# Exosomal circSPIRE1 mediates glycosylation of E-cadherin to suppress metastasis of renal cell carcinoma

**DOI:** 10.1038/s41388-023-02678-7

**Published:** 2023-04-12

**Authors:** Guannan Shu, Xuanxuan Lu, Yihui Pan, Junjie Cen, Kangbo Huang, Mi Zhou, Jun Lu, Jiaqi Dong, Hui Han, Wei Chen, Juan Lin, Junhang Luo, Jiaxing Zhang

**Affiliations:** 1 Department of Urology, First Affiliated Hospital, Sun Yat-sen University, Guangzhou, China; 2 Department of Food Science and Engineering, Jinan University, Guangzhou, China; 3 Department of Urology, Third Affiliated Hospital of Soochow University, Changzhou, China; 4 Department of Oncology, First Affiliated Hospital, Sun Yat-sen University, Guangzhou, China; 5 Department of Urology, Sun Yat-Sen University Cancer Center, Guangzhou, China; 6 Department of Pediatrics, Third Affiliated Hospital, Sun Yat-Sen University, Guangzhou, China

**Keywords:** Metastasis, Non-coding RNAs, RNA metabolism

## Abstract

Metastasis is the main cause of mortality in renal cell carcinoma (RCC). Circular RNAs (circRNAs) involvement in RCC metastasis has been described, although the underlying mechanisms remain unknown. We evaluated recurring lung-metastasis cases using patient-derived xenograft models and isolated a highly metastatic clone. CircSPIRE1 was identified as a metastasis-inhibiting circRNA in clinical cohort and xenograft models. Mechanistically, circSPIRE1 suppressed mesenchymal state through regulating ELAV like RNA binding protein 1-mRNA binding, and upregulating polypeptide N-acetylgalactosaminyltransferase 3 (*GALNT3*) and KH domain RNA binding protein (*QKI*) expression. GALNT3 promoted glycosylation and cytomembrane localization of E-cadherin. QKI formed a positive feedback loop to enhance circSPIRE1 expression. Meanwhile, exosomal circSPIRE1 suppressed angiogenesis and vessel permeability. Our work reveals a non-canonical route for circRNAs in RCC to suppress metastasis. Furthermore, a nanomedicine consisting of circSPIRE1 plasmid suppressed metastasis formation. In conclusion, circSPIRE1 may be a predictor of metastasis and a potential therapeutic target of metastatic RCC.

## Introduction

The incidence of kidney cancer continues to increase in both men and women, with renal cell carcinoma (RCC) representing approximately 85% of kidney cancers in recent decades [1, 2]. Due to advances in abdominal imaging and ultrasound technology, most RCCs are localized, but approximately 30% of cases still present metastases at the time of first diagnosis [3, 4]. Furthermore, up to one-third of patients develop local recurrence or distant metastases that are mostly resistant to chemotherapy and radiation therapy after surgical resection [5]. In metastatic RCC, there is limited effectiveness of targeted therapies such as sunitinib and pazopanib, because drug resistance develops between 6 and 15 months of treatment [6]. Because overall survival (OS) in patients remains unsatisfactory, exploration of the biological characteristics and the underlying mechanisms of metastatic RCC is a top priority.

Glycosylation is one of the most frequent protein modifications and has been associated with numerous pathologies, including cancer [7]. In cancer, glycosylation involves cell signaling, tumor invasion, cell-matrix interactions, angiogenesis, and metastasis formation [8]. E-cadherin is a transmembrane glycoprotein that functions as the main cell–cell adhesion molecule [9]. Previous studies have mainly focused on the impact of N-glycosylation and O-mannosylation alterations on malignant and invasive phenotypes [10, 11]. Abnormal O-glycosylation of E-cadherin has been proposed as a regulator that controls the localization, stability, and secretion of E-cadherin in trophoblast stem cells and in breast cancer cells [12]. However, the effects of altered O-glycosylation of E-cadherin have not been reported in RCC.

Circular RNAs (circRNAs) are endogenous closed-loop RNAs generated by back splicing of pre-mRNA [13]. Their circular form determines that circRNAs are resistant to exonuclease degradation and are more stable than linear RNA. To date, researchers have identified more than 100,000 unique circRNAs in Homo sapiens [14]. CircRNAs have been implicated in many pathological processes of human diseases, including malignant tumors [15]. Numerous potential functions, such as microRNA sponges, binding to RNA-associated proteins to form RNA-protein complexes, or translation have been reported [16]. So far, most reported circRNAs promote the development and progression of RCC through circRNA-microRNA-mRNA interaction networks [17]. The ability of circRNAs to act as scaffolds to facilitate RNA-protein interactions in RCC has not been thoroughly investigated, especially in metastatic RCCs.

In the present study, we constructed a patient-derived xenograft (PDX) model and identified a highly metastatic clone through the repeated formation of lung metastases in an in vivo model. Through next-generation sequencing, we identified a metastasis-inhibiting circRNA, hsa_circ_0000829 (circSPIRE1), which is downregulated in RCC patients with metastasis and predicts better patient survival. In vivo and in vitro assays confirmed that circSPIRE1 is a suppressor of metastasis. We used bioinformatic algorithms to preliminarily predict and molecular experiments to validate the functional sites of circSPIRE1. We determined that circSPIRE1 served as a scaffold between the ELAV-like RNA binding protein 1 (*ELAVL1*) and acetylgalactosaminyltransferase 3 (*GALNT3*) mRNA, as well as the KH domain RNA binding protein (*QKI*) mRNA, which leads to upregulation of GALNT3 and QKI. GALNT3 promoted the O-GalNAc glycosylation of E-cadherin and suppressed the mesenchymal state. QKI, in turn, was able to bind to introns flanking circSPIRE1 forming exons that promoted circSPIRE1 expression, generating a positive feedback loop to maintain the epithelial state. Furthermore, we found that circSPIRE1 could be packed in exosomes and transferred to endothelial cells to suppress vascular permeability and angiogenesis. Based on these findings, we developed a nanomedicine consisting of chitosan-epigallocatechin gallate nanoparticles (CS-EGCG NPs) which were able to deliver the circSPIRE1 overexpression plasmid to tumor cells and thereby, successfully suppressing metastasis in vivo.

## Results

### Establishment of a patient-derived metastatic RCC xenograft model and profiling of dysregulated circRNAs in RCC tissues

**Fig. 1:**
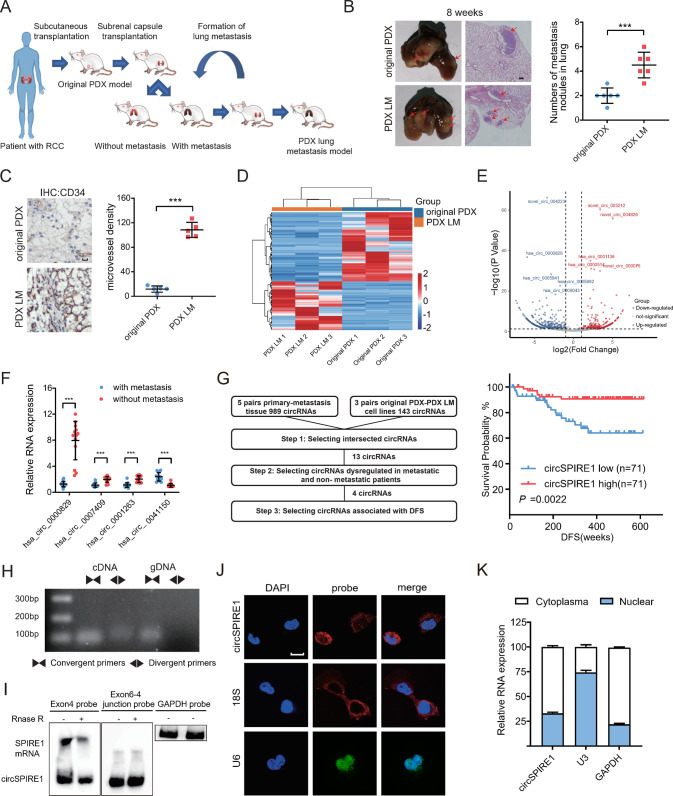
Downregulation and characterization of circSPIRE1 in metastatic RCC. **A** Schematic illustration of the development of the metastatic RCC PDX model, PDX LM. **B** Increased tumor metastasis formed in lungs of PDX LM model compared to the original PDX model. Left, representative lung and representative HE staining of lung metastatic lesions, scale bar, 100 µm. Right, the number of metastatic nodules formed in the lungs of mice for each group (*n* = 6 mice/group). Data represent mean ± S.D.; dot plot reflects data points from independent experiments. The *P* values were determined by Student’s *t* test. **C** Increased microvessel density of the PDX LM model compared to the original PDX model. Left, representative tumor stained with *CD34* anti-body. Right, microvessel density for each group (*n* = 5 mice/group). Data represent mean ± S.D.; dot plot reflects data points from independent experiments. The *P* values were determined by Student’s *t* test. **D** Heatmap of high-throughput sequencing between PDX LM cells and original PDX cells. **E** Volcano plot of circRNAs comparing five no clinically metastasis cases and five metastasis cases. **F** qRT-PCR analysis of four circRNAs expression in 14 RCC patients with metastasis and 14 patients without metastasis. Data represent mean ± S.D.; The *P* values were determined by Student’s *t* test. **G** Left, flowchart illustrating the screening criteria of potential metastasis related circRNAs. Right, Kaplan−Meier analysis of DFS in RCC patients with low versus high expression of circSPIRE1. The *P* value was determined by a Log-rank test. **H** Existence of circSPIRE1 confirmed by RT-PCR and gel electrophoresis using convergent and divergent primers. CircSPIRE1 can only be amplified in cDNA. **I** Northern blotting of circSPIRE1 and SPIRE1 transcripts by hybridization with exon 4 (left) and exon 6−exon 4 junction (right) probes in the presence or absence of RNase R treatment. **J** RNA fluorescence in situ hybridization for circSPIRE1. Scale bar, 10 µm. **K** Cytoplasmic and nuclear RNA fractionation experiment showing that circSPIRE1 mainly localized in the cytoplasm. *GAPDH* and U3 as positive controls in the cytoplasm and nucleus, respectively.

Because metastasis is the main reason for recurrence observed in patients with RCC, which ultimately leads to mortality, our aim was to identify the circRNAs that were responsible for RCC metastasis. We applied a screening assay based on the repeated formation of lung metastasis in an in vivo xenograft model to mimic the invasion-metastasis step and isolated a clone with higher lung metastatic potential than the original PDX model. We termed this clone, the patient-derived xenograft (PDX) with lung metastasis (PDX LM) clone (Fig. 1A). The increased invasive ability of this clone was confirmed by in vivo and in vitro assays (Figs. 1B, S1A). PDX LM maintained the clear cell phenotype despite passaging in mice, with a high incidence (>90%) of lung metastases formation at each passage, producing a higher density of tumor vessels stained with CD34 (Fig. 1C). Furthermore, human Alu sequences were detected by polymerase chain reaction (PCR), which suggested that the tumors, though passaged in mice, were of human origin (Fig. S1B). Next-generation sequencing (NGS) was carried out to identify deregulated circRNAs in PDX LM and original PDX cells. The resulting expression showed a marked difference between the original PDX and PDX LM cells (Fig. 1D).

After analysis of differentially expressed genes, 59 upregulated and 84 downregulated circRNAs with |log2 (fold change) |>1, and *P* value < 0.05 were selected. High-throughput RNA sequencing comparing five no clinically metastasis cases and five metastasis cases was performed to identify candidates based on the |log2 (fold change) |>1, and *P* value < 0.05 cutoff values and graphed using a volcano plot (Fig. 1E). Collectively, thirteen dysregulated circular RNAs were selected as validation candidates (Fig. S1C, Supplementary data 1). Furthermore, we collected 14 RCC patients with metastasis, as well as 14 patients without metastasis, and the expression levels of 13 circRNAs were quantified through real-time quantitative polymerase chain reaction (RT-qPCR) in primary tumor tissues. We found that three circRNAs were downregulated and one circRNA was statistically upregulated in metastatic RCC compared to RCC without metastases (Fig. 1F, Supplementary Data 2). To investigate the relevance between these four circRNAs and clinical outcome, a cohort of 142 patients with RCC with clinical information was collected (Supplementary Data 3). Among the four candidates, we found that only a lower expression of hsa_circ_0000829 (Chr18: 12506475-12535600) was significantly correlated with poorer disease-free survival (DFS) (Fig. 1G). To validate the clinical relevance of hsa_circ_0000829 in RCC, we applied qRT–PCR to detect the expression of hsa_circ_0000829 in 142 pairs of RCC tissues and matched adjacent normal tissues. It was identified that hsa_circ_0000829 was significantly downregulated in RCC tissue than adjacent normal tissue (Fig. S1D). Furthermore, the lower expression of hsa_circ_0000829 was statistically associated with the advanced clinical TNM stage and the Fuhrman grade (Table S1). According to the human reference genome (GRCh37/hg19), hsa_circ_0000829 is derived from exon 4, exon 5, and exon 6 regions within the spire-type actin nucleation factor 1 (*SPIRE1*) locus. Hence, it was named circSPIRE1. Univariate and multivariate Cox analysis confirmed that the low expression of circSPIRE1 was correlated with poor patient DFS in our RCC cohort (Table S2). In addition, receiver operating characteristic (ROC) analysis found that circSPIRE1 level can improve the DFS predictive ability of the clinical prognostic score algorithm (SSIGN scoring scale) (Fig. S1E). Therefore, we speculated that circSPIRE1 expression was associated with RCC metastasis.

### Characterization of circSPIRE1 in RCCs

We compared circSPIRE1 expression levels in five RCC cell lines (PDX LM, original PDX, 786-O, 769-P, ACHN) as well as the HK-2 cell line, and found a clear downregulation of circSPIRE1 in RCC cell lines, especially in PDX LM cells compared to HK-2 cells (Fig. S1F). CircSPIRE1 could be amplified with divergent primers from complementary DNA (cDNA), but not from genomic DNA (gDNA); moreover, convergent primers could amplify not only cDNA but also gDNA (Fig. 1H). Furthermore, we examined the junction sequence of circSPIRE1 by RT-PCR using divergent primers. Through Sanger sequencing of the PCR products, we confirmed the existence of a specific splice junction in circSPIRE1 (Fig. S1G). Tolerance under treatment with RNase R confirmed that circSPIRE1 is a covalently closed circular RNA, while *SPIRE1* mRNA was sensitive upon digestion with this treatment (Fig. S1H). The qRT-PCR analysis suggested a longer half-life of circSPIRE1 in RCC cells compared to *SPIRE1* mRNA after treatment with actinomycin D, a transcription inhibitor (Fig. S1I). Subsequently, we used probes that could hybridize with the specific junction to recognize circSPIRE1, and probes hybridizing with exon 4 to recognize both *SPIRE1* mRNA and circSPIRE1, through northern blotting (Fig. 1I). Furthermore, the cytoplasmic localization of circSPIRE1 was confirmed through nuclear and cytoplasmic fractionation and fluorescence in situ hybridization (FISH) (Fig. 1J, K). These results confirmed that circSPIRE1 was a classic cytoplasmic circRNA expressed in RCC.

### CircSPIRE1 suppressed RCC cell migration in vitro and metastasis in vivo

**Fig. 2:**
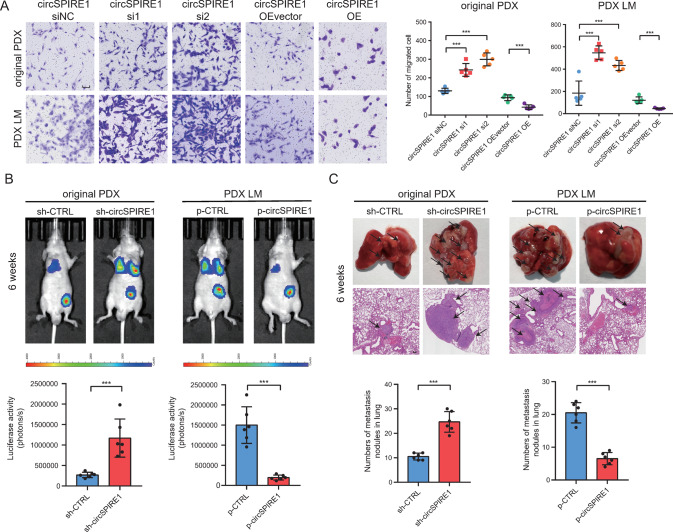
Function assays of circSPIRE1 in RCC cells. **A** Transwell assays showing that expression of circSPIRE1 affected the migration ability of RCC cells. Left, transwell representative images. Scale bar, 100 µm. Right, numbers of migration cell (*n* = 5/group). **B** Bioluminescent in the lungs of mice through sub-capsular injection showing that expression of circSPIRE1 affected RCC metastasis in vivo. Top, representative bioluminescent images of lungs for each experimental group. Bottom, statistical analysis of bioluminescent tracking plots (*n* = 6 mice/group). **C** Tumor metastasis formed in the lungs of mice through sub-capsular injection showing that expression of circSPIRE1 affected RCC metastasis in vivo. Top, representative lung and representative HE staining of lung metastatic lesions. Scale bar, 100 µm. Bottom, statistical analysis of the number of metastatic nodules formed in the lungs of mice for each group (*n* = 6 mice/group). **B**, **C** Data represent mean ± S.D.; dot plot reflects data points from independent experiments. The *P* values were determined by Student’s *t* test.

Due to the clinical relevance of circSPIRE1 levels and RCC aggressiveness, we conducted both in vitro and orthotopic in vivo functional assays to evaluate the role of circSPIRE1 in RCC cell metastasis. We constructed siRNAs targeting the back-splice region of circSPIRE1 (circSPIRE1 si) or the circSPIRE1 overexpression plasmid (circSPIRE1 OE) to knockdown or overexpress circSPIRE1. Transwell assays and wound healing assays revealed that tumor cell migration ability was enhanced following circSPIRE1 knockdown. In contrast, overexpressed circSPIRE1 suppressed cell migration (Figs. 2A, S1J). To evaluate the metastasis suppressing effect of circSPIRE1 in vivo, we transfected original PDX cells with an shRNA targeting the circSPIRE1 junction site (original PDX sh-circSPIRE1), while PDX LM cells were stably transfected with the circSPIRE1 plasmid (PDX LM p-circSPIRE1). Then, in vivo lung metastasis models were established through sub-capsular injection of stably transfected cells. We observed that tumor metastatic nodes in lungs were remarkably reduced in the PDX LM p*-*circSPIRE1 group compared to control cells. The opposite effect was observed in the original PDX sh-circSPIRE1 group (Fig. 2B, C). These results demonstrated that circSPIRE1 suppressed RCC invasion and metastasis ability.

### CircSPIRE1 acted by binding to the ELAVL1 protein

**Fig. 3:**
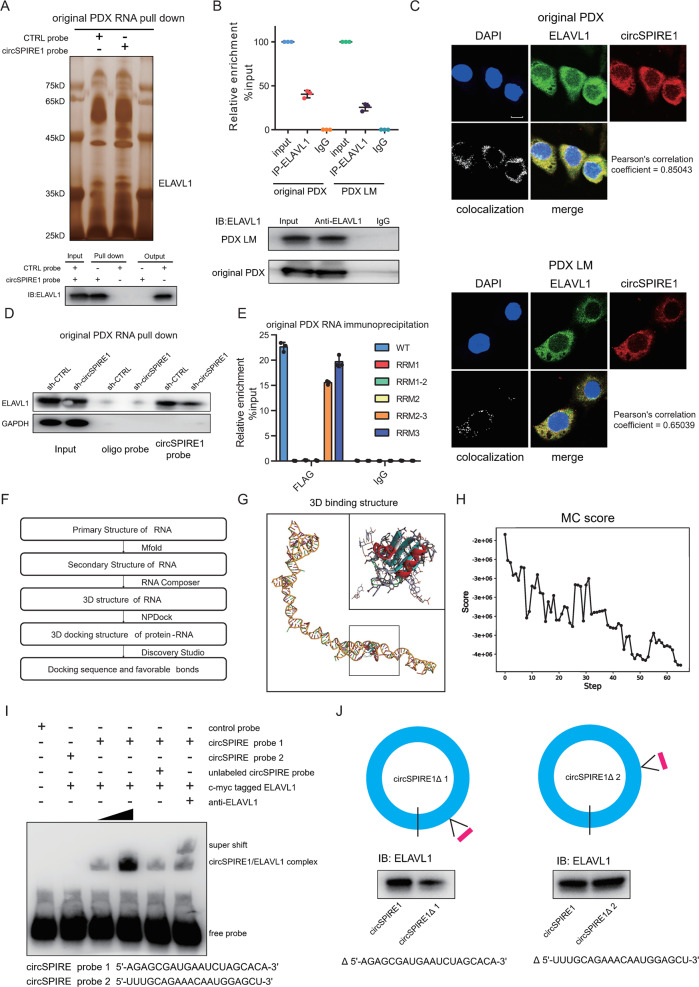
5ʹ-AGAGCGAUGAAUCUAGCACA-3ʹ sequence of circSPIRE1 interacts with RRM3 domain of ELAVL1. **A** Top, identification of the circSPIRE1-protein complex pulled down by circSPIRE1 junction probe with protein extracts from original PDX cells. Bottom, immunoblot analysis of ELAVL1 after pulldown assay showing its specific association with circSPIRE1. **B** RIP assays showing the association of ELAVL1 with circSPIRE1. Relative enrichment representing RNA levels associated with ELAVL1 compared to an input control. IgG antibody served as a control. Data represent mean ± S.D.; dot plots reflect data points from three independent experiments. **C** IF-FISH assay showing that circSPIRE1 is colocalized with ELAVL1 protein in the cytoplasm. Scale bar, 10 µm. **D** Western blotting assay showing the level of ELAVL1 protein pulled down by biotin-labeled circSPIRE1 probes from the lysates of original PDX cells following transfection with shRNAs targeted circSPIRE1 and negative control. **E** Relative enrichment representing circSPIRE1 levels associated with truncated ELAVL1 relative to an input control. Data represent mean ± S.D.; dot plots reflect data points from three independent experiments. **F** Flowchart illustrating the computational analysis of circSPIRE1 interaction with ELAVL1. **G** Graphical representation of the best scored three-dimensional structures of circSPIRE1 and ELAVL1 (RRM3 domain) docking models with a zoom-in image. **H** Refinement of the best docked circSPIRE1-ELAVL1 (RRM3 domain) model showing MC score vs steps of simulation. **I** RNA-EMSA assay showing the binding ability of purified ELAVL1 with two biotin-labeled oligonucleotides containing two predicted sequence from circSPIRE1. **J** Western blotting assay showing the level of ELAVL1 protein pulled down by biotin-labeled circSPIRE1 probes from the lysates of 293 T following transfection with circSPIRE1 full-length plasmid or truncated circSPIRE1 plasmid.

To discover the underlying role of circSPIRE1 in RCC, we first used TransCirc [18], a specialized database that provides comprehensive evidence supporting the translation potential of circular RNAs, to determine whether circSPIRE1 had the ability to translate. Although an ORF and eight IRES sequences were detected, proteomics evidence by mass spectrometry, ribosome/polysome profiling, and translation initiation site analysis did not support the translation potential of circSPIRE1. Recently, it has been reported that circRNA-protein interactions may play a role in the progression of various cancers [19]. Thus, we performed RNA pull-down and mass spectrometry (MS) analysis to identify proteins that might interact with circSPIRE1. Based on RNA pull-down assays, we observed that ELAVL1 protein was enriched in complexes pulled down by probes targeting the circSPIRE1 junction site (Fig. 3A, Table S3). Subsequently, we validated the interaction between the ELAVL1 protein and circSPIRE1 by immunoprecipitating RNA in original PDX and PDX LM cell lines (Fig. 3B). Then, through immunofluorescence and fluorescence in situ hybridization (IF-FISH) assays, we confirmed that circSPIRE1 and ELAVL1 co-localized in the cytoplasm (Fig. 3C). Quantifications of colocalization are provided in Table S4. Furthermore, we performed another RNA pull-down assay with cells transfected with shRNAs targeting circSPIRE1 and a negative control. The results showed that knockdown of circSPIRE1 did not influence the total amount of ELAVL1 protein, but reduced the ELAVL1 protein pulled down by circSPIRE1 (Fig. 3D). These results suggested that ELAVL1, an RNA binding protein (RBP), could bind circSPIRE1 in the cytoplasm.

Next, our objective was to discover which domain of ELAVL1 was responsible for the interaction with circSPIRE1. We constructed ELAVL1 mutants with truncation of individual RNA recognition motif (RRM) domains (Fig. S2A). RBP immunoprecipitation (RIP) assays in original PDX cells revealed that the RRM3 domain of ELAVL1 specifically bound to circSPIRE1 (Fig. 3E). Furthermore, we then investigated which sequence in circSPIRE1 was critical for RNA-protein binding. We performed a computational analysis of circSPIRE1 interaction with ELAVL1 (Fig. 3F). To determine the secondary structures of circSPIRE1, we used the minimum free energy algorithm implemented in Mfold (version2.3) [20]. The secondary structure with a lower theoretical value of free energy was selected as the model structure for the prediction of the three-dimensional (3D) structure (Fig. S2B). The output file containing a primary sequence and an associated secondary structure (Dot-Bracket Notation) was then submitted to RNA Composer to generate the 3D structure [21, 22] (Fig. S2C). The docking procedure between the circSPIRE1 and ELAVL1 RRM3 domain was carried out by the NPDock web server [23]. The 3D crystal structure of RRM3 domain was derived from Protein Data Bank entry 6GC5 [24]. The molecular docking output included the three best scored structures that were taken from the three largest clusters. The best and second-best scored structures shared the same interacting sequence of circSPIRE1, 5ʹ-AGAGCGAUGAAUCUAGCACA-3ʹ (Fig. 3G). And the third structure suggested that 5ʹ-UUUGCAGAAACAAUGGAGCU-3ʹ was critical in the docking procedure (Fig. S2D). The contact distance between circSPIRE1 and the ELAVL1 binding domain (Table S5), and the MC score (Fig. 3H) both supported the conclusion that circSPIRE1 sufficiently docked ELAVL1. All favorable bonds were acquired through Discover Studio.

We then aimed to verify the in silico binding through in vitro RNA-EMSA. We confirmed that the best- and second-best scored structures sharing the same binding sequence were indispensable for RNA-protein binding. The supershift bands suggested that ELAVL1 specifically bound to the former sequence (Fig. 3I). Upon increasing the concentration of the ELAVL1 protein, the concentration of the circSPIRE1/ELAVL1 complexes was also increased. Further evidence that the ELAVL1 protein binds to the predicted sequence was obtained by employing constructs that overexpressed full-length circSPIRE1 or truncated circSPIRE1 lacking the former (circSPIRE1Δ1) or latter (circSPIRE1Δ2) predicted binding sequence. We transfected the constructs into 293 T cells, and 48 h later performed RNA pull-down assays. In line with earlier results, ELAVL1 was more enriched in pulldowns from cultures expressing full-length circSPIRE1 than in cultures expressing circSPIRE1Δ1, while circSPIRE1Δ2 showed no effect on binding of ELAVL1/circSPIRE1 (Fig. 3J). Altogether, our data revealed that ELAVL1 bound to circSPIRE1 through the RRM3 domain.

### CircSPIRE1 guided ELAVL1 binding and stabilized *GALNT3*/*QKI* mRNA

**Fig. 4:**
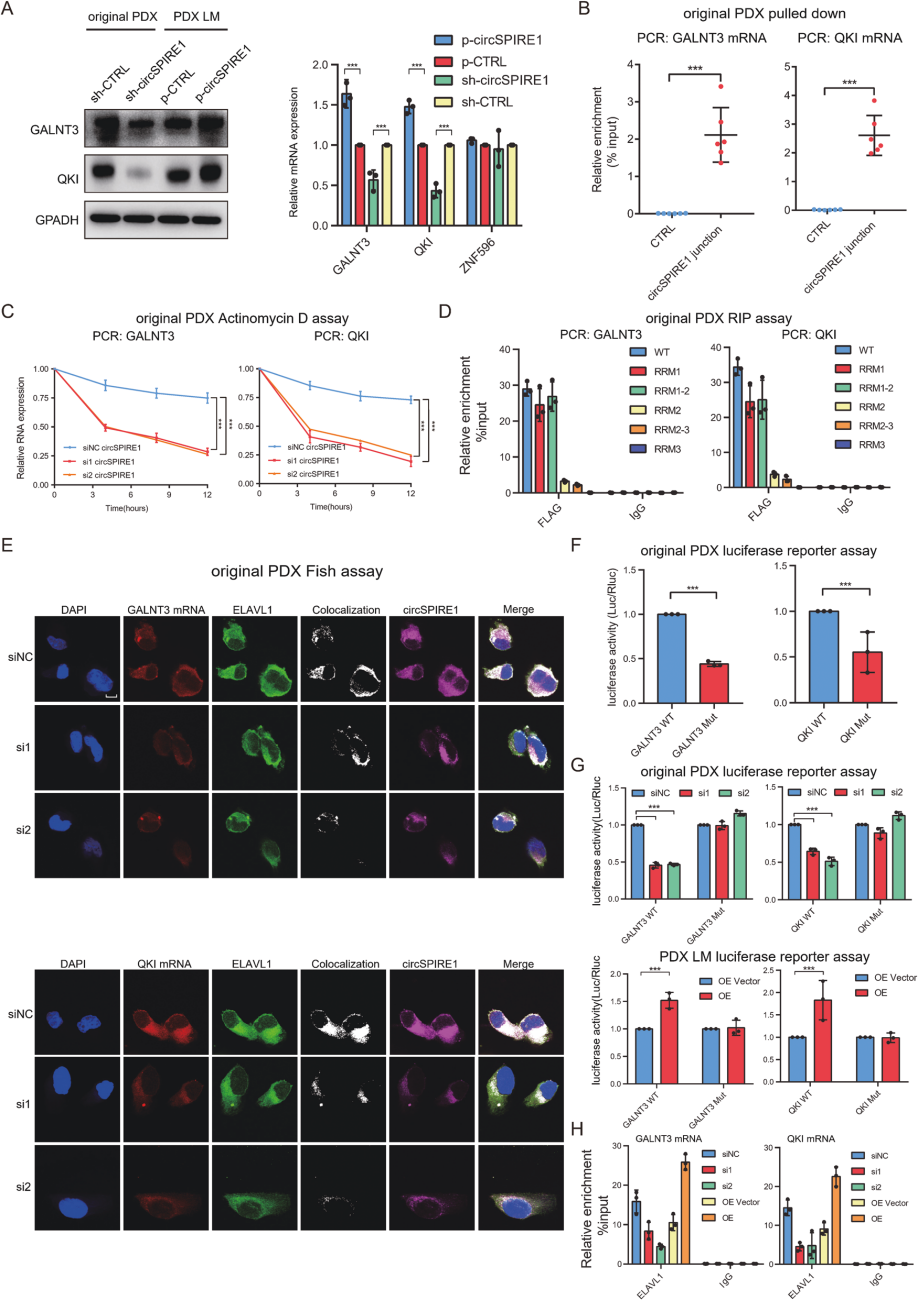
CircSPIRE1/ELAVL1/mRNA ternary complex stabilizes GALNT3 and QKI mRNA. **A** Western blotting (left) as well as qPCR (right) validation of *GALNT3* and *QKI* upon circSPIRE1 knockdown and overexpression. **B** Relative enrichment representing *GALNT3* mRNA (left) and *QKI* mRNA (right) levels associated with circSPIRE1 junction compared to controls. Data represent mean ± S.D.; dot plots reflect data points from three independent experiments. The *P* values were determined by Student’s *t* test. **C** Reduced stability of *GALNT3* (left) and *QKI* (right) upon circSPIRE1 knockdown. **D** Relative enrichment representing the enrichment of *GALNT3* mRNA (left) and *QKI* mRNA (right) associated with the truncated ELAVL1 protein complex compared to an input control. IgG antibody served as a control. Data represent mean ± S.D.; dot plots reflect data points from three independent experiments. **E** IF-FISH assay showing that the colocalization of circSPIRE1/ELAVL1/mRNA was decreased upon knockdown of circSPIRE1. Scale bar, 10 µm. **F** Relative luciferase activity of luciferase reporter gene with *GALNT3*-WT or *GALNT3*-Mut (left) and *QKI*-WT or *QKI*-Mut (right). Data represent mean ± S.D.; dot plots reflect data points from three independent experiments. The *P* value was determined by Student’s *t* test. **G** Upon knockdown or overexpression of circSPIRE1, relative luciferase activity of luciferase reporter gene with *GALNT3*-WT or *GALNT3*-Mut (left) and *QKI*-WT or *QKI*-Mut (right). Data represent mean ± S.D.; dot plots reflect data points from three independent experiment. The *P* value was determined by Student’s *t* test. **H** Upon knockdown or overexpression of circSPIRE1, relative enrichment representing *GALNT3* mRNA (left) and *QKI* mRNA (right) levels associated with ELAVL1 protein complex compared to an input control. IgG antibody served as a control. Data represent mean ± S.D.; dot plots reflect data points from three independent experiments.

*ELAVL1* has been reported to be a conventional RBP, which selectively binds AU-rich elements (AREs) found in the 3ʹ untranslated regions of mRNAs and plays an important role in mRNA stability [25–27]. Thus, we wondered whether the circSPIRE1/ELAVL1 complex exerts activity by protecting downstream targets from fast degradation. We performed RNA sequencing analyses using PDX LM p-CTRL cells versus PDX LM p-circSPIRE1 cells. A total of 283 upregulated mRNAs were identified upon overexpression of circSPIRE1 (fold change>2, *p* < 0.05) (Table S6). Next, among the 283 upregulated mRNAs, we screened ELAVL1-binding 3ʹUTRs obtained from the StarBase across different cancer types [28]. Eight mRNAs were identified by intersecting the results of the RNA sequencing analyses and the StarBase output. Given that circSPIRE1 suppresses metastasis, we applied data sets from The Cancer Genome Atlas (TCGA: https://www.cancer.gov/tcga) to determine the impact of these eight mRNAs respectively on the DFS of RCC patients. Based on the above results, *GALNT3*, *QKI*, and *ZNF596* mRNA levels were negatively correlated with disease progression in RCC, and were therefore identified as potential downstream targets of circSPIRE1/ELAVL1 (Fig. S2E). By utilizing circRNA and polyA sequencing data acquired from five no clinically metastasis cases and five metastasis cases, we found that the expression of *QKI* mRNA (NM_006775.2) and *GALNT3* mRNA (NM_004482.3) was positively correlated with the expression of circSPIRE1 (Fig. S2F). We confirmed that *GALNT3* and *QKI* were abundantly expressed in RCC cell line, and targets of circSPIRE1 through qPCR and western blotting validation (Figs. 4A, S2G). Subsequently, through sequence BLAST analysis, we found that the AAACA site as well as the GUAAUUG site of circSPIRE1 could separately bind to the 3′UTR region of *GALNT3* mRNA at the UGUUU site and the CAUUAAC site of *QKI* mRNA (Fig. S3A). The interaction was confirmed by separate RNA pull-down assays (Fig. 4B).

Next, we found that the downregulation of circSPIRE1 significantly suppressed the stability of *GALNT3* mRNA and *QKI* mRNA in original PDX cell line (Fig. 4C). While overexpression of circSPIRE1 enhanced the stability of *GALNT3* mRNA and *QKI* mRNA in PDX LM cell line (Fig. S3B). Consistent with previous studies [29] which reported that RRM1 was the primary ARE-binding domain in ELAVL1 and that RRM2 improved the RNA-binding affinity of ELAVL1, our RIP assays with ELAVL1 mutants confirmed that both RRM1 and RRM2 were responsible for ELAVL1 and mRNA binding (Fig. 4D). We further confirmed circSPIRE1 guides ELAVL1 to bind and stabilize *GALNT3*/*QKI* mRNA through the following experimental evidence. First, RNA-FISH assays showed that *GALNT3* mRNA, as well as *QKI* mRNA and ELAVL1 were colocalized in the cytoplasm. When circSPIRE1 was knocked down, the colocalization of ELAVL1 and downstream mRNA was significantly decreased, while the expression of ELAVL1 protein remained unaffected in original PDX cell line (Fig. 4E, Table S4). While overexpression of circSPIRE1 enhanced the colocalization of ELAVL1 protein and downstream mRNA in PDX LM cell line (Fig. S3C). Second, we constructed two sets of luciferase reporter minigenes including wild-type *GALNT3* (*GALNT3*-WT) and wild-type *QKI* (*QKI*-WT), and mutant *GALNT3* (*GALNT3*-Mut) and mutant *QKI* (*QKI*-Mut), respectively. The mutant form of the luciferase reporter contained the mutant binding sequence instead of the sequence required for the interaction. The luciferase activity of *GALNT3*-Mut was approximately 50% that of *GALNT3*-WT. The luciferase activity of *QKI*-Mut was also obviously weaker compared to *QKI*-WT (Fig. 4F). Furthermore, circSPIRE1 knockdown significantly suppressed the expression of luciferase mRNA and luciferase activity of wild-type luciferase reporters, rather than mutant luciferase reporters. In contrast, overexpression of circSPIRE1 significantly elevated the expression of luciferase mRNA and luciferase activity of wild type luciferase reporters, but not that of mutant luciferase reporters (Fig. 4G). Third, the knockdown of circSPIRE1 in original PDX cells dramatically decreased the ELAVL1/mRNA protein-RNA complex, as shown in the RIP assays, in contrast, overexpression of circSPIRE1 in PDX LM cells significantly increased the enrichment of mRNA in immunoprecipitated complexes of ELAVL1 (Fig. 4H). These results demonstrated that circSPIRE1 was vital in enhancing the interactions between ELAVL1 and *GALNT3* mRNA, as well as *QKI* mRNA, by forming a ternary complex of circSPIRE1/ELAVL1/mRNA.

### *GALNT3* suppressed the mesenchymal state in RCC cells by promoting O-GalNAc glycosylation of E-cadherin

**Fig. 5:**
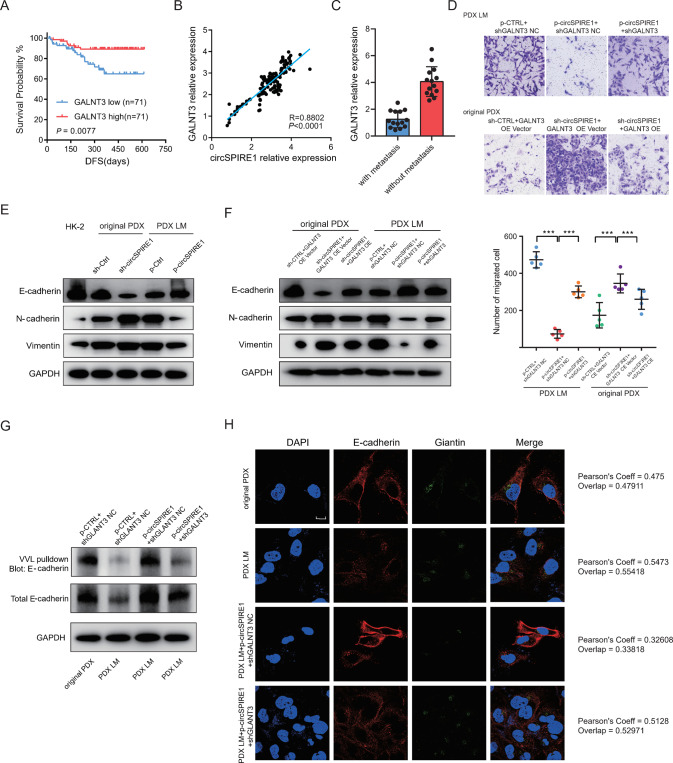
CircSPIRE1 suppresses EMT of RCC cells through stabilizing GALNT3 mRNA. **A** Kaplan−Meier analysis of DFS in RCC patients with low versus high expression of *GALNT3*. The *P*-value was determined by the Log-rank test. **B** Positive correlation between *GALNT3* expression and circSPIRE1 expression in 142 clinical samples of RCC. **C** qRT-PCR analysis of *GALNT3* expression in 14 RCC patients with metastasis and 14 patients without metastasis. Data represent mean ± S.D.; the *P* values were determined by Student’s *t* test. **D** Transwell assays showing that circSPIRE1 suppresses the migration ability of RCC cells depending on *GALNT3*. Top, representative images of the Transwell assay. Scale bar, 100 µm. Bottom, numbers of migrating cells (*n* = 5/group). **E** Western blots of epithelial and mesenchymal markers upon overexpression of circSPIRE1 in PDX LM and knockdown of circSPIRE1 in original PDX. **F** Western blots of enhanced epithelial markers upon overexpression of circSPIRE1 was reversed by *GALNT3* knockdown in PDX LM and reduced epithelial markers upon knockdown of circSPIRE1 was reversed by *GALNT3* overexpression in original PDX. **G** E-cadherin O-GalNAc glycosylation decreases in PDX LM comparing with original PDX and increases upon circSPIRE1 overexpression. Knockdown of *GALNT3* reversed the effect of circSPIRE1 overexpression. **H** E-cadherin retention in the Golgi increases in PDX LM compared to original PDX and decreases upon circSPIRE1 overexpression. Knockdown of *GALNT3* reversed the effect of circSPIRE1 overexpression. Scale bar, 10 µm. Pearson’s coeff was used as quantifications.

To further confirm that *GALNT3* is essential in the metastasis of RCC, we first examined the expression of *GALNT3* in a cohort of 142 patients with RCC, and consistent with the result obtained from the TCGA data set, low *GALNT3* expression predicted a poorer DFS of patients with RCC (Fig. 5A). Second, qPCR results of 142 clinical samples of RCC demonstrated a positive correlation between *GALNT3* expression and circSPIRE1 expression (Fig. 5B). Third, lower expression of *GALNT3* was confirmed in our 14 patients with metastasis compared to patients without metastasis (Fig. 5C). Fourth, Transwell assays demonstrated that *GALNT3* knockdown reversed the reduction in migration induced by circSPIRE1 overexpression in PDX LM cells in vitro, while *GALNT3* overexpression rescued the enhanced migration induced by circSPIRE1 knockdown in original PDX cells (Fig. 5D).

As previously reported, the loss of *GALNT3* induces epithelial mesenchymal transition (EMT) and disrupts barrier formation [12]. Thus, we used Gene Set Enrichment Analysis (GSEA: https://www.gsea-msigdb.org/gsea/index.jsp) to predict the pathways involved downstream of *GALNT3*, and the analysis suggested that EMT was a potentially involved pathway (Fig. S3D). Thus, we wondered whether circSPIRE1 could suppress EMT in RCC cells by stabilizing *GALNT3* mRNA. First, original PDX cells and PDX LM cells displayed a mesenchymal-like phenotype with loss of cell-cell adhesion and gain of front-back polarity, while HK-2 cells displayed an epithelial phenotype. And with the decrease of *GALNT3* expression in PDX LM comparing original PDX cells, mesenchymal-like phenotype was enhanced. (Fig. S3E). Next, overexpression of circSPIRE1 in PDX LM cells reduced the expression of mesenchymal markers and regained the expression of epithelial markers and knockdown of circSPIRE1 in original PDX cells led to the opposite effect (Fig. 5E). Finally, the elimination of *GALNT3* mRNA in PDX LM cells successfully reversed the effect of circSPIRE1 overexpression, which led to the acquisition of epithelial markers and *GALNT3* overexpression in original PDX cells rescued the effect of circSPIRE1 knockdown (Fig. 5F). Altogether, these data indicated that circSPIRE1 was critical for sustaining the epithelial state in RCC cells by upregulating *GALNT3*.

Because GALNT3 protein functions as an O-GalNAc glycosyltransferase localized to the Golgi and changes in O-GalNAc levels affect the location of E-cadherin and alter the metastatic phenotype [12], we carried out an O-glycosylated glycan-binding lectin VVL biotin pulldown assay and analyzed proteins by western blotting. There was a clear downregulation of O-GalNAc glycosylated E-cadherin in PDX LM compared to the original PDX cells, which is consistent with a higher metastatic potential of PDX LM compared to the original PDX that we verified by the functional assays described above. In addition, VVA-precipitated E-cadherin was upregulated in circSPIRE1-ovexpressing cells. This effect was reversed by transfection of *GALNT3* shRNA (Fig. 5G). Immunofluorescence assays showed that E-cadherin localized more densely in the Golgi bodies of PDX LM cells compared to the original PDX cells where E-cadherin localized more at the cell surface membrane. Overexpressed circSPIRE1 led to the translocation of E-cadherin from Golgi to the cell surface, while *GALNT3* knockdown reversed this effect. The Golgi apparatus was labeled with a Golgi marker, Giantin (Fig. 5H, Table S4). Together, these data indicated that circSPIRE1 promotes O-GalNAc glycosylation of E-cadherin through *GALNT3*. O-GalNAc glycosylation is indispensable for the correct localization of E-cadherin, which suppresses RCC metastasis.

### *QKI* promoted the expression of circSPIRE1 through binding sites in introns

**Fig. 6:**
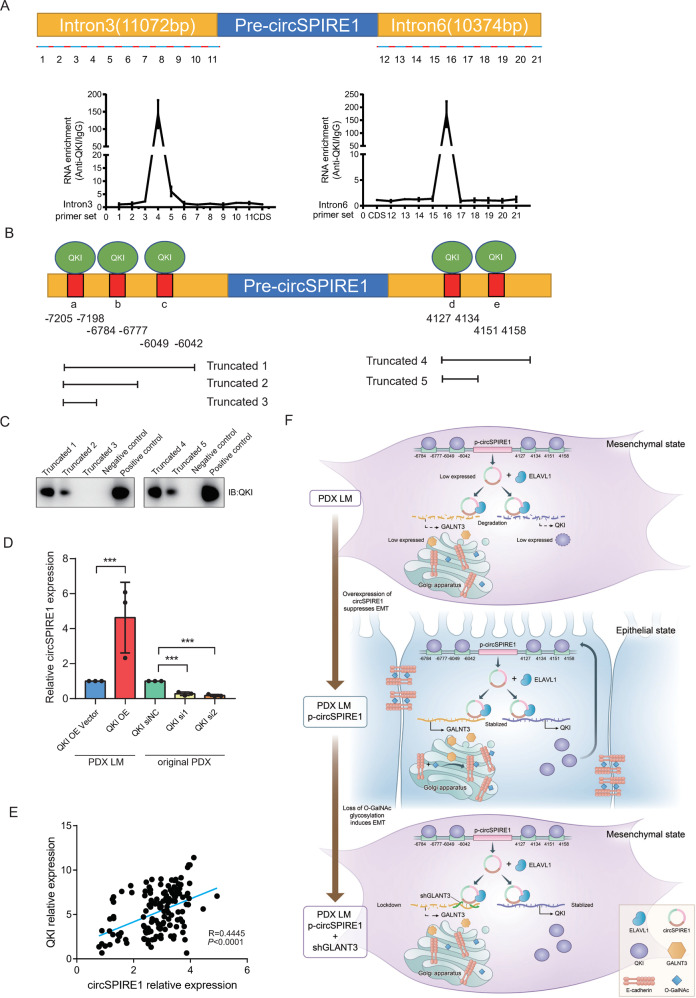
CircSPIRE1 expression was dependent on the QKI positive feedback loop. **A** QKI was associated with the introns flanking circSPIRE1. RIP assays were conducted with a QKI antibody or control IgG, and primer sets covering the introns flanking circSPIRE1 (top). RNA enrichment indicates the levels of the indicated fragments in the anti-*QKI* precipitates relative to those in the IgG precipitates (bottom). Mean ± S.D. are provided. Dot plots reflect data points from three independent experiments. **B** Schematic structures showing RNA-binding sequence in the introns flanking the circSPIRE1 and a summary of intron truncations. **C** RNA pull-down assay performed to analyze the interaction between QKI and introns flanking circSPIRE1. **D** CircSPIRE1 expression upon QKI knockdown or overexpression. **E** Positive correlation between *QKI* expression and circSPIRE1 expression in 142 clinical samples of RCC. **F** Schematic diagram of mechanism of circSPIRE1 during RCC metastasis in RCC cells.

QKI protein binds to the flanking sequence of circRNA-forming exons to promote circularization of circRNAs; thus, we postulated that QKI could identify QKI binding sites in the introns upstream and downstream of the circSPIRE1 forming exons [30]. To assess whether QKI binds to SPIRE1 pre-mRNA, we first used MEME SUITE [31] tools for motif discovery and searching, to recognize the QKI binding motif in intron 3 and intron 6 (Tables S7, S8). Next, formaldehyde crosslinked RIP assays with a QKI antibody were performed and the amount of circSPIRE1 pre-mRNA was detected by quantitative real-time PCR with a series of primers covering the entire intron 3, intron 6 and three exons that form circSPIRE1. Compared to the IgG control, one fragment within intron 3 and one fragment within intron 6, which were detected respectively by primer sets 4 and 16, were significantly enriched in anti-QKI immunoprecipitants (Fig. 6A). This result suggested that two regions located between primer sets 3–5 and primer sets 15–17, instead of circSPIRE1 itself, were associated with QKI. The two results above identified three putative elements that were located upstream (i.e., a, b, and c) and two were located downstream of the circRNA-forming splice sites, (i.e., d and e) (Fig. 6B). Next, we constructed five truncated transcripts that contained different QKI binding elements and performed an RNA pull-down assay. The results revealed that sequences b and c located upstream, as well as sequences d and e located downstream, which contain QKI binding elements, were important for the interaction between *SPIRE1* pre-mRNA and QKI (Fig. 6C). We then knocked down the expression of *QKI* and found a reduction in circSPIRE1 expression (Fig. 6D). Further, positive correlation between *QKI* expression and circSPIRE1 expression was demonstrated through qPCR of 142 clinical samples of RCC (Fig. 6E).

Together, these data confirmed our postulation that QKI could bind upstream and downstream of the circRNA-forming exons in *SPIRE1* pre-mRNA to promote circRNA formation. Furthermore, circSPIRE1 could form a ternary complex of circSPIRE1/ELAVL1/*QKI* mRNA to promote the expression of QKI protein, which together formed a positive feedback loop to maintain circSPIRE1 expression. In summary, circSPIRE1 functions as a scaffold between *ELAVL1* and downstream mRNA, *GALNT3*, as well as *QKI*. GALNT3 maintains the correct localization of E-cadherin, suppresses EMT. QKI bound to introns flanking circSPIRE1 forming exons to form a positive feedback loop to maintain expression of circSPIRE1 (Fig. 6F).

### CirSPIRE1 secreted by RCC was transferred to endothelial cells through exosomes

**Fig. 7:**
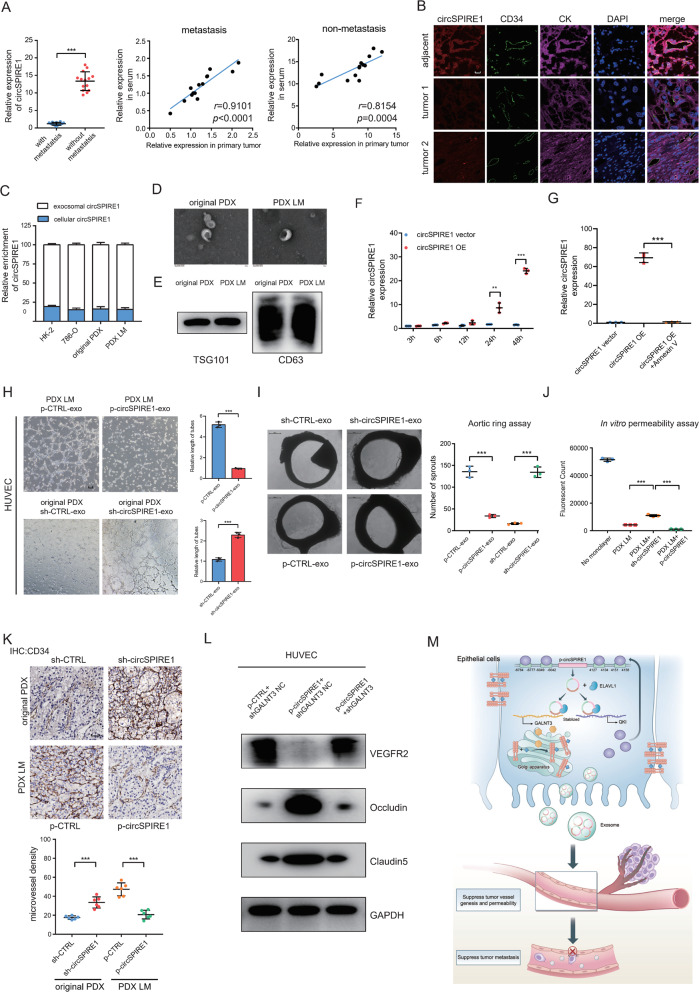
RCC-secreted circSPIRE1 is transferred to endothelial cells and suppressed vascular permeability and angiogenesis. **A** Left, qRT-PCR analysis of circSPIRE1 expression in sera of 14 RCC patients with metastasis and 14 patients without metastasis. Data represent mean ± S.D.; The *P* values were determined by Student’s *t* test. Right, correlation of circSPIRE1 expression between in serum and in primary tumor. **B** Correlation analysis of circSPIRE1 (red) expression in RCC epithelium and stroma (labeled by CK, purple) and their adjacent endothelial cells (labeled by CD34, green). CircSPIRE1 levels were determined by the FISH score, Spearman rank correlation coefficient (rs), and *P* value. *P* value is from Spearman’s test. Scale bar, 50 µm. **C** Cytoplasmic and exosome RNA fractionation experiment showing that circSPIRE1 mainly localized in the exosome. **D** Transmission electron microscopy of exosomes derived from original PDX and PDX LM. **E** Exosome marker TSG101 and CD63 analyzed through western blotting. **F** RT-PCR analysis of circSPIRE1 expression in HUVECs incubated with exosomes derived from p-CTRL and p-circSPIRE1 cells for 3, 6, 12, 24, and 48 h. Data represent mean ± S.D.; dot plots reflect data points from three independent experiments. The *P* values were determined by Student’s *t* test. **G** Effect of p-CTRL, p-circSPIRE1 and p-circSPIRE1 + Annexin V treatments on circSPIRE1 expression in HUVECs. Data represent mean ± S.D.; dot plots reflect data points from three independent experiments. The *P* values were determined by Student’s *t* test. **H** Effect of exosomes derived from circSPIRE1 knockdown and control as well as overexpression and control on tube formation ability of HUVECs by tube formation assay. Mean ± S.D. are provided (*n* = 3/group). Scale bar represents 100 µm. **I** Effect of exosomes derived from circSPIRE1 knockdown and control as well as overexpression and control on vascular outgrowth of rat aortic rings. Vascular outgrowth was quantified by counting all sprouts from one ring. Left, representative aortic ring images (*n* = 3/group). Data represent mean ± S.D.; dot plot reflects data points from three independent experiments. Scale bar represents 200 µm. **J** Permeability of the HUVEC monolayers to rhodamine–dextran (70 kDa) after exposure to exosomes derived from circSPIRE1 knockdown or overexpression cells for 72 h. Mean ± S.D. are shown. Dot plots reflect data points from three independent experiments. The *P* values were determined by Student’s *t* test. **K** Effect of circSPIRE1 knockdown or overexpression on tumor microvessel genesis. Top, representative tumor stained with CD34 anti-body. Bottom, microvessel density for each group. Data represent mean ± S.D.; dot plots reflect data points from six independent experiments. The *P* values were determined by Student’s *t* test. **L** VEGFR2, Occluding and Claudin5 protein expression in circSPIRE1 overexpression or circSPIRE1 overexpression combined *GALNT3* knockdown HUVECs by western blot. **M** Schematic diagram of mechanism of circSPIRE1 during RCC metastasis in tumor microenvironment.

We also collected serum samples from 14 patients with RCC with metastasis and 14 patients with RCC without metastasis. Interestingly, circSPIRE1 expression was significantly downregulated in sera derived from patients with metastasis compared to patients without metastasis, and the expression level of circSPIRE1 in RCC tissues and sera was significantly correlated (Fig. 7A). Furthermore, the evaluation of the histological FISH analysis showed that circSPIRE1 was expressed in both RCC cells and cancer-adjacent endothelial cells. In addition, the level of circSPIRE1 in RCC cells was positively related to that in cancer-adjacent endothelial cells (Fig. 7B, Table S9). A previous study reported that cells could communicate with neighboring cells through the important cell-to-cell mediator, exosomes, to render the primary microenvironment [32]. Thus, we asked whether circSPIRE1 could be secreted and transferred via exosomes between RCC cells and endothelial cells. To answer this question, we extracted cellular RNAs from the cells and exosomal RNAs from the conditioned media (CM) of RCC cell lines and detected that circSPIRE1 was enriched in exosomes. (Fig. 7C). Through transmission electron microscopy, we identified exosomes from original PDX and PDX LM CM as membrane-encapsulated particles with a range of 50–100 nm in size (Fig. 7D). Furthermore, the exosome markers CD63 and TSG101 were detected by western blotting (Fig. 7E). Next, we constructed siRNAs targeting the back-splice region of circSPIRE1 (circSPIRE1 si) or the circSPIRE1 overexpression plasmid (circSPIRE1 OE) to knockdown or overexpress circSPIRE1 in original PDX and PDX LM cells. We observed a marked upregulation or downregulation of exosomal and cellular circSPIRE1, respectively, but the level of mRNA levels remained statistically unchanged (Fig. S3F, G). Further, we incubated PKH67 labeled exosomes derived from with HUVEC and observed PKH67 transmission to HUVEC (Fig. S3H). Furthermore, we detected the levels of circSPIRE1 in HUVECs that were incubated with exosomes from control cells and cells with circSPIRE1 overexpression. CircSPIRE1 was markedly upregulated in HUVEC incubated with exosomes overexpressing circSPIRE1 (Fig. 7F). However, the HUVEC CM did not alter the expression level of circSPIRE1 in RCC cells after incubation for 48 h (Fig. S3I). Furthermore, the inhibitor of exosome internalization, Annexin V, abolished the circSPIRE1 upregulation effect of exosomes derived from circSPIRE1-overexpressed cells (Fig. 7G). These results suggested that cancer-secreted exosomes contained circSPIRE1, which could be transmitted to endothelial cells in vitro and in vivo.

### CircSPIRE1 suppressed vascular permeability and angiogenesis

Because the microvessel environment is also a key to initiating metastasis, we explored the impact that exosomal circSPIRE1 had on endothelial cells. We wondered if exosomes derived from cells overexpressed with circSPIRE1 could suppress vascular permeability and angiogenesis. Compared with controls, conditioned media from circSPIRE1 over-expressed cells led to shorter tubes in tube formation assay, less sprouts from one ring in aortic ring assay, and less fluorescence in the permeability assay. In contrast, the CM from the circSPIRE1 knockdown cells led to the opposite outcome (Fig. 7H–J). Next, we performed an immunohistochemistry assay using subcutaneous tumors acquired by injecting original PDX sh-CTRL, original PDX sh-circSPIRE1, PDX LM p-CTRL, and PDX LM p-circSPIRE1 subcutaneously. The *CD34* antibody was used to detect the density of tumor vessels. IHC showed that tumors developed from PDX LM p-circSPIRE1 had a lower density of tumor vessels compared to PDX LM p-CTRL, while tumors developed from original PDX sh-circSPIRE1 had a higher density of tumor vessels compared to original PDX sh-CTRL (Fig. 7K). Further western blotting was conducted and compared with controls, conditioned media from circSPIRE1 over-expressed cells led to decreased VEGFR2 and increased Occludin, and Claudin5. While *GALNT3* knockdown in HUVEC cells reversed the western blotting assays outcome (Fig. 7L). These data suggested that exosomal circSPIRE1 enhanced the integrity of endothelial barriers and suppressed angiogenesis. In addition, the low expression of circSPIRE1 might be a key factor associated with enhanced vascular permeability and angiogenesis, which ultimately led to tumor metastasis (Fig. 7M).

### Chitosan-EGCG nanoparticles delivered circSPIRE1 overexpression plasmid in vitro with low cytotoxicity and high transfection efficiency

**Fig. 8:**
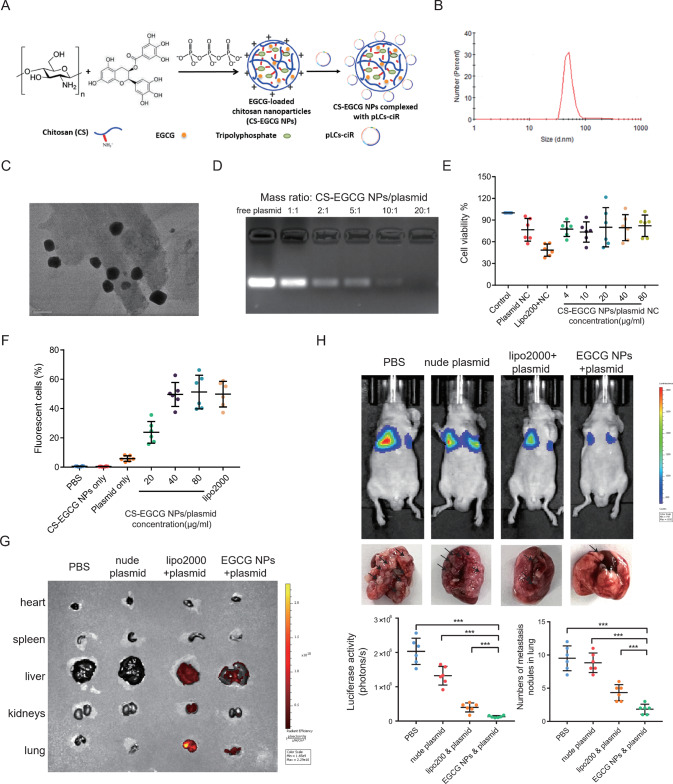
CS-EGCG NPs/circSPIRE1 overexpression plasmid nanocomplexes inhibits RCC metastasis in vivo. **A** Chemical structure of Chitosan-EGCG nanoparticles and schematic structures of CS-EGCG NPs complexing with plasmid. **B** Mean particle size of CS-EGCG nanoparticles. **C** TEM image of CS-EGCG NPs. **D** Binding ability of CS-EGCG NPs to circSPIRE1 overexpression plasmid at different mass ratios characterized by gel electrophoresis. **E** Cell cytotoxicity of CS-EGCG NPs complexing with plasmid assessed by CCK8 assay and the percentage of viable cells was calculated relative to untreated control cells. Data represent mean ± S.D.; dot plots reflect data points from six independent experiments. **F** Percentage of fluorescent cells analyzed by flow cytometry. Data represent mean ± S.D.; dot plots reflect data points from six independent experiments. **G** Fluorescence images of nude mice bearing metastatic renal cell carcinoma after tail vein injection of PBS, nude plasmid, lipo2000 and plasmid, as well as CS-EGCG NPs and overexpression plasmid complexes. **H** Top, bioluminescent in the lungs and tumor metastasis formed in the lungs after tail vein injection of PBS, nude plasmid, lipo2000, and plasmid, as well as CS-EGCG NPs and overexpression plasmid complexes. Bottom, statistical analysis of bioluminescent and tumor metastasis plots (*n* = 6 mice/group). Data represent mean ± S.D.; dot plot reflects data points from independent experiments. The *P* values were determined by Student’s *t* test.

Studies have suggested that the combination of the bioactive anticancer component and the gene would not only improve the transfection efficiency of the gene but also possess a synergistic effect of anticancer agents and gene therapies [33]. Therefore, in this study we applied effective and safe cross-linked chitosan-EGCG nanoparticles (CS-EGCG NPs) for the construction and delivery of the circSPIRE1 overexpression plasmid to cells. EGCG is one of the most abundant and active constituents of polyphenolic green tea, which has been shown to possess extensive therapeutic properties, including anti-inflammatory, anti-diabetic, anti-obesity, and anti-cancer effects [34–38]. Chitosan is a natural cationic biocopolymer with good biocompatible and biodegradable properties. Under slightly acidic conditions, chitosan could be modified to interact with genes through ionic interactions and can be delivered to cells [39, 40]. The preparation scheme of cross-linked CS-EGCG nanoparticles and their complexing with plasmid is presented in Fig. 8A. The mean particle size of the CS-EGCG nanoparticles was 92 ± 19 nm (Fig. 8B). This particle size should allow them to enter cancer cells through cell endocytosis. The average zeta potential of the CS-EGCG nanoparticles was 28.2 ± 2.6 mV under near neutral pH conditions. The positive charge of the CS-EGCG nanoparticles would allow them to bind to negatively charged ions and benefit the controlled release of pLCs-ciR. We used transmission electron microscopy (TEM) to characterize the CS-EGCG NPs (Fig. 8C).

CS-EGCG NPs and plasmid complexes were also tested for their ability to bind RNA using agarose gel electrophoresis. Compared to the free plasmid, when the mass ratio of CS-EGCG NPs and the plasmid was 20:1 or higher, the plasmid band was almost invisible, suggesting a high binding ability of the CS-EGCG NPs (Fig. 8D). CCK8 assays with HK-2 cells showed that CS-EGCG NP and negative control plasmid complexes had no apparent cytotoxic effect at 80 μg/mL, a concentration that was two-fold higher than that required for high transfection efficiency (Fig. 8E). Next, we analyzed the cellular uptake of CS-EGCG NPs and of the overexpression plasmid in PDX LM cells under an inverted fluorescence microscope. When the total concentration of CS-EGCG NP and plasmid reached 40 μg/mL, cells incubated with complexes exhibited almost similar transfection efficiency compared to transfection of plasmids using liposomal-based transfection using Lipofectamine 2000 (lipo2000). When the dose of complexes was up to 80 μg/mL, there was only a modest change in transfection efficiency (Fig. 8F). Thus, we concluded that the optimal dose of CS-EGCG NPs and the overexpression plasmid concentration was 40 μg/mL. In conclusion, the low cytotoxicity and high transfection efficiency of CS-EGCG NPs suggested that they were a promising nanocarrier construct of an anticancer plasmid.

### Nanomedicine consisting of CS-EGCG NPs and circSPIRE1 overexpression plasmid suppressed metastasis in vivo

To test whether CS-EGCG NPs and circSPIRE1 overexpression plasmid have clinical significance, we evaluated the nanomedicines in a metastatic RCC model obtained via tail vein injection of PDX LM cells. Three weeks after injection, we divided 24 nude mice equally into four groups receiving different treatments. Mice were injected through the tail vein with phosphate-buffered saline (PBS), nude plasmid, lipo2000, and plasmid, as well as CS-EGCG NPs and overexpression plasmid complexes. A total of 4 μg plasmid DNA were injected per mouse and at a mass ratio of CS-EGCG NPs/circSPIRE1 of 20:1. Two injections were performed per week for an additional 3 weeks. The biodistribution of the complexes in mice was detected by the Xenogen IVIS Lumina system after the mice were euthanized. CS-EGCG NPs and circSPIRE1 showed complex accumulation mainly in lung metastasis, compared to the lipo2000 and plasmid group treated groups, which accumulated in the liver, kidneys, and lung metastasis (Fig. 8G). The liver and kidneys proved to be non-metastases sites based on hematoxylin-eosin (HE) staining of tissue slices. Only the background fluorescence in metastasis could be observed in the plasmid-only group (Fig. 8G). These results suggested that the nude plasmid could be quickly cleared by the bloodstream in vivo in the absence of nanoparticle coats and that the CS-EGCG NPs was stable in the circulation and could be specifically delivered to metastasis sites in comparison to lipo2000. Subsequently, we determined that lung metastasis was significantly suppressed following treatment with CS-EGCG NPs as the plasmid carrier compared to the lipo2000 carrier (Fig. 8H). These results suggested that CS-EGCG NP and circSPIRE1 overexpression plasmid complexes were able to specifically target metastasis and suppress metastasis in vivo.

## Discussion

The characteristics of circRNAs, including stability and high abundance, have attracted great interest in cancer research. Furthermore, the role of circRNAs in RCC is becoming better understood [17]. Nonetheless, considering the poor 5-year cancer-specific survival rates of patients with advanced RCC, the relationship between metastasis and circRNAs still requires thorough investigation [41]. In addition, subtyping of cells with different metastasis potential within a tumor classified by a circRNA expression profile is poorly defined. In the present study, considering the heterogeneity of the original clinical sample, we simulated metastasis in the PDX model and screened for a specific clone that easily detaches from the primary tumor to initiate metastasis. CircSPIRE1, which predicted a better clinical outcome, was identified by high-throughput sequencing and confirmed to be downregulated in patients with RCC with metastasis. Specifically, circSPIRE1 increased the stability of downstream mRNA by forming a ternary complex of circSPIRE1-RNA-protein that resulted in an enhanced epithelial state and self-renewal of circSPIRE1. Furthermore, we revealed that circSPIRE1 could be packed and secreted in exosomes to modify the tumor microenvironment. The strength of our study was that our screening assay for clones with high metastatic potential was carried out in vivo starting from a fresh clinical sample. Compared to previously applied screening approaches based on Transwell assays using homogeneous cell lines, in vivo assays, using clinical samples that are intrinsically heterogeneous, may better reproduce the complexity of metastatic RCC in humans.

In recent decades, circRNAs have been reported to perform biological functions through several well-studied mechanisms. For circRNAs which are predominantly localized in the cytoplasm, most circRNAs have been proposed to act as miRNA sponges and interact with RBP to act as protein sponges. Although most circRNAs are non-coding, some circRNAs undergo translation under specific conditions [13]. However, in the urinary system, contrasting opinions have arisen on the role of circRNAs as miRNA sponges, due to evidence indicating that identical circRNAs may display opposite roles [42]. Moreover, a potent mechanism whereby circNSUN2 can stabilize *HMGA2* mRNA by forming an RNA-protein ternary complex has been raised in colorectal carcinoma [43]. The model demonstrated that circRNAs can serve as a guide to promote targeted binding of RBPs to downstream RNAs. Our MS analysis results revealed that ELAVL1, a conventional RBP, was greatly enriched in the pull-down complex, and motivated us to explore the guiding effect of circRNAs in RCC for the first time.

ELAVL1 is capable of regulating the half-life of mRNAs by binding AU-rich elements and increasing their expression [44]. Several *ELAVL1* target genes are involved in tumorigenesis and proliferation, such as hypoxia-inducible factor 1α (*HIF-1α*), vascular endothelial growth factor (*VEGF*), cyclooxygenase-2 (*COX-2*), and *β-catenin* [45–47]. ELAVL1 contains three RRM motifs, whose 3D structures have been characterized. RRM1 is the primary ARE-binding domain in *ELAVL1*, while RRM2 greatly improves the RNA-binding affinity of ELAVL1 [29]. Further, it is reported that RRM3 counteracts the stabilization effect of ARE-containing RNAs [24]. In our study, we used the minimum free energy algorithm to predict the 3D model of circSPIRE1. Combining the 3D model of circSPIRE1 and the crystal structure of RRMs, we applied in silico simulation models including docking, scoring, clustering, and refinement to define the most promising models. Subsequently, through molecular experiments, we determined that ELAVL1 protein could bind to downstream RNA through RRM1 and RRM2. In addition, circSPIRE1 bound to RRM3, instead of RRM1 and RRM2, which would have resulted in a competition with mRNA. Furthermore, circSPIRE1 binding to RRM3 interfered with the potential binding of mRNA and neutralized the destabilization effect of RRM3. The reverse complementary sequences in circSPIRE1 and targeted downstream mRNA ensured the specificity of the stabilization effect.

CircRNAs have been validated to be tissue-specific, cell-specific, and abundant in eukaryotes, and several mechanisms such as back-splicing and exon-skipping have been proposed to explain the biogenesis of circRNAs [13]. RBPs including QKI could bind to specific motifs in the flanking introns and bring the downstream splice donor site and the upstream splice acceptor site into close proximity to achieve back-splicing [48]. This study first suggests that circRNAs could achieve self-renewal in RCC through a positive feedback loop, in which circSPIRE1 stabilizes *QKI* transcription and elevated *QKI* expression, in turn, supports the expression of circSPIRE1. However, the feedback loop may be interrupted due to rapid replication of cancer cells that dilute circRNA expression and ultimately leads to favor cancer progression [49].

The glycosylation of E-cadherin has been reported to influence the adhesive function of this adhesion molecule [50]. Among the various glycoforms of cadherins, O-Glycosylation has drawn greater attention due to its important role in carcinogenesis [51]. *GALNT3* is a member of a large family of homologous genes called O-GalNAc glycosyltransferases (GALNTs), which initiates O-GalNAc glycosylation in the Golgi apparatus by transfer of N-GalNAc to serine and threonine residues [52]. Reduced expression of *GALNT3* was previously reported in mesenchymal trophoblast stem cells and breast cancer cells, suggesting that *GALNT3* may be important in promoting an epithelial phenotype [12]. In contrast, GlcNAcylation was previously considered to trigger the migration/invasion of breast cancer cells by decreasing cell surface E-cadherin levels [53]. However, lentiviral-mediated O-linked-β-N-acetylglucosamine transferase (OGT) silencing was used to reduce the degree of global GlcNAcylation. The findings suggested that the GlcNAcylation of p120 and β-catenin but not of E-cadherin itself might play a role in the decrease of cell surface E-cadherin localization. In our present study, circSPIRE1 exerted its activity primarily by stabilizing GALNT3, a member of a large evolutionarily conserved family of GALNTs, and was not likely able to alter the global level of GlcNAcylation. Changes in *GALNT3* expression in RCC cells did not influence the degree of GlcNAcylation of p120 and β-catenin, which suggested that in RCC, GALNT3 targets E-cadherin instead of p120 and β-catenin and O-GlcNAcylation of E-cadherin maintains the epithelial state.

Indeed, due to functional redundancies and compensatory mechanisms of GALNTs, the E-cadherin glycosylation network warrants further investigation. The specific pathway through which circSPIRE1 influences other EMT markers and tumor angiogenesis will be the aim of our future studies. Another limitation is that it would be more reliable if circSPIRE1 is also validated in another paired PDXs.

In summary, we provide primary evidence that circSPIRE1 is a specific metastasis suppressor in RCC. The tumor suppressing effect was confirmed in vivo. CircSPIRE1 exerts its activity by forming a ternary complex to stabilize *GALNT3* and *QKI* mRNA. More importantly, CS-EGCG-containing NPs and the circSPIRE1 overexpression plasmid were able to reverse the high metastatic potential of PDX LM. Our findings provide a foundation for developing diagnostic markers or potential therapeutic intervention in metastatic RCC.

### Study approval

Clinical samples used in this study were approved by the Medical Ethics Committee of the Sun Yat-sen University Cancer Center and the First Affiliated Hospital of Sun Yat-sen University and written informed consent was received prior to participation. All animal experiments were approved and performed in strict accordance with the guidelines of the Institutional Animal Care and Use Committee of SYSUCC.

## Materials and methods

### Care and maintenance of animals

All in vivo experiments were approved and performed in strict accordance with the guidelines of the Institutional Animal Care and Use Committee of SYSUCC. BALB/c nude mice (3–4 weeks old) were purchased from Vital River Laboratory Animal Technology (China). Male mice 4–5 weeks old NCG (NOD/ShiLtJGpt-Prkdcem26Cd52Il2rgem26Cd22/Gpt) (GemPharmatech, China) were housed in a sterile, pathogen-free facility. To generate the metastatic model, a set of 4–5 weeks old male NCG mice were transplanted with fragments of fresh human RCC tissues into the flanks subcutaneously (*n* = 6). Till the subcutaneous tumors were formed, we transplanted the tumor pieces into subrenal capsule of another set of NCG mice. Lung metastasis developed were collected and re-implanted orthotopically in the subrenal capsule to select a pure metastatic population. After repeated formation and passage of tumors, we successfully found a metastatic clone with a high incidence (>90%) of spontaneous lung metastases at every passage. For the orthotopic metastasis experiment, a sub-capsular injection was conducted with cells stably transfected with negative control, shRNA, or circSPIRE1 overexpression plasmid, 1 × 10^5^ cells/5 μL per mouse. Each group contains six mice which were randomly allocated. All mice were sacrificed after 8 weeks. Images were taken using a Xenogen IVIS Lumina system. Lungs were collected for HE staining and pulmonary metastasis loci were counted in each sample. To conduct CS-EGCG NPs and circSPIRE1 overexpression plasmid treatment assay, a metastatic RCC model was obtained via tail vein injection of PDX LM cells. Three weeks after injection, 24 nude mice were randomly allocated into four groups receiving phosphate-buffered saline (PBS), nude plasmid, lipo2000, and plasmid, as well as CS-EGCG NPs and overexpression plasmid complexes through tail vein injection. A total of 4 μg plasmid DNA were injected per mouse and at a mass ratio of CS-EGCG NPs/circSPIRE1 of 20:1. Two injections were performed per week for an additional 3 weeks. The biodistribution of the complexes in mice was detected by the Xenogen IVIS Lumina system after the mice were euthanized.

No sample size calculations were performed. Sample size was determined according to our experience as well as literature reporting in terms of specific experiment. Randomization method was used to determine how animals were allocated to different groups. To achieve randomization, all animals were numbered by body weight, then, random number table was used to allocate animals to experimental groups. During the study, no data was excluded from the experiments, and no blinding was done.

### RCC patient samples

For circRNA expression profiling, we obtained 142 paired pathologically diagnosed RCC tumor samples from the Sun Yat-sen University Cancer Center (SYSUCC). The collection of samples was carried out consecutively between 2007 and 2018. Corresponding adjacent noncancerous tissues were acquired approximately ≥5 mm away from the tumor site. The clinical features of the RCC patients are summarized in Table S1. Regular follow-up was carried out and DFS was determined from the date of surgery to the date of the first evidence of clinical recurrence. Five no clinically metastasis cases and five metastasis cases were obtained from the First Affiliated Hospital of Sun Yat-sen University. Fourteen RCC patients with metastasis and fourteen RCC patients without metastasis were enrolled from the Sun Yat-sen University Cancer Center and the First Affiliated Hospital of Sun Yat-sen University. Samples used in this study were approved by the Medical Ethics Committee of the Sun Yat-sen University Cancer Center and the First Affiliated Hospital of Sun Yat-sen University. The informed consent of each patient was obtained.

### Cell lines and cell culture

Human RCC cell lines (786-O, ACHN, 769-P), a human renal proximal tubular epithelial cell line (HK-2), a human embryonic kidney cell line (HEK-293T), and human umbilical vein endothelial cells (HUVEC) were purchased from the Cell Bank of the Chinese Academy of Sciences. 786-O 769-P, and PDX originated cell lines (original PDX and PDX LM) were cultured in RPMI 1640 (Gibco, China). HK-2, and HEK-293T were cultured in DMEM (Gibco, China). HUVECs were cultured in F12-K medium (Gibco, China). All media were supplemented with 10% FBS (PAN-Seratech, Germany) and 1% penicillin-streptomycin (Biosharp, China). The cells were cultured in a humidified incubator with 5% CO_2_ at 37 °C. All cell lines were authenticated by the short tandem repeat DNA profiling test and tested negative for mycoplasma contamination. All the cell lines were not cultured for more than 2 months.

### Bioinformatics analysis

The Cancer Genome Atlas (TCGA) RCC sequencing data, clinical and survival data were obtained from Firebrowse (http://firebrowse.org/). R (version 3.4.3) (https://www.r-project.org/) was used for subsequent data analysis. Mfold (version2.3) was used to determine the secondary structures of circSPIRE1 [21]. RNA Composer was used to generate the 3D structure [22, 23]. NPDock web server was used to conduct the docking procedure [24]. The 3D crystal structure of the RRM3 domain was derived from the Protein Data Bank [25]. Discover studio was used to visualize the docking model and obtain the contact distance between atoms. MEME SUITE was applied to discover the QKI binding motif in intron 3 and intron 6 flanking circSPIRE1 [32].

### RNA interference (RNAi) and transfection

For transient transfection of circSPIRE1, *GALNT3* and *QKI*, two different siRNAs and one scrambled siRNA served as negative control were synthesized by RiboBio (China). siRNAs were transfected into RCC cells using RNAiMAX (Invitrogen, USA) according to manufacturers’ instructions. Function tests were performed 48 h after transfection. The knockdown lentiviruses of circSPIRE1, *GALNT3* and *QKI* as well as corresponding negative controls were provided and examined by Genechem (China). The target sequences for constructing lentiviral shRNAs and siRNAs are listed in Supplementary Data 4.

### Plasmid construction

For the circSPIRE1 over-expression plasmids, circSPIRE1 cDNA was synthesized and cloned into a pLC5-ciR vector (Geneseed, China). An empty vector served as negative control. Flag-ELAVL1 WT and truncated plasmids, truncated circSPIRE1, circSPIRE1-mRNA (*GALNT3* and *QKI*) binding mutation plasmid and truncated *QKI* binding mutation plasmid were obtained from Genecreate (China). Plasmids were transfected using Lipofectamin 3000 (Invitrogen, USA), according to the manufacturer’s instructions. In vivo bioluminescence imaging was conducted following injection of cells transfected with vectors containing Gaussia luciferase (Gluc).

### RNA quantitative real-time polymerase chain reaction

Total RNA was extracted with TRIzol reagent (Invitrogen, USA) and measured by NanoDrop 2000 (Thermo Fisher Scientific, USA). PrimeScript RT Master Mix (Takara, China) was used in RNA reverse transcription. Real-time polymerase chain reaction (RT-PCR) was carried out using SYBR Green SuperMix (Roche, Basel, Switzerland) in the LightCycler 480 (Roche, Basel, Switzerland). Primer sequences are provided in Supplementary Data 4. The results of circRNA and mRNA levels were calculated in a 2^−ΔΔCt^ method and normalized to *GAPDH* in cytoplasm, exogenous λ polyA (Takara, China) in exosomes.

### Western blot

Cell lysis buffer containing proteinase inhibitor (Beyotime, China) was used for cells in culture dishes. The lysates were then incubated on ice for 15 min before a 15-min centrifugation (12,000 × *g*, 4 °C). The supernatants were collected and the protein concentration was measured with a BCA protein assay kit (ThermoFisher, USA). Equal protein samples were loaded into each lane of 10% SDS-PAGE gels. Subsequently, proteins were transferred to PVDF membranes, which were blocked with nonfat milk at room temperature for 1 h. The membranes were then incubated overnight at 4 °C with primary antibody and subsequently with secondary antibody for 1 h at room temperature. Hybridizations were detected using high-signal ECL Western Blotting Substrate (Tanon, China) on a ChemiDoc Touch Imaging System (BIO-RAD, USA). The antibodies used in western blots were as follows: TSG101 (1:1000 dilution, ab125011, Abcam, USA), CD63 (1:1000 dilution, ab252919, Abcam), ELAVL1 (1:1000 dilution, ab200342, Abcam), GAPDH (1:1000 dilution, 5174, Cell signaling, USA), anti-Flag for western blot (1:1000 dilutions, 8146, Cell Signaling), GALNT3 (1:1000 dilution, AP51768PU-N, Origene, USA), QKI (1:1000 dilution, A300-183A, BETHYL, USA), ZNF596 (1:1000 dilution, TA316199, Origene), E-cadherin (1:1000 dilution, ab40772, Abcam), N- cadherin (1:5000 dilution, ab76011, Abcam), Vimentin (1:1000 dilution, 5741, Cell Signaling), HRP-conjugated goat anti-mouse (1:5000 dilution, Proteintech, China), and HRP-conjugated goat anti-rabbit antibody (15,000 dilution, Proteintech).

### Actinomycin D and RNase R treatment

When at 60% confluency in six-well plates, cells were treated with 5 μg/mL actinomycin D or DMSO and harvested at specific time points. Total RNA was incubated with 3 U/μg of RNase R (Epicentre Technologies, USA) for 15 min at 37 °C. Afterwards, expression levels of circSPIRE1 and mRNAs were quantified through qRT-PCR.

### Northern blotting

The labeled RNA probes were synthesized from GenePharma (China). Northern blotting was performed using NorthernMax Kit from Ambion (Life Technologies, USA) according to manufacturer’s instructions. Total RNA with or without RNase R digestion was loaded on different lanes of a 2% agarose gel and transferred to a Hybond-N^+^ membrane (GE Healthcare, Sweden) by capillary transfer. Hybridization temperature was 58 °C and overnight incubation with biotin-labeled oligonucleotide probe was conducted. The Chemiluminescent Nucleic Acid Detection Module (Thermo Fisher Scientific, USA) was used to detect the biotin-labeled RNA probes and *GAPDH* was used as an internal control. The probe sequences are shown in Supplementary data 4.

### Nuclear and cytoplasmic extraction

The PARIS Kit (Invitrogen) was used to separate nuclear and cytoplasmic lysates according to manufacturer’s instructions. Briefly, cells were lysed in Cell Fraction Buffer on ice for 10 min. After centrifugation at 500 × g for 3 min at 4 °C, the supernatant was collected as a cytoplasmic fraction. The nuclei were collected followed by washing the pellet with Cell Fraction Buffer.

### Exosome isolation, characterization, and treatment

RCC cells were cultured in medium with 10% exosome-free medium (System Biosciences) for 72 h before the isolation of exosomes. The supernatant was collected and centrifuged at 300 × *g* for 10 min, 2000 × *g* for 10 min, 10,000 × g for 30 min, in turn. After filtration with a 0.22-pm filter (Millipore, USA), the filtrate was centrifuged at 120,000 × *g* for 70 min twice (Beckman Coulter, USA). The pellets were resuspended with PBS and prepared for the subsequent experiments. For transmission electron microscopy, exosomes were fixed with 2% paraformaldehyde and placed on 200-mesh Formvar-coated grids. The grids were then stained using 2% phosphotungstic acid for 2 min and observed on a transmission electron microscope (Hitachi H-7500). The size of exosomes was detected by Nanosight ns300 (Malvern Instruments Ltd., UK). For exosomes labeling, exosomes were labeled using PKH67 membrane dye (Sigma). Labeled exosomes were collected by ultracentrifugation after washing in 10 mL PBS, and resuspended in PBS. For cell treatment, exosomes were incubated with recipient cells for 48 h. An ExoQuick ULTRA EV Isolation Kit for Serum (System Biosciences) was used to isolate the exosomes from the serum of RCC patients. The RNAs in exosomes were extracted using an Exosome RNA Purification Kit (EZBioscience, USA).

### Fluorescence in situ hybridization and immunohistochemistry

Oligonucleotide-modified probe sequence for circSPIRE1, *GALNT3* mRNA, and *QKI* mRNA was synthesized from GenePharma (China). Paraffin-embedded tissue blocks were cut into 2.5-μm sections and transferred to glass slides. FISH was performed in tissue sections using a fluorescence in situ hybridization (FISH) kit purchased from GenePharma (China) following the manufacturer’s protocol. The images were obtained using the OLYMPUS FV1000 confocal microscope (Japan). Fluorescence intensity was analyzed by ImageJ. Pearson’s correlation coefficient was analyzed by OLYMPUS FV1000 software. The probe sequences are shown in Supplementary data 4. For immunohistochemistry (IHC), deparaffinization, rehydration, antigen retrieval, and staining were conducted in order. Normal goat serum blocking buffer (no. ZLI-9056), Rabbit two-step kit (no. PV-6001), and DAB kit (no. ZLI-9017) were purchased from ZSGB-BIO (China). The following primary antibodies were used: CD34 (1:200 dilution, ab81289, Abcam), CK (1:200 dilution, ab191208, Abcam), ELAVL1 (1:250 dilution, ab200342, Abcam), GALNT3 (1:100 dilution, AP51768PU-N, Origene), QKI (1:200 dilution, A300-183A, BETHYL), E-cadherin (1:250 dilution, ab40772, Abcam), and anti-Giantin (1:100 dilution, ab37266, Abcam).

### Immunofluorescence

Cells grown on a confocal dish (Corning, USA) were fixed with 4% paraformaldehyde in PBS for 15 min on ice and then permeabilized with 0.1% TritonX-100 for 10 min. Then, cells were washed twice with PBS and blocked with 5% BSA for 30 min at 37 °C. Afterwards, cells were incubated with primary antibody overnight at 4 °C and washed with PBS. The secondary antibody was incubated for 30 min at 37 °C, followed by staining with DAPI for nucleus staining. Fluorescent images were taken with OLYMPUS FV1000 confocal microscopy. Fluorescence densities were analyzed by ImageJ. Correlation analysis was carried out with GraphPad Prism 7 software.

### Transwell assays

Cells were starved for 8 h in serum-free medium. Then, cells were collected, resuspended in serum-free medium, and added to Transwell inserts (Corning, USA). Medium containing 10% FBS was added to the bottom chamber. After incubation for 6–12 h, the migrated cells in lower filters were fixed with 4% polyformaldehyde (Beyotime, China), and stained with 0.4% crystal violet (Beyotime, China) for 20 min. Migrated cells were calculated by capturing five random fields under an Olympus IX83 inverted microscope (Japan). All experiments were performed in triplicate.

### Tube formation assay, aortic ring assay, and in vitro permeability assay

For the tube formation assay, Matrigel matrix (Corning) was plated on a 24-well plate and incubated at 37 °C for 30 min to allow the matrigel to polymerize. Treated HUVECs were seeded on the matrigel-coated well and incubated at 37 °C in 5% CO2 humidified atmosphere. After 12 h, tube formation was observed with a microscope. The tube formation ability was determined by measuring the number of tubes. For the aortic ring assay, 8–12-week-old Sprague Dawley (SD) rats were sacrificed and thoracic aortas were cut into 1-mm cross-sections. Aortic sections were placed in wells pre-coated with matrigel for 30 min, 37 °C. F12-K medium (GIBCO, USA) was supplemented with 10% fetal bovine serum, 50 U/mL penicillin, 50 μg/mL streptomycin, 10 ng/mL vascular endothelial growth factor, and 25 μg/mL heparin for the first 24 h of incubation in vitro. Then the aortic rings were incubated with CM obtained from treated cells. Vascular outgrowth was quantified by counting all sprouts from one ring using ImageJ. For the in vitro permeability assay, HUVECs (10^5^ cells per well) were treated with CM from differently treated cells and seeded on Transwell filters (0.4-μm pore size; BD Biosciences) for 3 days. Rhodamine B isothiocyanate-dextran (average MW ~70,000; Sigma) was added to the top well of the Transwell filters for 30 min. Fluorescence of medium in the bottom well was quantified at 544 nm excitation and 590 nm emission. All assays were performed in triplicate and each experiment was repeated three times.

### RNA pull-down assays

The biotin-coupled RNA complex was pulled down by incubating the cell lysates with streptavidin-coated magnetic beads (Invitrogen, Carlsbad, USA). The RNA pull-down assay was performed using Pierce Magnetic RNA-Protein Pull-Down Kit (Thermo Scientific, USA) following the manufacturer’s instructions. The enrichment of circSPIRE1, *GALNT3*, or *QKI* in the capture fractions was evaluated by qRT-PCR analysis. The bound proteins were eluted from the packed beads and analyzed by SDS-PAGE. The proteins in the capture complex were identified by western blotting or silver staining or mass spectrometry analysis.

### Silver staining and mass spectrometry analysis

Silver staining was performed with a Fast Silver Stain Kit (Beyotime, China). Targeted region of gel was cut off and saved in microcentrifuge tubes. MS analysis was provided by BGI (Shenzhen, China). A protein presented exclusively in circSPIRE1 compared to controls, with unique peptides > 2 was considered significant.

### Immunoprecipitation of RNA-binding proteins

RIP was carried out using an EZ-Magna RIP Kit (Millipore, USA) according to the manufacturer’s instructions. The bounded proteins were further confirmed by western blotting. The immunoprecipitated RNA was subjected to qRT-PCR analysis.

### Docking simulations, contact maps, and identification of binding site residues

NPDOCK Server was used to determine the possible interaction of circSPIRE1 with ELAVL1. NPDOCK Server combines GRAMM for global macromolecular docking, scoring with a statistical potential, clustering, followed by refinement of best scored docked complexes from the three largest clusters. A distance-based approach was used to identify the binding site residues/nucleotides for protein-RNA complexes using a specific cutoff value. Two atoms (one in RNA and another in protein) were considered to interact with each other if the distance between them was < 3.5 Å determined by Discovery Studio.

### Electrophoretic mobility shift assay

Biotin-labeled RNA oligonucleotides were obtained from Umine Biotechnology (Guangzhou). The RNA Electrophoretic mobility shift assay (EMSA) assay was carried out using LightShift™ Chemiluminescent RNA EMSA Kit (Thermo Fisher Scientific, USA), according to the manufacturer’s instructions. Purified proteins were purchased from Origene (USA). For supershift assays, recombinant proteins were pre-incubated with antibodies at 0 °C for 20 min followed by the addition of the labeled probe. The probe sequences are shown in Supplementary data 4.

### Luciferase reporter assay

The *GALNT3* or *QKI* sequence was cloned separately downstream of the pmirGLO dual luciferase vector (Genecreate, China). Mutations were inserted at the binding sites. Cells were seeded in 24-well plates and transfected with a mixture of pmirGLO dual-luciferase reporter and siRNA at the same time. After 48 h, the relative luciferase activity was measured as the ratio between Firefly and Renilla luciferase activities with a Dual-luciferase Reporter Assay System (Promega, USA). Renilla luciferase activity was used as an internal control. The relative Luc/Rluc ratio was further normalized to that of the control sample in the circSPIRE1 knockdown or overexpression group.

### Lectin pull-down assay and immunoprecipitation

Protein lysates from treated cell lines were incubated with biotinylated VVL at 4 °C with gentle rotation for 16 h. Proteins bound to these biotinylated lectins were then pulled down using streptavidin-coated magnetic beads (Invitrogen, Carlsbad, USA). Pulled down proteins were then immunoblotted with anti-E-cadherin antibody (Abcam).

### Preparation of chitosan-EGCG nanoparticles

To prepare cross-linked chitosan-EGCG nanoparticles (CS-EGCG NPs), chitosan (1.5 g) was dissolved in 1% (v/v) aqueous acetic acid at a concentration of 1.5% (m/v) to form a transparent CS solution and the pH was adjusted to 5.0 using a 1 M NaOH solution. EGCG (0.02 g) was added to the CS solution under stirring until it was well dissolved. Subsequently, 10 mL of 3 mg/mL sodium tripolyphosphate (STPP) solution was added dropwise to the mixed solution under stirring at room temperature. After continuous stirring for 30 min, cross-linked CS-EGCG particle suspension was homogenized using high-speed homogenization at 10,000 r/min for 1 min. The suspension obtained was ultrasonicated for 5 s with 5 s intervals using a probe sonicator under 300 W for a total of 5 min to obtain fine CS-EGCG nanoparticles. After settling at room temperature for 2 h, the CS-EGCG nanoparticle suspension was collected after being dialyzed against pH 5.5 acetic buffer to remove unreacted STPP and free EGCG.

### Size and zeta potential characterization

The size and zeta potential of CS-EGCG nanoparticles were measured using a NanoZS Nanosizer (Malvern Instruments Ltd., UK) by photon correlation spectroscopy and electrophoretic mobility, respectively. All measurements were made in triplicate.

### Binding assay

CS-EGCG NPs and plasmid were mixed in a mass ratio of 1:1, 2:1, 5:1, 10:1, 20:1. The supernatants containing C-SLN-ASO complexes were loaded onto a 1.5% agarose gel, which was later visualized by the Gel Doc XR + system and analyzed with Image Lab software (BIO-RAD, USA).

### Statistical analysis

Statistical analyses were performed using GraphPad Prism 7 software and R (version 3.4.3) (https://www.rproject.org/). All experiments were carried out at least three times, and data from one representative experiment are shown. Data are presented as mean ± standard deviation (S.D.). Differences among/between sample groups were analyzed by one-way analysis of variance (ANOVA) or independent samples two-tailed student’s *t* test. Disease-free survival analysis was performed using Kaplan–Meier curves, with application of the Log-rank test to calculate statistical significance. The association between clinicopathological parameters and the expression level of circSPIRE1 was analyzed using chi-square test. We calculated the coefficient of variation and comparisons between the groups were performed. Univariate and multivariate hazard ratios (HRs) were calculated using Cox regression. The statistical difference was confirmed when the *P* value < 0.05 (*P < 0.05; **P < 0.01; ***P < 0.001).

## Supplementary information

supplementary materials

Data S1

Data S2

Data S3

Data S4

Data S5

supplementary table 5

supplementary table 6

## Data Availability

RNA-SEQ data and the mass spectrometry data was uploaded to Figshare database (Figshare 10.6084/m9.figshare.20293743). Figure S2E were generated from publicly available databases from TCGA. The human sequence data generated in this study are available upon reasonable request from the corresponding author. Other data generated in this study are available within the article and its supplementary data files.
