# Supplementary material for: Exosomal circSPIRE1 mediates glycosylation of E-cadherin to suppress metastasis of renal cell carcinoma
Source: Oncogene. 2023 Apr 12;42(22):1802–20. doi: 10.1038/s41388-023-02678-7 (PMC10238271; doi:10.1038/s41388-023-02678-7)
Supplement: Supplementary file 1 — supplementary materials [file 41388_2023_2678_MOESM1_ESM.docx]

Supplementary Materials for

**Exosomal circSPIRE1 mediates glycosylation of E-cadherin to suppress metastasis of renal cell carcinoma**

Guannan Shu, Xuanxuan Lu, Yihui Pan, Junjie Cen, Kangbo Huang, Mi Zhou, Jun Lu, Jiaqi Dong, Hui Han, Wei Chen, Juan Lin*, Junhang Luo*, Jiaxing Zhang*

*Corresponding author. Email: zhangjx25@mail.sysu.edu.cn (J.Z.); luojunh@mail.sysu.edu.cn

(Junhang L.); linj89@mail.sysu.edu.cn (J.L.)

**This PDF file includes:**

Figs. S1 to S5

Tables S1 to S9

**Other Supplementary Materials for this manuscript include the following:**

Data S1 to S5

Supplementary Figures

**Figure S1**


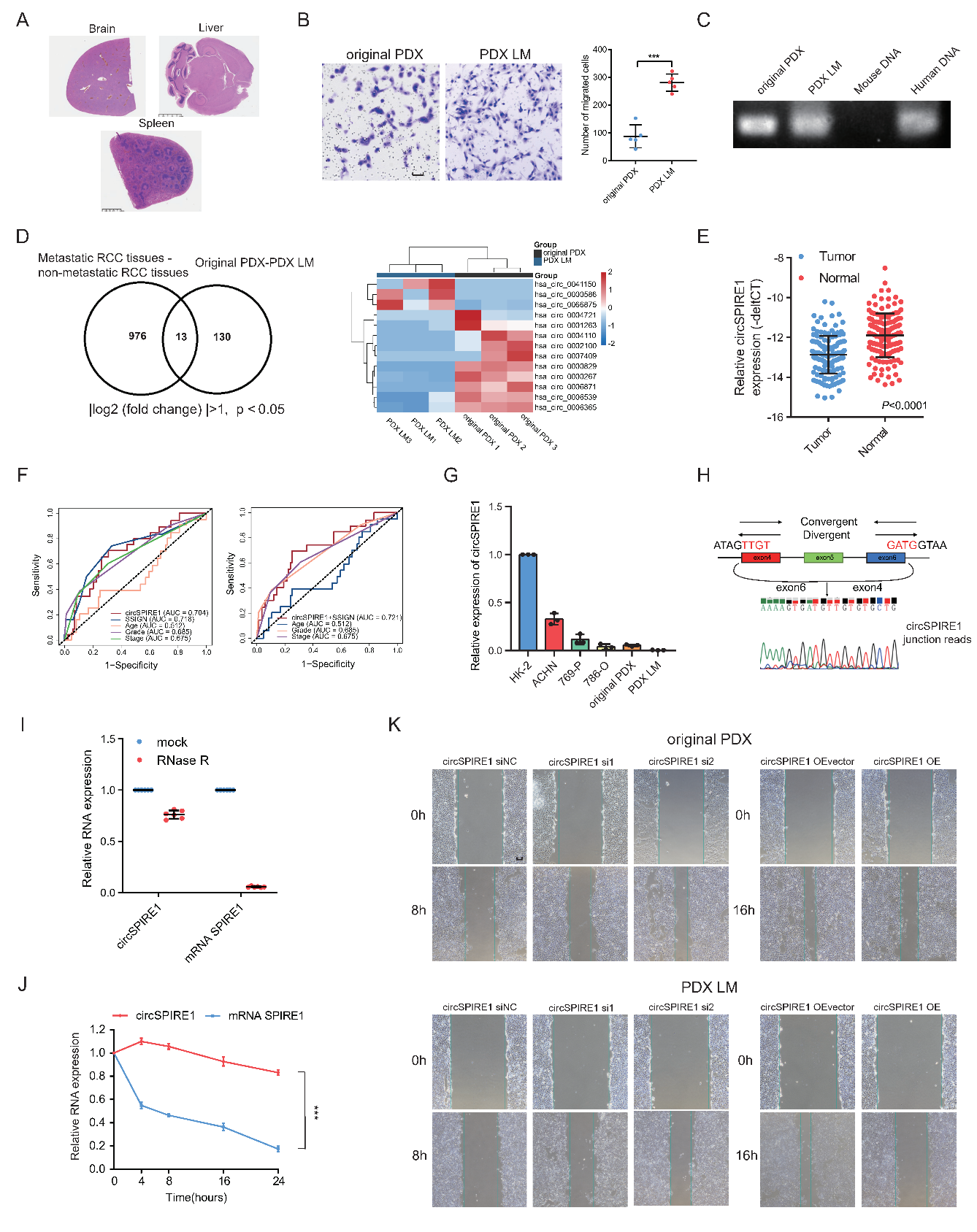


Fig. S1. Screening of potential regulatory circRNAs in metastatic RCC and confirming a circular RNA structure of circSPIRE1 and wound healing assay in vitro.

(A) HE staining of liver, brain and spleen during the PDX model constructing process. (B) Transwell assays showed increased migration ability of the PDX LM model compared to the original PDX model. Left, representative images. Scale bar, 100 µm. Right, histograms of invasive cell numbers (n=5/group). Data represent mean ± *S.D*.; dot plot reflects data points from independent experiments. The *P* values were determined by Student’s *t* test. (C) PCR data indicated the presence of human Alu sequence in both original PDX model and PDX LM model. (D) Left, Venn plot of the two datasets. Common circRNAs with *p* < 0.05, |log2 (fold change) | > 1 are chosen. Right, heatmap of 13 recurrently dysregulated circRNAs in both two datasets. (E) Expression of circSPIRE1 in RCC tissues and adjacent noncancer tissues was detected by qRT‑PCR (N =142). (F) Comparison of the circSPIRE1 with other known clinical prognostic biomarkers: ROC analyses of different prognostic biomarkers based on DFS. (G) qRT-PCR for the abundance of circSPIRE1 in different cell lines. Data represent mean ± *S.D*.; dot plots reflect data points from three independent experiments. (H) Schematic illustration of the circSPIRE1 formation. The back-splicing junction was verified by Sanger sequencing. Arrows represent divergent primers binding to the genome region of circSPIRE1. (I) Stability of circSPIRE1 and linear SPIRE1 assessed by RNase treatment followed by qRT-PCR. Data represent mean ± S.D.; dot plots reflect data points from six independent experiments. The *P* value was determined by Student’s *t* test. (J) Stability of circSPIRE1 and linear *SPIRE1* was assessed by Actinomycin D treatment followed by qRT-PCR at different time points. Data represent mean ± S.D.; dot plots reflect data points from six independent experiments. The *P* value was determined by two-way ANOVA. (K) Wound healing assays showed that expression of circSPIRE1 affected the migration ability of RCC cells. Scale bar, 100 µm.

**Figure S2**


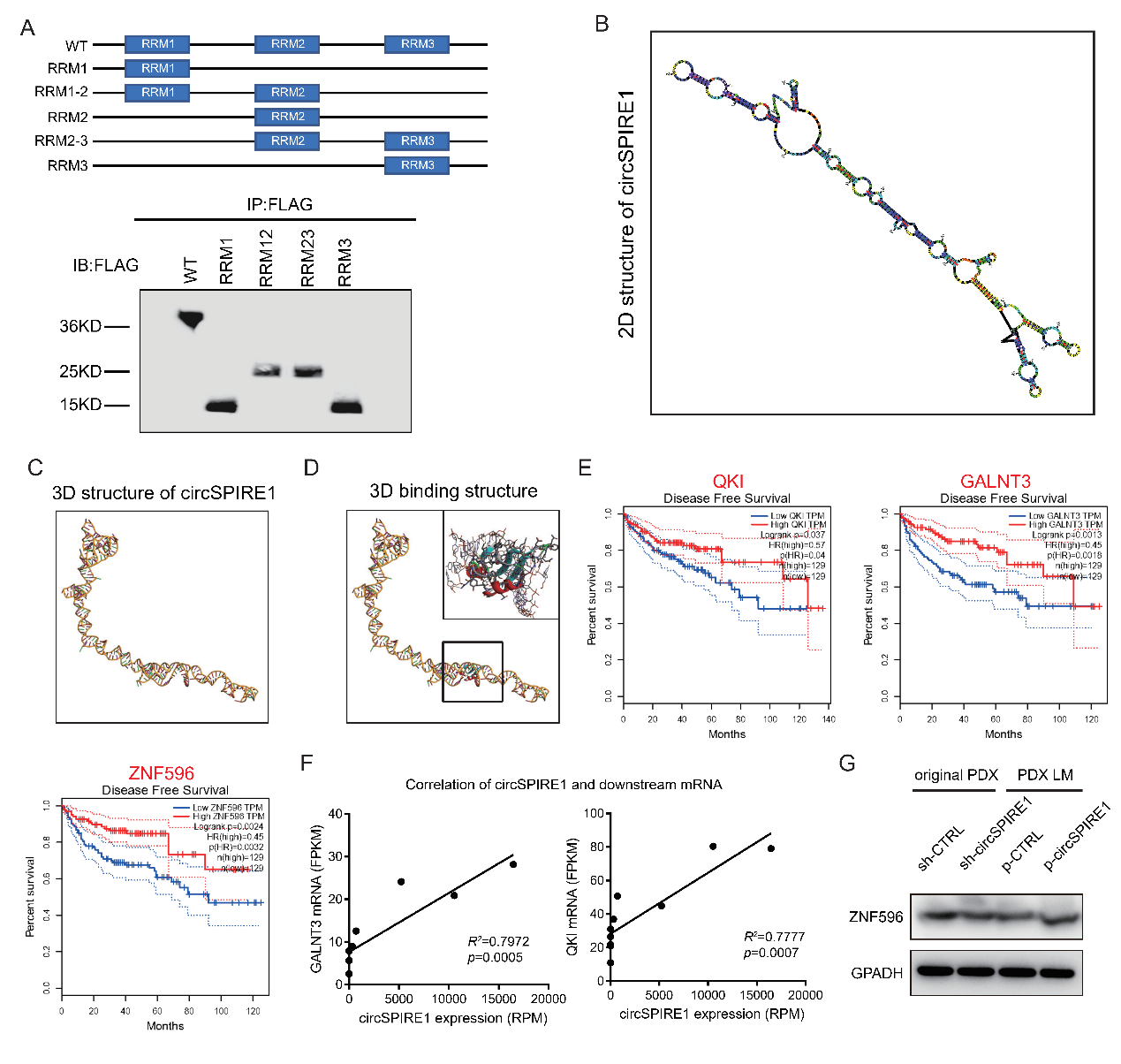


Fig. S2. Binding mechanism, and profile of downstream targets.

(A) Top, schematic structures showing RNA-binding domains within ELAVL1 protein and a summary of ELAVL1 truncations. Bottom, immunoblot analysis with anti-FLAG of cells transfected with plasmids encoding FLAG-tagged WT or truncated ELAVL1s. (B) 2-D RNA structure of circSPIRE1 generated by Mfold. (C) The 3-D structure of circSPIRE1 was generated by RNA Composer. (D) Graphical representation of the third scored three-dimensional structures of circSPIRE1 and ELAVL1 (RRM3 domain) docking models with a zoom-in image. (E) Kaplan−Meier DFS analysis of *GALNT3*, *QKI*, *ZNF596* RNA in RCC patients. (F) Liner correlation of circSPIRE1 and downstream mRNA expression in high throughput RNA sequencing result of five no clinical metastasis cases and five metastasis cases. (G) Western blotting validation of *ZNF596* upon circSPIRE1 knockdown and overexpression.

**Figure S3**


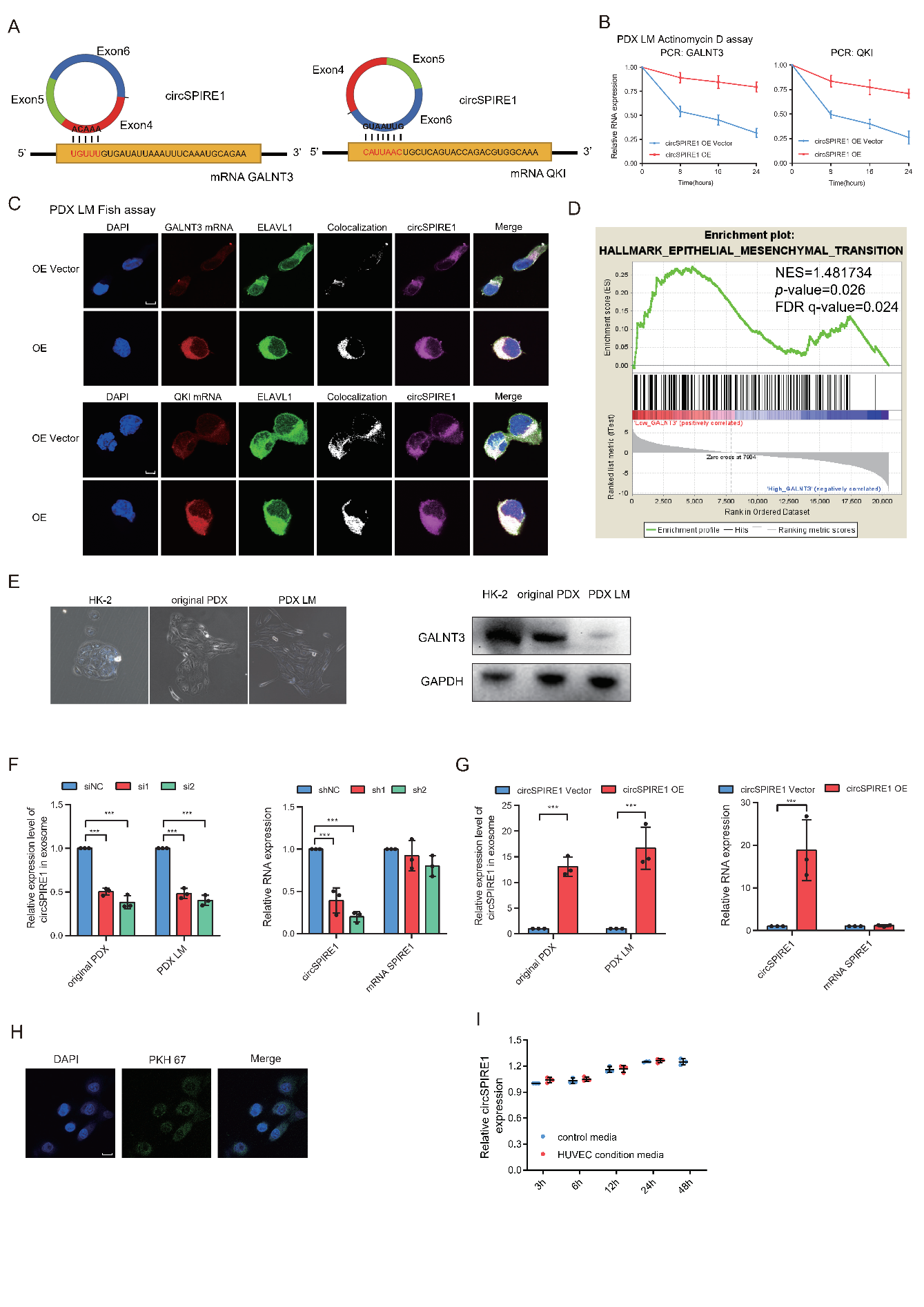


Fig. S3. Validation of downstream targeted pathway and exosome location of circSPIRE1.

(A) Schematic illustration of the binding between *GALNT3* as well as *QKI* and circSPIRE1. (B) Enhanced stability of *GALNT3* (left) and *QKI* (right) upon circSPIRE1 overexpression. (C) IF-FISH assay showed that the colocalization of circSPIRE1/ELAVL1/mRNA was increased upon overexpression of circSPIRE1. Scale bar, 10 µm. (D) GSEA analysis of EMT pathway in TCGA patients with high and low *GALNT3* expression (NES, normal enrichment score; FDR, false discovery rate). (E) Morphology and *GALNT3* protein expression of HK-2, original PDX, PDX LM cells. Scale bar, 10 µm. (F) Left, RT-PCR analysis of exosomal circSPIRE1 expression in knockdown cell lines. Right, circSPIRE1 and *SPIRE1* mRNA expression in knockdown cell lines. Data represent mean ± S.D.; dot plots reflect data points from three independent experiments. The *P*-values were determined by Student’s *t* test. (G) Left, RT-PCR analysis of exosomal circSPIRE1 expression in overexpression cell lines. Right, circSPIRE1 and *SPIRE1* mRNA expression in overexpression cell lines. Data represent mean ± *S.D*.; dot plots reflect data points from three independent experiments. The *P* values were determined by Student’s *t* test. (H) Presence of PKH67 lipid dye in HUVECs after adding PKH67 labeled exosomes derived from CM for 48 hours. (I) RT-PCR analysis of circSPIRE1 expression in original PDX cells incubated with exosomes derived from HUVECs for 3, 6, 12, 24, and 48 hours. Data represent mean ± S.D.; dot plots reflect data points from three independent experiments. The *P*-values were determined by Student’s *t* test.

**Figure S4**

**
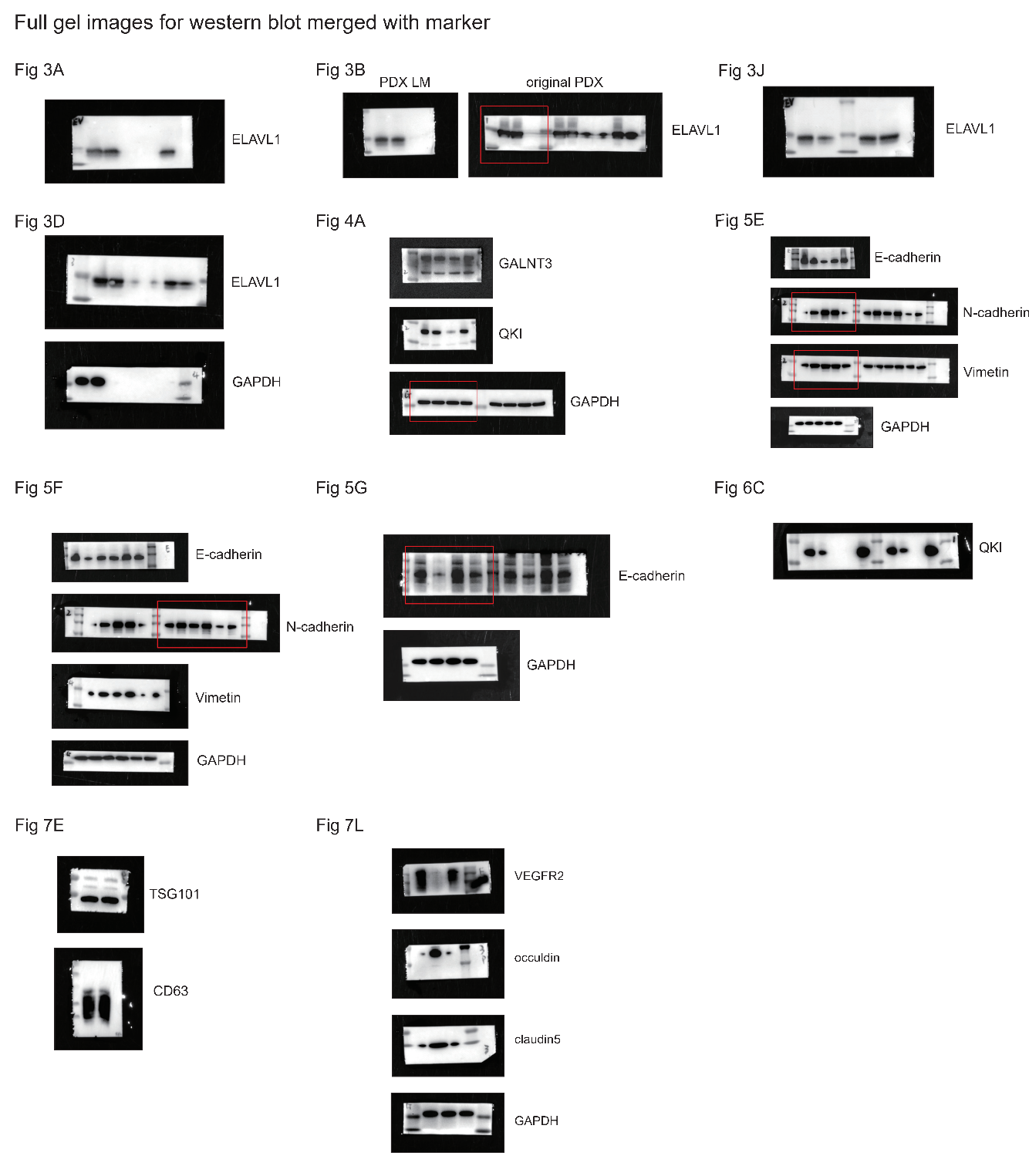
**

**Fig. S4**. **The marked size full gel images for western blot.**

**Figure S5**

**
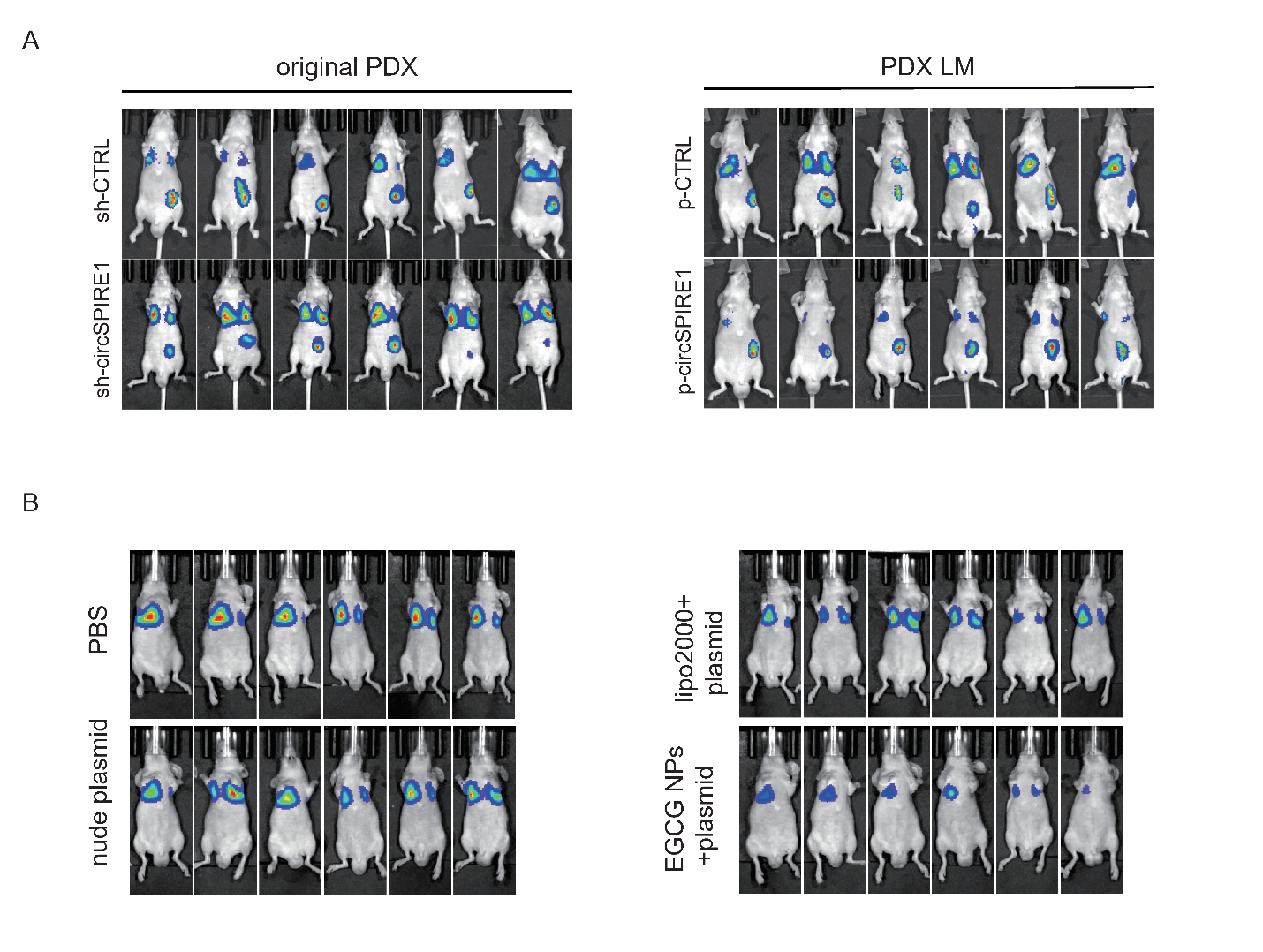
**

**Fig. S5**. **Bioluminescent images of all mice in the study.**

(A) Bioluminescent images of our orthotopic metastasis model. (B) Bioluminescent images of our treatment study.

Supplementary Tables

Table S1. Clinicopathological characteristics in 142 RCC patients with different hsa_circ_0000829 expression level

| Parameter | Total | CircSPIRE1 expression | | *P* value |
| --- | --- | --- | --- | --- |
|  |  | High | Low |  |
| Age(year) |  |  |  | 0.579 |
| <60 | 101(71.1) | 52(73.2) | 49(69.0) |  |
| ≥ 60 | 41(28.9) | 19(26.8) | 22(31.0) |  |
| Gender |  |  |  | 0.108 |
| Female | 47(33.1) | 28(39.4) | 19(26.8) |  |
| Male | 95(66.9) | 43(60.6) | 52(73.2) |  |
| TNM stage |  |  |  | <0.001^*^ |
| Ⅰ | 90(63.4) | 58(81.7) | 32(45.1) |  |
| Ⅱ+Ⅲ | 52(36.6) | 13(18.3) | 39(54.9) |  |
| Grade |  |  |  | 0.001^*^ |
| 1+2 | 115(81.0) | 65(91.0) | 50(69.8) |  |
| 3+4 | 27(19.0) | 6(9.0) | 21(30.2) |  |

Data were n (%). ^*^*P* value<0.05

Table S2. Univariate and multivariate Cox regression analyses of different parameters on disease-free survival

| Parameter | Univariate Analysis | | Multivariate Analysis | |
| --- | --- | --- | --- | --- |
|  | HR (95%*CI*) | *P* Value | HR (95%*CI*) | *P* Value |
| Age (≥ 60 vs. < 60 year) | 2.004(0.889,4.518) | 0.094 | - | - |
| Gender (Female vs. male) | 1.820(0.722,4.592) | 0.205 | - | - |
| TNM stage (II+III vs. I) | 2.921(1.239,6.886) | 0.014^*^ | 1.240(0.452,3.405) | 0.676 |
| Grade (3+ 4 vs. 1+ 2) | 3.278(1.393,7.716) | 0.007^*^ | 2.251(0.859,5.896) | 0.099 |
| Hsa_circ_0000829 expression (Low vs. high) | 3.859(1.524,9.768) | 0.004 | 3.089(1.131,8.438) | 0.028^*^ |

HR: hazard ratio; *CI*: confidence interval

| Protein_ID | Protein_Qscore | Protein_Mass | Coverage |
| --- | --- | --- | --- |
| sp\|Q9BQE3\|TBA1C_HUMAN | 3.575250631 | 49863.46151 | 0.0445 |
| sp\|P46779\|RL28_HUMAN | 3.575250631 | 15737.67076 | 0.0803 |
| sp\|P60709\|ACTB_HUMAN | 16.49151089 | 41709.72951 | 0.2107 |
| sp\|P26373\|RL13_HUMAN | 4.381016728 | 24246.5256 | 0.0948 |
| sp\|P21333\|FLNA_HUMAN | 2.075654076 | 280563.868 | 0.0068 |
| sp\|Q9NUU7\|DD19A_HUMAN | 10.72575189 | 53940.79351 | 0.0941 |
| sp\|Q96GA3\|LTV1_HUMAN | 2.190508364 | 54821.21904 | 0.0211 |
| sp\|P62979\|RS27A_HUMAN | 2.293648493 | 17953.49131 | 0.1218 |
| sp\|Q86Y46\|K2C73_HUMAN | 2.293648493 | 58886.7375 | 0.0222 |
| sp\|P51114\|FXR1_HUMAN | 3.575250631 | 69677.94265 | 0.0209 |
| sp\|O95831\|AIFM1_HUMAN | 21.45150379 | 66858.9054 | 0.1289 |
| sp\|P25705\|ATPA_HUMAN | 3.575250631 | 59713.59237 | 0.0235 |
| sp\|P02647\|APOA1_HUMAN | 16.59465102 | 30758.93196 | 0.2547 |
| sp\|Q06830\|PRDX1_HUMAN | 3.575250631 | 22096.27844 | 0.0503 |
| sp\|Q96HS1\|PGAM5_HUMAN | 2.293648493 | 31984.60677 | 0.0415 |
| sp\|Q7L2H7\|EIF3M_HUMAN | 3.575250631 | 42475.8044 | 0.0508 |
| sp\|O60506\|HNRPQ_HUMAN | 3.575250631 | 69559.59626 | 0.0209 |
| sp\|Q15717\|ELAV1_HUMAN | 10.72575189 | 36069.14952 | 0.1595 |
| sp\|O43790\|KRT86_HUMAN | 4.484156857 | 53466.29809 | 0.0309 |
| sp\|P08708\|RS17_HUMAN | 2.190508364 | 15540.3896 | 0.0667 |

Table S3. List of top 20 candidates of circSPIRE1-interacting proteins that were identified by RNA pull down and MS

Table S4. Quantifications of IF staining

**Quantification for Fig. 3C**

| circSPIRE1-ELAVL1 | | | | |
| --- | --- | --- | --- | --- |
|  | ROI | Area (pixels^2) | Pearson's Coeff. | Overlap |
| original PDX | Entire Slice | 1.05E+06 | 0.85043 | 0.83524 |
| PDX LM | Entire Slice | 1.05E+06 | 0.65039 | 0.6623 |

**Quantification for Fig. 4E**

| GALNT3 mRNA-ELAVL1 | | | | |
| --- | --- | --- | --- | --- |
|  | ROI | Area (pixels^2) | Pearson's Coeff. | Overlap |
| original PDX siNC | Entire Slice | 1.05E+06 | 0.804127 | 0.816751 |
| original PDX si1 | Entire Slice | 1.05E+06 | 0.459985 | 0.46418 |
| original PDX si2 | Entire Slice | 1.05E+06 | 0.328931 | 0.335818 |
| PDX LM OE Vector | Entire Slice | 1.05E+06 | 0.5390748 | 0.5411241 |
| PDX LM OE | Entire Slice | 1.05E+06 | 0.821874 | 0.836842 |

**Quantification for Fig. S3C**

| QKI mRNA-ELAVL1 | | | | |
| --- | --- | --- | --- | --- |
|  | ROI | Area (pixels^2) | Pearson's Coeff. | Overlap |
| original PDX siNC | Entire Slice | 1.05E+06 | 0.84365 | 0.86635 |
| original PDX si1 | Entire Slice | 1.05E+06 | 0.37477 | 0.38418 |
| original PDX si2 | Entire Slice | 1.05E+06 | 0.22702 | 0.230554 |
| PDX LM OE Vector | Entire Slice | 1.05E+06 | 0.634251 | 0.6442624 |
| PDX LM OE | Entire Slice | 1.05E+06 | 0.872394 | 0.889174 |

**Quantification for Fig. 5H**

| E-Cadherin-Giantin | | | | |
| --- | --- | --- | --- | --- |
|  | ROI | Area (pixels^2) | Pearson's Coeff. | Overlap |
| original PDX | Entire Slice | 1.05E+06 | 0.475 | 0.47911 |
| PDX LM | Entire Slice | 1.05E+06 | 0.5473 | 0.55418 |
| PDX LM+p-circSPIRE1+shGALNT3 NC | Entire Slice | 1.05E+06 | 0.32608 | 0.33818 |
| PDX LM+p-circSPIRE1+shGALNT3 | Entire Slice | 1.05E+06 | 0.5128 | 0.52971 |

Table S5. Contact distance and favorable bonds

Provided in supplementary table 5 contact distance.xlsx

Table S6. NCG result of PDX LM p-CTRL cells versus PDX LM p-circSPIRE1 cells

Provided in supplementary table 6 seq.xlsx

Table S7. *QKI* binding motif in intron3 from MEME SUITE

| motif_id | start | stop | strand | score | p-value | q-value | matched_sequence |
| --- | --- | --- | --- | --- | --- | --- | --- |
| WTACTAAC | 896 | 903 | + | 7.56471 | 0.000824 | 0.703 | UUACUUAC |
| WTACTAAC | 1073 | 1080 | + | 7.51176 | 0.000839 | 0.703 | AGAUUAAC |
| WTACTAAC | 2305 | 2312 | + | 7.3 | 0.000946 | 0.703 | AAAUUAAU |
| WTACTAAC | 3205 | 3212 | + | 7.48824 | 0.000854 | 0.703 | GUUCUAAC |
| WTACTAAC | 3868 | 3875 | + | 9.84706 | 0.000122 | 0.703 | AUAUUAAC |
| WTACTAAC | 3985 | 3992 | + | 7.70588 | 0.000778 | 0.703 | GCACUAAU |
| WTACTAAC | 4289 | 4296 | + | 8.47059 | 0.000473 | 0.703 | CUAUUAAU |
| WTACTAAC | 4699 | 4706 | + | 7.21176 | 0.000992 | 0.703 | UCAUUAAU |
| WTACTAAC | 5024 | 5031 | + | 8.80588 | 0.000366 | 0.703 | AUUCUAAC |
| WTACTAAC | 7387 | 7394 | + | 7.71765 | 0.000763 | 0.703 | UUACUCAC |
| WTACTAAC | 8588 | 8595 | + | 7.56471 | 0.000824 | 0.703 | UUACUUAC |
| WTACTAAC | 9772 | 9779 | + | 7.27059 | 0.000977 | 0.703 | UUACUGAC |
| WTACTAAC | 10862 | 10869 | + | 8.17647 | 0.000565 | 0.703 | CAACUAAU |

Table S8. *QKI* binding motif in intron6 from MEME SUITE

| motif_id | start | stop | strand | score | p-value | q-value | matched_sequence |
| --- | --- | --- | --- | --- | --- | --- | --- |
| WTACTAAC | 321 | 328 | + | 8.47059 | 0.000473 | 1 | CUAUUAAU |
| WTACTAAC | 3704 | 3711 | + | 8.47059 | 0.000473 | 1 | CUAUUAAU |
| WTACTAAC | 4127 | 4134 | + | 7.74118 | 0.000748 | 1 | UAAUUAAC |
| WTACTAAC | 4151 | 4158 | + | 7.21176 | 0.000992 | 1 | UCAUUAAU |
| WTACTAAC | 7375 | 7382 | + | 7.21176 | 0.000992 | 1 | UCAUUAAU |
| WTACTAAC | 8197 | 8204 | + | 7.56471 | 0.000824 | 1 | UUACUUAC |
| WTACTAAC | 10276 | 10283 | + | 8.89412 | 0.000336 | 1 | UCACUAAU |

Table S9. Correlation analysis of circSPIRE1 (red) expression in RCC epithelium and stroma (labeled by CK, purple) and their adjacent endothelial cells (labeled by CD34, green)

|  | FISH score of circSPIRE1 in epithelial cells/cancer cells |
| --- | --- |
| FISH score of circSPIRE1 in endothelial cells | *r*=0.672, *p*=0.003, *n*=10 |

Data S1. Intersected 13 dysregulated circular RNAs circular RNAs.

Data S2. Metastatic sites of 14 RCC patients with metastasis.

Data S3. Four dysregulated circular RNAs circular RNAs in metastatic RCC comparing with non-metastatic RCC.

Data S4. DFS information of circSPIRE1 and *GALNT3*.

Data S5. Primers, probes, and vectors used in this article.
