# Supplementary material for: Exosomal circSPIRE1 mediates glycosylation of E-cadherin to suppress metastasis of renal cell carcinoma
Source: Oncogene. 2023 Apr 12;42(22):1802–20. doi: 10.1038/s41388-023-02678-7 (PMC10238271; doi:10.1038/s41388-023-02678-7)
Supplement: Supplementary file 2 — Data S1 [file 41388_2023_2678_MOESM2_ESM.pdf]

Data S1. Intersected 13 dysregulated circular RNAs circular RNAs

circBase	original PDX 1	original PDX 2	original PDX 3	PDX LM1	PDX LM2	PDX LM3
hsa_circ_0000586	1.39E-17	1.39E-17	1.39E-17	0.00E+00	2.49E+02	1.93E+02
hsa_circ_0002100	6.80437262	20.68159803	27.21789071	1.39E-17	1.39E-17	1.39E-17
hsa_circ_0000829	6.759747889	7.767854986	6.767062197	1.39E-17	1.39E-17	1.39E-17
hsa_circ_0004110	2.765397915	11.60184367	8.138509051	1.39E-17	1.39E-17	1.39E-17
hsa_circ_0004721	37.43408127	0.049379911	0	1.39E-17	1.39E-17	1.39E-17
hsa_circ_0007409	0.055919063	9.052197032	13.65093237	1.39E-17	1.39E-17	1.39E-17
hsa_circ_0041150	1.39E-17	1.39E-17	1.39E-17	7.862750986	12.78350685	0.10837779
hsa_circ_0006871	5.409922317	3.919857495	6.795458083	1.39E-17	1.39E-17	1.39E-17
hsa_circ_0006539	17.49571462	12.95249134	13.56237213	0	7.167167011	0.087387032
hsa_circ_0001263	14.73648262	5.218444037	4.697887564	0	1.993221952	0.051140574
hsa_circ_0000267	29.60131477	24.56956931	20.35103347	9.431841748	8.224901212	10.1833822
hsa_circ_0066875	0.026400196	1.277275554	0.654206275	3.104382145	6.96106766	11.75893551
hsa_circ_0006365	9.452884537	10.35816989	10.85593841	1.536647372	5.13873244	1.801119305
