# Supplementary material for: Exosomal circSPIRE1 mediates glycosylation of E-cadherin to suppress metastasis of renal cell carcinoma
Source: Oncogene. 2023 Apr 12;42(22):1802–20. doi: 10.1038/s41388-023-02678-7 (PMC10238271; doi:10.1038/s41388-023-02678-7)
Supplement: Supplementary file 3 — Data S2 [file 41388_2023_2678_MOESM3_ESM.pdf]

Data S2. Metastatic sites of 14 RCC patients with metastasis.

Patient ID	metastasis site
106947	Lung
189939	Liver
193953	Bone
195967	Lung
204903	Colon
208328	Bone
210670	Lung
1012033	Lung
1021690	Right adrenal
1058553	Left hilar lymph node
1065022	Right hilar renal lymph node, left supraclavicular lymph node
1075717	Liver, lungs,
1087750	Brain, lung,
1091319	The right femur
