# Supplementary material for: Exosomal circSPIRE1 mediates glycosylation of E-cadherin to suppress metastasis of renal cell carcinoma
Source: Oncogene. 2023 Apr 12;42(22):1802–20. doi: 10.1038/s41388-023-02678-7 (PMC10238271; doi:10.1038/s41388-023-02678-7)
Supplement: Supplementary file 4 — Data S3 [file 41388_2023_2678_MOESM4_ESM.pdf]

Data S3. Four dysregulated circular RNAs circular RNAs in metastatic RCC comparing with non-metastatic RCC

circBase	original PDX 1	original PDX 2	original PDX 3	PDX LM1	PDX LM2	PDX LM3
hsa_circ_0041150	1.39E-17	1.39E-17	1.39E-17	7.86E+00	1.28E+01	1.08E-01
hsa_circ_0001263	14.73648262	5.218444037	4.697887564	0.00E+00	1.99E+00	5.11E-02
hsa_circ_0000829	6.759747889	7.767854986	6.767062197	1.39E-17	1.39E-17	1.39E-17
hsa_circ_0007409	0.055919063	9.052197032	13.65093237	1.39E-17	1.39E-17	1.39E-17

hsa_circ_0000829				hsa_circ_0007409				hsa_circ_0001263				hsa_circ_0041150			
patient with metastasis		patient without metastasis		patient with metastasis		patient without metastasis		patient with metastasis		patient without metastasis		patient with metastasis		patient without metastasis	
1	1	1	3.348078452	1	1	1	2.542068182	1	1	1	2.367522949	1	3.172513598	1	1
2	0.989565869	2	2.351639094	2	0.937871732	2	2.121372377	2	1.195425578	2	1.732420564	2	1.764265391	2	0.845530384
3	0.957760814	3	2.009263349	3	1.485481058	3	1.912066737	3	1.134270206	3	1.38137234	3	2.342698402	3	0.906261858
4	1.085266972	4	18.54982065	4	0.925624399	4	2.134210054	4	1.034278094	4	2.681697291	4	1.898997099	4	1.384571155
5	0.961726373	5	6.276672783	5	0.951473482	5	2.311224409	5	1.13510346	5	1.631754258	5	3.345277432	5	0.826452685
6	0.921537176	6	4.055837919	6	1.965223469	6	2.134270711	6	1.312800294	6	2.334511494	6	3.36424338	6	0.876421416
7	0.911860763	7	4.75682846	7	0.942325998	7	1.901532572	7	1.134286779	7	2.717224938	7	2.027118877	7	1.257212454
8	0.956210178	8	8.378352983	8	1.545213657	8	1.171483217	8	1.042308398	8	1.53420172	8	3.145746991	8	0.964521729
9	0.983248677	9	7.727490631	9	0.947835637	9	2.432542612	9	1.132518799	9	1.434790778	9	1.56459095	9	0.954290165
10	0.978912672	10	8.51496146	10	0.931788087	10	1.474320057	10	0.614234482	10	2.51987909	10	2.547852379	10	1.16750368
11	0.975260178	11	6.884411498	11	0.914230229	11	1.868124457	11	2.132709024	11	1.537529419	11	1.564839346	11	0.934251186
12	0.99624056	12	7.516181994	12	1.172551316	12	1.948699666	12	1.453456212	12	2.34527735	12	2.483910705	12	1.85643163
13	0.978526323	13	6.48302289	13	0.954127892	13	1.031734319	13	0.793421473	13	2.597577167	13	2.06453715	13	0.967560281
14	1.012565064	14	14.2214829	14	0.734222068	14	2.638342943	14	0.970403577	14	1.545293533	14	2.241814617	14	1.14527496
