# Supplementary material for: Exosomal circSPIRE1 mediates glycosylation of E-cadherin to suppress metastasis of renal cell carcinoma
Source: Oncogene. 2023 Apr 12;42(22):1802–20. doi: 10.1038/s41388-023-02678-7 (PMC10238271; doi:10.1038/s41388-023-02678-7)
Supplement: Supplementary file 5 — Data S4 [file 41388_2023_2678_MOESM5_ESM.pdf]

Data S4. DFS information of circSPIRE1.

id	fustat	gender	age	Grade	size	T-stage	N	M-stage	STAGE	futime	DFS(days)	DFS(weeks)	Recurrence status	circSPIRE1(low1 high0)
295	0	0	66.36986301	1	58	1	0	0	1	1031	1031	147	0	0
304	0	0	49.18630137	1	46	1	0	0	1	1482	1482	212	0	1
313	0	1	64.77534247	1	24	1	0	0	1	4242	4242	606	0	0
335	0	0	59.18082192	1	43	1	0	0	1	4175	4175	596	0	0
389	0	1	50.10684932	1	60	1	0	0	1	3909	3909	558	0	0
478	0	0	53.98082192	1	54	1	0	0	1	3808	3808	544	0	0
524	0	1	43.55616438	1	25	1	0	0	1	3646	3646	521	0	0
601	1	1	61.09863014	1	53	1	0	0	1	998	444	63	1	0
644	0	1	59.99309474	1	63	1	0	0	1	3354	3354	479	0	0
668	0	1	32.07542989	1	25.4	1	0	0	1	3307	3307	472	0	0
1153	0	0	54.81227238	1	30	1	0	0	1	2764	2764	395	0	0
1157	0	0	59.08898403	1	40	1	0	0	1	2562	2562	366	0	0
1227	0	0	70.25871386	1	44	1	0	0	1	2400	2400	343	0	0
1229	0	1	47.91088279	1	28	1	0	0	1	2514	2514	359	0	0
1231	0	1	64.19617581	1	65	1	0	0	1	2442	2442	349	0	0
1265	0	0	62.94383562	1	45	1	0	0	1	2438	2438	348	0	1
1489	0	1	64.21506868	1	32	1	0	0	1	2097	2097	300	0	0
1528	0	1	62.97142318	1	25	1	0	0	1	1904	1904	272	0	1
2315	0	0	53.46712422	1	15	1	0	0	1	1527	1527	218	0	1
3021	0	1	47.58213521	1	40	1	0	0	1	1191	1191	170	0	1
561	0	0	54.16164384	1	67	2	0	0	2	3561	3561	509	0	0
599	0	1	55.15616438	1	138	2	0	0	2	3514	3514	502	0	1
600	0	0	58.19726027	1	73	2	0	0	2	3514	3514	502	0	0
792	0	1	57.00678422	1	90	2	0	0	2	2688	2688	384	0	0
966	0	1	41.29041233	1	80	2	0	0	2	3000	3000	429	0	0
1148	0	1	60.03687214	1	112	2	0	0	2	2767	2295	328	1	1
1445	0	0	42.87855921	1	84	2	0	0	2	2038	1207	172	1	1
413	0	0	24.66027397	1	76	3	0	0	3	3924	3924	561	0	1
293	0	0	57.24109589	2	57	1	0	0	1	4308	4308	615	0	0
308	0	0	46.87945205	2	40	1	0	0	1	2693	2693	385	0	0
322	0	1	51.90958904	2	50	1	0	0	1	641	641	92	0	0
332	0	1	46.4	2	56	1	0	0	1	3694	3694	528	0	0
347	0	1	57.58356164	2	25	1	0	0	1	3550	3550	507	0	0
374	0	0	65.59452055	2	42	1	0	0	1	4030	4030	576	0	0
376	0	1	30.33424658	2	56	1	0	0	1	2451	2451	350	0	0
378	0	1	54.31506849	2	30	1	0	0	1	3926	3926	561	0	0
393	0	1	43.83287671	2	70	1	0	0	1	3902	3902	557	0	1

395	0	1	51.66575342	2	41	1	0	0	1	3988	3988	570	0	1
398	0	1	46.28767123	2	50	1	0	0	1	3889	3889	556	0	0
422	0	0	79.02191781	2	36	1	0	0	1	3928	3928	561	0	0
428	0	1	40.1260274	2	30	1	0	0	1	3416	3416	488	0	1
433	0	0	61.41917808	2	47	1	0	0	1	3808	3808	544	0	0
435	0	1	44.73150685	2	70	1	0	0	1	3895	3895	556	0	0
460	0	0	76.85753425	2	70	1	0	0	1	3843	3843	549	0	0
464	0	1	53.80273973	2	29	1	0	0	1	3836	3836	548	0	1
483	0	1	29.26027397	2	55	1	0	0	1	3770	3770	539	0	0
487	0	1	62.53424658	2	37	1	0	0	1	1377	1377	197	0	0
503	0	1	41.22465753	2	45	1	0	0	1	2489	2489	356	0	0
506	0	1	41.99452055	2	60	1	0	0	1	3688	3688	527	0	1
536	0	1	42.39452055	2	60	1	0	0	1	1277	1277	182	0	0
537	0	0	40.60547945	2	52	1	0	0	1	2400	2400	343	0	0
543	0	1	66.43835616	2	20	1	0	0	1	3101	3101	443	0	0
552	0	1	69.13972603	2	55	1	0	0	1	3092	3092	442	0	0
553	0	0	59.65205479	2	50	1	0	0	1	3491	3491	499	0	0
569	0	0	53.18356164	2	40	1	0	0	1	3454	3454	493	0	0
651	0	1	39.43961362	2	70	1	0	0	1	3424	3424	489	0	0
654	0	1	31.37420153	2	31	1	0	0	1	2922	2922	417	0	1
681	0	0	50.23447534	2	60	1	0	0	1	2868	2868	410	0	0
746	0	1	42.7274263	2	47	1	0	0	1	3238	3238	463	0	0
832	0	1	48.99726205	2	50	1	0	0	1	3139	3139	448	0	0
944	0	0	36.61095929	2	20	1	0	0	1	3026	3026	432	0	0
1164	0	1	32.76841764	2	55	1	0	0	1	2749	2749	393	0	0
1237	0	0	38.33869959	2	44	1	0	0	1	2502	2502	357	0	0
1315	0	1	57.82625584	2	18	1	0	0	1	2352	2352	336	0	1
1316	0	0	26.6622146	2	60	1	0	0	1	2352	2352	336	0	0
1380	0	0	23.61493567	2	50	1	0	0	1	2293	2293	328	0	1
1442	0	0	31.4015411	2	50	1	0	0	1	2159	2159	308	0	0
1594	1	0	39.89231444	2	40	1	0	0	1	1657	1657	237	1	0
1610	0	1	64.3737443	2	30	1	0	0	1	1915	1915	274	0	1
1614	0	1	46.75616438	2	40	1	0	0	1	1665	1665	238	0	1
1621	0	1	41.42637112	2	35	1	0	0	1	1814	1814	259	1	1
1625	0	1	34.42859589	2	55	1	0	0	1	1925	1925	275	0	0
2056	0	1	37.6622146	2	25	1	0	0	1	1659	1659	237	0	1
2067	0	1	57.42627474	2	55	1	0	0	1	1657	1657	237	0	1
2697	0	1	46.43700622	2	40	1	0	0	1	1333	1333	190	1	1
3011	0	1	50.78007992	2	50	1	0	0	1	156	121	17	0	1
3025	0	1	39.81290096	2	40	1	0	0	1	1251	1251	179	0	0

3033	0	1	66.06196863	2	65	1	0	0	1	1187	1187	170	0	0
3034	0	1	36.63415145	2	32	1	0	0	1	1192	1192	170	0	1
3063	0	1	58.97465753	2	53	1	0	0	1	1236	1236	177	0	1
3067	0	1	52.04828833	2	55	1	0	0	1	1232	895	128	1	0
3128	0	1	70.70262556	2	45	1	0	0	1	1136	1136	162	0	1
3333	0	1	54.85334888	2	22	1	0	0	1	714	714	102	0	1
3352	0	0	43.26153044	2	45	1	0	0	1	881	881	126	1	0
3539	0	1	32.46982532	2	48	1	0	0	1	890	890	127	0	1
4100	0	1	44.28152622	2	45	1	0	0	1	725	725	104	0	0
4115	0	1	43.25953367	2	50	1	0	0	1	546	546	78	0	1
4704	0	1	56.78779619	2	40	1	0	0	1	263	263	38	0	0
244	0	0	50.79726027	2	156	2	0	0	2	2616	2616	374	0	1
392	0	1	33.63287671	2	88	2	0	0	2	3909	3909	558	0	1
458	0	0	52.87123288	2	80	2	0	0	2	3857	3857	551	0	1
481	1	0	56.05479452	2	105	2	0	0	2	937	97	14	0	0
485	0	1	57.98082192	2	135	2	0	0	2	3084	3084	441	0	0
494	0	1	73.96438356	2	78	2	0	0	2	3729	3729	533	0	0
529	1	1	64.89041096	2	134	2	0	0	2	684	253	36	1	1
649	0	1	50.83136603	2	138	2	0	0	2	3438	3438	491	0	1
691	0	1	46.70959038	2	80	2	0	0	2	3242	2528	361	1	1
787	0	1	39.45878995	2	101	2	0	0	2	3178	3178	454	0	0
799	0	1	55.9890411	2	156	2	0	0	2	2593	2593	370	0	1
1038	0	1	68.94366512	2	80	2	0	0	2	2878	1498	214	1	1
1297	0	0	80.58207797	2	75	2	0	0	2	2494	2494	356	0	1
303	0	1	70.64657534	2	78	3	0	0	3	4285	4285	612	0	1
371	1	1	48.21917808	2	58	3	0	0	3	863	863	123	1	1
399	0	0	61.95890411	2	69	3	0	0	3	3889	3889	556	0	1
431	0	1	62.01369863	2	70	3	0	0	3	660	660	94	0	0
441	1	1	61.72328767	2	33	3	0	0	3	2346	2097	300	1	1
504	0	0	54.98082192	2	140	3	0	0	3	3690	3690	527	0	1
650	1	1	21.60667833	2	77	2	0	0	3	2159	960	137	1	0
1233	0	1	71.5849329	2	50	1	0	0	3	290	290	41	0	0
3036	0	1	50.26731353	2	65	3	0	0	3	1222	1222	175	1	1
3083	0	1	40.974678	2	20	3	0	0	3	1179	1179	168	0	1
3085	0	0	18.30262556	2	130	3	0	0	3	1253	1253	179	0	1
3110	0	1	60.34927737	2	63	3	0	0	3	963	963	138	0	1
3123	0	1	60.05932356	2	30	3	1	0	3	1139	1139	163	1	1
3241	0	1	50.16837948	2	62	3	1	0	3	908	908	130	0	1
286	1	1	64.14246575	3	68	1	0	0	1	1638	584	83	1	0
810	0	0	42.93036707	3	25	1	0	0	1	2969	2969	424	0	0

868	0	1	80.62076003	3	60	1	0	0	1	89	89	13	0	1
1139	1	1	46.40547945	3	50	1	0	0	1	731	731	104	0	1
1223	0	0	49.66575342	3	40	1	0	0	1	2617	841	120	1	1
1306	0	1	59.83732937	3	30	1	0	0	1	2181	2181	312	0	0
1523	0	0	64.61643836	3	60	1	0	0	1	2032	2032	290	0	0
1527	0	1	63.41232877	3	35	1	0	0	1	2028	2028	290	0	1
3311	0	0	53.10279682	3	35	1	0	0	1	1076	1076	154	0	1
416	0	0	29.84657534	3	110	2	0	0	2	3939	3939	563	0	0
1230	0	0	49.32726515	3	73	2	0	0	2	2398	2398	343	0	1
1607	1	0	34.44657534	3	150	2	0	0	2	2019	230	33	1	1
3257	0	1	53.96301405	3	81	2	0	0	2	1083	1083	155	0	1
387	0	1	60.77260274	3	80	3	0	0	3	4022	4022	575	0	1
834	0	1	60.84383701	3	120	2	0	0	3	1591	1591	227	0	1
1441	0	1	55.5492009	3	80	3	0	0	3	2288	2288	327	0	1
3095	1	1	61.08698764	3	92	3	0	0	3	167	167	24	1	1
3113	1	1	44.99594882	3	119	3	0	0	3	727	727	104	0	0
3115	0	0	60.88624611	3	90	3	0	0	3	1234	1234	176	0	1
3119	0	1	48.34667123	3	75	3	0	0	3	1232	1232	176	0	1
815	1	1	41.70865901	4	47	1	0	0	1	1340	214	31	1	1
1605	0	1	66.57808219	4	95	2	0	0	2	425	149	21	1	1
446	1	1	44.87945205	4	100	3	0	0	3	2461	1478	211	1	1
755	0	0	56.44573819	4	60	3	0	0	3	3211	3211	459	0	1
757	0	0	73.22311737	4	70	3	0	0	3	1664	1664	238	1	1
3116	1	1	60.1934934	4	102	3	0	0	3	414	414	59	0	1
3350	0	1	52.21820953	4	64	3	1	0	3	704	704	101	0	1

Data S4. DFS information of GALNT3.

id	fustat	gender	age	Grade	size	T-stage	N	M-stage	STAGE	futime	DFS(days)	DFS(weeks)	Recurrence status	GALNT3(low1 high0)
295	0	0	66.36986301	1	58	1	0	0	1	1031	1031	147	0	0
304	0	0	49.18630137	1	46	1	0	0	1	1482	1482	212	0	1
313	0	1	64.77534247	1	24	1	0	0	1	4242	4242	606	0	0
335	0	0	59.18082192	1	43	1	0	0	1	4175	4175	596	0	0
389	0	1	50.10684932	1	60	1	0	0	1	3909	3909	558	0	0
478	0	0	53.98082192	1	54	1	0	0	1	3808	3808	544	0	0
524	0	1	43.55616438	1	25	1	0	0	1	3646	3646	521	0	0
601	1	1	61.09863014	1	53	1	0	0	1	998	444	63	1	1
644	0	1	59.99309474	1	63	1	0	0	1	3354	3354	479	0	0
668	0	1	32.07542989	1	25.4	1	0	0	1	3307	3307	472	0	0
1153	0	0	54.81227238	1	30	1	0	0	1	2764	2764	395	0	0
1157	0	0	59.08898403	1	40	1	0	0	1	2562	2562	366	0	0
1227	0	0	70.25871386	1	44	1	0	0	1	2400	2400	343	0	0
1229	0	1	47.91088279	1	28	1	0	0	1	2514	2514	359	0	0
1231	0	1	64.19617581	1	65	1	0	0	1	2442	2442	349	0	0
1265	0	0	62.94383562	1	45	1	0	0	1	2438	2438	348	0	1
1489	0	1	64.21506868	1	32	1	0	0	1	2097	2097	300	0	0
1528	0	1	62.97142318	1	25	1	0	0	1	1904	1904	272	0	1
2315	0	0	53.46712422	1	15	1	0	0	1	1527	1527	218	0	1
3021	0	1	47.58213521	1	40	1	0	0	1	1191	1191	170	0	1
561	0	0	54.16164384	1	67	2	0	0	2	3561	3561	509	0	0
599	0	1	55.15616438	1	138	2	0	0	2	3514	3514	502	0	1
600	0	0	58.19726027	1	73	2	0	0	2	3514	3514	502	0	0
792	0	1	57.00678422	1	90	2	0	0	2	2688	2688	384	0	0
966	0	1	41.29041233	1	80	2	0	0	2	3000	3000	429	0	0
1148	0	1	60.03687214	1	112	2	0	0	2	2767	2295	328	1	1
1445	0	0	42.87855921	1	84	2	0	0	2	2038	1207	172	1	0
413	0	0	24.66027397	1	76	3	0	0	3	3924	3924	561	0	1
293	0	0	57.24109589	2	57	1	0	0	1	4308	4308	615	0	0
308	0	0	46.87945205	2	40	1	0	0	1	2693	2693	385	0	0
322	0	1	51.90958904	2	50	1	0	0	1	641	641	92	0	0
332	0	1	46.4	2	56	1	0	0	1	3694	3694	528	0	0
347	0	1	57.58356164	2	25	1	0	0	1	3550	3550	507	0	0
374	0	0	65.59452055	2	42	1	0	0	1	4030	4030	576	0	0
376	0	1	30.33424658	2	56	1	0	0	1	2451	2451	350	0	0
378	0	1	54.31506849	2	30	1	0	0	1	3926	3926	561	0	0

393	0	1	43.83287671	2	70	1	0	0	1	3902	3902	557	0	1
395	0	1	51.66575342	2	41	1	0	0	1	3988	3988	570	0	1
398	0	1	46.28767123	2	50	1	0	0	1	3889	3889	556	0	0
422	0	0	79.02191781	2	36	1	0	0	1	3928	3928	561	0	0
428	0	1	40.1260274	2	30	1	0	0	1	3416	3416	488	0	1
433	0	0	61.41917808	2	47	1	0	0	1	3808	3808	544	0	0
435	0	1	44.73150685	2	70	1	0	0	1	3895	3895	556	0	0
460	0	0	76.85753425	2	70	1	0	0	1	3843	3843	549	0	1
464	0	1	53.80273973	2	29	1	0	0	1	3836	3836	548	0	1
483	0	1	29.26027397	2	55	1	0	0	1	3770	3770	539	0	0
487	0	1	62.53424658	2	37	1	0	0	1	1377	1377	197	0	0
503	0	1	41.22465753	2	45	1	0	0	1	2489	2489	356	0	0
506	0	1	41.99452055	2	60	1	0	0	1	3688	3688	527	0	0
536	0	1	42.39452055	2	60	1	0	0	1	1277	1277	182	0	0
537	0	0	40.60547945	2	52	1	0	0	1	2400	2400	343	0	1
543	0	1	66.43835616	2	20	1	0	0	1	3101	3101	443	0	0
552	0	1	69.13972603	2	55	1	0	0	1	3092	3092	442	0	0
553	0	0	59.65205479	2	50	1	0	0	1	3491	3491	499	0	0
569	0	0	53.18356164	2	40	1	0	0	1	3454	3454	493	0	0
651	0	1	39.43961362	2	70	1	0	0	1	3424	3424	489	0	0
654	0	1	31.37420153	2	31	1	0	0	1	2922	2922	417	0	1
681	0	0	50.23447534	2	60	1	0	0	1	2868	2868	410	0	0
746	0	1	42.7274263	2	47	1	0	0	1	3238	3238	463	0	0
832	0	1	48.99726205	2	50	1	0	0	1	3139	3139	448	0	0
944	0	0	36.61095929	2	20	1	0	0	1	3026	3026	432	0	0
1164	0	1	32.76841764	2	55	1	0	0	1	2749	2749	393	0	0
1237	0	0	38.33869959	2	44	1	0	0	1	2502	2502	357	0	0
1315	0	1	57.82625584	2	18	1	0	0	1	2352	2352	336	0	1
1316	0	0	26.6622146	2	60	1	0	0	1	2352	2352	336	0	0
1380	0	0	23.61493567	2	50	1	0	0	1	2293	2293	328	0	1
1442	0	0	31.4015411	2	50	1	0	0	1	2159	2159	308	0	0
1594	1	0	39.89231444	2	40	1	0	0	1	1657	1657	237	1	1
1610	0	1	64.3737443	2	30	1	0	0	1	1915	1915	274	0	1
1614	0	1	46.75616438	2	40	1	0	0	1	1665	1665	238	0	1
1621	0	1	41.42637112	2	35	1	0	0	1	1814	1814	259	1	1
1625	0	1	34.42859589	2	55	1	0	0	1	1925	1925	275	0	0
2056	0	1	37.6622146	2	25	1	0	0	1	1659	1659	237	0	1
2067	0	1	57.42627474	2	55	1	0	0	1	1657	1657	237	0	1

2697	0	1	46.43700622	2	40	1	0	0	1	1333	1333	190	1	1
3011	0	1	50.78007992	2	50	1	0	0	1	156	121	17	0	1
3025	0	1	39.81290096	2	40	1	0	0	1	1251	1251	179	0	1
3033	0	1	66.06196863	2	65	1	0	0	1	1187	1187	170	0	0
3034	0	1	36.63415145	2	32	1	0	0	1	1192	1192	170	0	1
3063	0	1	58.97465753	2	53	1	0	0	1	1236	1236	177	0	1
3067	0	1	52.04828833	2	55	1	0	0	1	1232	895	128	1	0
3128	0	1	70.70262556	2	45	1	0	0	1	1136	1136	162	0	1
3333	0	1	54.85334888	2	22	1	0	0	1	714	714	102	0	1
3352	0	0	43.26153044	2	45	1	0	0	1	881	881	126	1	0
3539	0	1	32.46982532	2	48	1	0	0	1	890	890	127	0	1
4100	0	1	44.28152622	2	45	1	0	0	1	725	725	104	0	0
4115	0	1	43.25953367	2	50	1	0	0	1	546	546	78	0	1
4704	0	1	56.78779619	2	40	1	0	0	1	263	263	38	0	0
244	0	0	50.79726027	2	156	2	0	0	2	2616	2616	374	0	1
392	0	1	33.63287671	2	88	2	0	0	2	3909	3909	558	0	1
458	0	0	52.87123288	2	80	2	0	0	2	3857	3857	551	0	1
481	1	0	56.05479452	2	105	2	0	0	2	937	97	14	0	0
485	0	1	57.98082192	2	135	2	0	0	2	3084	3084	441	0	0
494	0	1	73.96438356	2	78	2	0	0	2	3729	3729	533	0	0
529	1	1	64.89041096	2	134	2	0	0	2	684	253	36	1	1
649	0	1	50.83136603	2	138	2	0	0	2	3438	3438	491	0	1
691	0	1	46.70959038	2	80	2	0	0	2	3242	2528	361	1	1
787	0	1	39.45878995	2	101	2	0	0	2	3178	3178	454	0	0
799	0	1	55.9890411	2	156	2	0	0	2	2593	2593	370	0	1
1038	0	1	68.94366512	2	80	2	0	0	2	2878	1498	214	1	1
1297	0	0	80.58207797	2	75	2	0	0	2	2494	2494	356	0	1
303	0	1	70.64657534	2	78	3	0	0	3	4285	4285	612	0	1
371	1	1	48.21917808	2	58	3	0	0	3	863	863	123	1	1
399	0	0	61.95890411	2	69	3	0	0	3	3889	3889	556	0	1
431	0	1	62.01369863	2	70	3	0	0	3	660	660	94	0	0
441	1	1	61.72328767	2	33	3	0	0	3	2346	2097	300	1	1
504	0	0	54.98082192	2	140	3	0	0	3	3690	3690	527	0	1
650	1	1	21.60667833	2	77	2	0	0	3	2159	960	137	1	0
1233	0	1	71.5849329	2	50	1	0	0	3	290	290	41	0	1
3036	0	1	50.26731353	2	65	3	0	0	3	1222	1222	175	1	1
3083	0	1	40.974678	2	20	3	0	0	3	1179	1179	168	0	1
3085	0	0	18.30262556	2	130	3	0	0	3	1253	1253	179	0	0

3110	0	1	60.34927737	2	63	3	0	0	3	963	963	138	0	1
3123	0	1	60.05932356	2	30	3	1	0	3	1139	1139	163	1	1
3241	0	1	50.16837948	2	62	3	1	0	3	908	908	130	0	1
286	1	1	64.14246575	3	68	1	0	0	1	1638	584	83	1	0
810	0	0	42.93036707	3	25	1	0	0	1	2969	2969	424	0	0
868	0	1	80.62076003	3	60	1	0	0	1	89	89	13	0	1
1139	1	1	46.40547945	3	50	1	0	0	1	731	731	104	0	1
1223	0	0	49.66575342	3	40	1	0	0	1	2617	841	120	1	1
1306	0	1	59.83732937	3	30	1	0	0	1	2181	2181	312	0	0
1523	0	0	64.61643836	3	60	1	0	0	1	2032	2032	290	0	0
1527	0	1	63.41232877	3	35	1	0	0	1	2028	2028	290	0	1
3311	0	0	53.10279682	3	35	1	0	0	1	1076	1076	154	0	1
416	0	0	29.84657534	3	110	2	0	0	2	3939	3939	563	0	0
1230	0	0	49.32726515	3	73	2	0	0	2	2398	2398	343	0	1
1607	1	0	34.44657534	3	150	2	0	0	2	2019	230	33	1	1
3257	0	1	53.96301405	3	81	2	0	0	2	1083	1083	155	0	1
387	0	1	60.77260274	3	80	3	0	0	3	4022	4022	575	0	0
834	0	1	60.84383701	3	120	2	0	0	3	1591	1591	227	0	1
1441	0	1	55.5492009	3	80	3	0	0	3	2288	2288	327	0	1
3095	1	1	61.08698764	3	92	3	0	0	3	167	167	24	1	0
3113	1	1	44.99594882	3	119	3	0	0	3	727	727	104	0	0
3115	0	0	60.88624611	3	90	3	0	0	3	1234	1234	176	0	1
3119	0	1	48.34667123	3	75	3	0	0	3	1232	1232	176	0	1
815	1	1	41.70865901	4	47	1	0	0	1	1340	214	31	1	1
1605	0	1	66.57808219	4	95	2	0	0	2	425	149	21	1	1
446	1	1	44.87945205	4	100	3	0	0	3	2461	1478	211	1	0
755	0	0	56.44573819	4	60	3	0	0	3	3211	3211	459	0	1
757	0	0	73.22311737	4	70	3	0	0	3	1664	1664	238	1	1
3116	1	1	60.1934934	4	102	3	0	0	3	414	414	59	0	1
3350	0	1	52.21820953	4	64	3	1	0	3	704	704	101	0	1
