# Supplementary material for: Exosomal circSPIRE1 mediates glycosylation of E-cadherin to suppress metastasis of renal cell carcinoma
Source: Oncogene. 2023 Apr 12;42(22):1802–20. doi: 10.1038/s41388-023-02678-7 (PMC10238271; doi:10.1038/s41388-023-02678-7)
Supplement: Supplementary file 6 — Data S5 [file 41388_2023_2678_MOESM6_ESM.pdf]

Data S5. Si & sh sequence used in our experiement

Gene name	sequence (5’→3’)	
	sh1	sh2
circSPIRE1	TTGCGAAAAGTGATGTTGT	TGCGAAAAGTGATGTTGTG
GALNT3	GCATCCAAGTTTAGTGTCA	GTGACAGGATTTCTTTGCA
QKI	GAAGCTGGTTTAATCTATA	GACCTATTGTTCAGTTACA

Data S5. Primers used in our experiement

Primer name(F, forward; R, reverse)	qPCR primer sequence (5'→3')
circSPIRE1 divergent F	TCGCTGCAAAAGATACACCT
circSPIRE1 divergent R	GAGATGAGCAGCACACAACAT
circSPIRE1 convergent F	GCACGATTCTGGGTACAGGT
circSPIRE1 convergent R	GTTGTACTGCCGCTCTTGGA
qPCR SPIRE1mRNA F	TGAAACCAACTCCACCACGG
qPCR SPIRE1mRNA R	GTGGCCGCATTGCTAATCTG
circSPIRE1 divergent2 F for sanger sequence	TCCAAGAGCGGCAGTACAA
circSPIRE1 divergent2 R for sanger sequence	TGCCTGATAATGATTTGGTGCA
QKI mRNA F	CTGATGCTGTGGGACCTATTG
QKI mRNA R	GTTGTTTGGCTGTAAGTCCTCT
GALNT3 mRNA F	TCTTGGACCAGACACTCGAC
GALNT3 mRNA R	GGACAGTTCTAAGCAACGTGG
pre mRNA intron3 1F	TGTATCAGCTTGACTGGGCA
pre mRNA intron3 1R	CAGCTGTGACACTTGAGATGC
pre mRNA intron3 2F	TCTGCAGACCACAAATGACCA
pre mRNA intron3 2R	CCAACACACACACCACCAAAAT
pre mRNA intron3 3F	TCCACATCTGTGACTGTTCTGA
pre mRNA intron3 3R	ACCTGCTAACCAAACCACAAAC
pre mRNA intron3 4F	AGGTAGATTGGGCAGGCATT
pre mRNA intron3 4R	GCACCATTACTGCCCACTG
pre mRNA intron3 5F	GGAAAGCCACTAAAGCCTAACC
pre mRNA intron3 5R	TTTCTTAGCTCCACCTGCCC
pre mRNA intron3 6F	TGCCCCAAGAGTCAAAGAAGA
pre mRNA intron3 6R	TCAAGCGATCTACCTGCCTT
pre mRNA intron3 7F	ACAGGGAAGACGTTCTGAGC
pre mRNA intron3 7R	AGTGGCTCCCACTGAACTTA
pre mRNA intron3 8F	CACCACACTTGGCCTATTTTTCTT
pre mRNA intron3 8R	TGAACAAGTCAACATCCCGTT
pre mRNA intron3 9F	AGACTGCTCCCTCAAAAACCT
pre mRNA intron3 9R	ATCAGGGGTTAGGGGGACAAAA
pre mRNA intron3 10F	GGTAAAGCCTCAGTTGTTGTCT
pre mRNA intron3 10R	ACTCACAGAGCCACTAGTAAACAG
pre mRNA intron3 11F	GCTACTGTGAAGAAGGTTTCCT
pre mRNA intron3 11R	TGTGGCTTTCTGTTTTCACG
pre mRNA intron6 1F	GCAAATTCTGACAGCTCTCCC
pre mRNA intron6 1R	GGGAAGTCGAGGCTACAGTAG
pre mRNA intron6 2F	TGTGCTCAAGTTTTCCCACAT
pre mRNA intron6 2R	TCAGGCCCATGTTCTTGTCAAA
pre mRNA intron6 3F	ACTGGGTGTCTTTTGGGGTT
pre mRNA intron6 3R	ACGTCCTTCCAGAGTCAGAAT
pre mRNA intron6 4F	TGCAAAGAGCAGGCAGTGTT
pre mRNA intron6 4R	TGTCACTACTGCCTCACCTACT
pre mRNA intron6 5F	TGCTGATTTAACAGTCCTCAGC
pre mRNA intron6 5R	TCCATTTTGGATGCACAGTTC
pre mRNA intron6 6F	CCCCTTATCCCCTGGTAACC
pre mRNA intron6 6R	AGGAGCAAAGTACCTCAGCA
pre mRNA intron6 7F	CATGTGCTTGTGAACCATTTGT
pre mRNA intron6 7R	GGTACGCTAGAAACCCAGTC
pre mRNA intron6 8F	TGCAATCCAACCCTTTGTGG
pre mRNA intron6 8R	TGTAATCCCAACTGCACCCT
pre mRNA intron6 9F	TCCTCCACCCGTACTGACTT
pre mRNA intron6 9R	GGCCAACCCTATACGTTCCA
pre mRNA intron6 10F	TGACTGTGCCTTCTAAGTGGTC
pre mRNA intron6 10R	TCTGCCTCCATCCACAAAACT
U3 F	TTCTCTGAGCGTGTAGAGCACCGA
U3 R	GATCATCAATGGCTGACGGCAGTT

Data S5. Probe used in our experiement

Probe name	sequence (5’→3’)
circSPIRE1 e6+4	5'Biotin-TGATTCAGTAGGGAGATGAGCAGCACACAACATCACTTTTCGCAAGGTGTATCTTTTGCA-3’
SPIRE1 e4	5'Biotin-CACGACATACTGCCTGATAATGATTTGGTGCATCTGATTCAGTAGGGAGATGAGCAGCA-3’
GAPDH probe	5′-UAUCCACUUUACCAGAGUUAAAAGCAGCCCUGGUGACCAGGCGCCCAAUACGACCAAA-3′
GALNT3 mRNA probe e2-e3	GCCAACAGAGGTTCTAGCCAACCATAGAAACACTCACAGTGAGCATC
QKI mRNA CDS probe e2-e3	TCTTCATTTAGATGCTCCCAATTGGGCTTGCCTCTATTTTGCTCCTC
EMSA probe1	5’biotin-TGTGCTAGATTCATCGCTCT-3’
EMSA probe2	5’biotin-AGCTCCATTGTTTCTGCAAA-3’
