# Supplementary material for: Exosomal circSPIRE1 mediates glycosylation of E-cadherin to suppress metastasis of renal cell carcinoma
Source: Oncogene. 2023 Apr 12;42(22):1802–20. doi: 10.1038/s41388-023-02678-7 (PMC10238271; doi:10.1038/s41388-023-02678-7)
Supplement: Supplementary file 7 — supplementary table 5 [file 41388_2023_2678_MOESM7_ESM.pdf]

Supplementary table 5 contact distance model 1

name	distance	category	type	from	from chemistry	to	to chemistry
B:GLY243:N - A:C159:OP1	3.60716	rogen Bond;Electros	Salt Bridge	B:GLY243:N	H-Donor	A:C159:OP	H-Acceptor
B:GLY243:N - A:G158:OP1	5.42418	Electrostatic	Attractive Charge	B:GLY243:N	Positive	A:G158:OP	Negative
B:LYS274:NZ - A:A161:OP2	4.88661	Electrostatic	Attractive Charge	B:LYS274:NZ	Positive	A:A161:OP	Negative
A:G84:N1 - B:VAL317:O	2.65499	Hydrogen Bond	Conventional Hydrogen Bond	A:G84:N1	H-Donor	B:VAL317:	H-Acceptor
A:A86:N6 - B:SER318:OG	2.37375	Hydrogen Bond	Conventional Hydrogen Bond	A:A86:N6	H-Donor	:SER318:O	H-Acceptor
A:A157:N6 - B:THR321:OXT	2.37635	Hydrogen Bond	Conventional Hydrogen Bond	A:A157:N6	H-Donor	THR321:O	H-Acceptor
B:TYR295:OH - A:A157:OP1	2.52934	Hydrogen Bond	Conventional Hydrogen Bond	B:TYR295:OH	H-Donor	A:A157:OP	H-Acceptor
B:THR321:N - A:G87:O6	2.26415	Hydrogen Bond	Conventional Hydrogen Bond	B:THR321:N	H-Donor	A:G87:O6	H-Acceptor
A:G158:C5' - B:GLY243:O	2.65942	Hydrogen Bond	Carbon Hydrogen Bond	A:G158:C5'	H-Donor	B:GLY243:	H-Acceptor
B:GLN262:CA - A:G73:OP2	3.54324	Hydrogen Bond	Carbon Hydrogen Bond	B:GLN262:CA	H-Donor	A:G73:OP2	H-Acceptor
B:LYS285:NZ - A:A161	4.75344	Electrostatic	Pi-Cation	B:LYS285:NZ	Positive	A:A161	Pi-Orbitals
B:LYS285:NZ - A:A161	3.90313	rogen Bond;Electros	-Cation;Pi-Donor Hydrogen Bo	B:LYS285:NZ	Positive;H-Donor	A:A161	Pi-Orbitals;Pi-Orbitals
B:LYS320:NZ - A:C88	4.30361	Electrostatic	Pi-Cation	B:LYS320:NZ	Positive	A:C88	Pi-Orbitals
B:LYS320:NZ - A:G160	4.71427	Electrostatic	Pi-Cation	B:LYS320:NZ	Positive	A:G160	Pi-Orbitals
B:LYS320:NZ - A:G160	4.4788	Electrostatic	Pi-Cation	B:LYS320:NZ	Positive	A:G160	Pi-Orbitals
A:A157:OP1 - B:PHE319	3.33625	Electrostatic	Pi-Anion	A:A157:OP1	Negative	B:PHE319	Pi-Orbitals
A:G160:OP2 - B:PHE289	4.80623	Electrostatic	Pi-Anion	A:G160:OP2	Negative	B:PHE289	Pi-Orbitals
B:CYS245:SG - A:C159	3.54139	Hydrogen Bond	Pi-Donor Hydrogen Bond	B:CYS245:SG	H-Donor	A:C159	Pi-Orbitals
B:ASN306:ND2 - A:G84	2.42205	Hydrogen Bond	Pi-Donor Hydrogen Bond	B:ASN306:ND2	H-Donor	A:G84	Pi-Orbitals
B:ILE276:CG2 - A:A161	3.39782	Hydrophobic	Pi-Sigma	B:ILE276:CG2	C-H	A:A161	Pi-Orbitals
A:A86:N3 - B:PHE247	2.88758	Other	Pi-Lone Pair	A:A86:N3	Lone Pair	B:PHE247	Pi-Orbitals
A:A82 - B:ALA303	4.5863	Hydrophobic	Pi-Alkyl	A:A82	Pi-Orbitals	B:ALA303	Alkyl
A:G87 - B:LYS320	4.71271	Hydrophobic	Pi-Alkyl	A:G87	Pi-Orbitals	B:LYS320	Alkyl
A:G158 - B:LYS320	5.09086	Hydrophobic	Pi-Alkyl	A:G158	Pi-Orbitals	B:LYS320	Alkyl
A:G158 - B:LYS320	5.00377	Hydrophobic	Pi-Alkyl	A:G158	Pi-Orbitals	B:LYS320	Alkyl
A:A161 - B:ILE276	4.89121	Hydrophobic	Pi-Alkyl	A:A161	Pi-Orbitals	B:ILE276	Alkyl
A:U162 - B:ILE276	5.30214	Hydrophobic	Pi-Alkyl	A:U162	Pi-Orbitals	B:ILE276	Alkyl
A:U162 - B:LYS285	3.85278	Hydrophobic	Pi-Alkyl	A:U162	Pi-Orbitals	B:LYS285	Alkyl
A:G163 - B:LYS285	3.91336	Hydrophobic	Pi-Alkyl	A:G163	Pi-Orbitals	B:LYS285	Alkyl
A:G163 - B:LYS285	3.96849	Hydrophobic	Pi-Alkyl	A:G163	Pi-Orbitals	B:LYS285	Alkyl

Supplementary table 5 contact distance model 2

name	distance	category	type	from	from chemistry	to	to chemistry
B:LYS285:NZ - A:U168:OP1	3.0357	Hydrogen Bond;Electrostatic	Salt Bridge	B:LYS285:NZ	H-Donor	A:U168:OP1	H-Acceptor
B:ARG309:NH1 - A:A190:OP2	3.09195	Hydrogen Bond;Electrostatic	Salt Bridge	B:ARG309:NH1	H-Donor	A:A190:OP2	H-Acceptor
B:ARG309:NH2 - A:A190:OP1	3.90304	Hydrogen Bond;Electrostatic	Salt Bridge	B:ARG309:NH2	H-Donor	A:A190:OP1	H-Acceptor
B:LYS274:NZ - A:U166:OP2	4.41594	Electrostatic	Attractive Charge	B:LYS274:NZ	Positive	A:U166:OP2	Negative
B:LYS285:NZ - A:A169:OP2	3.92839	Electrostatic	Attractive Charge	B:LYS285:NZ	Positive	A:A169:OP2	Negative
B:ARG309:NH1 - A:C201:OP1	4.18554	Electrostatic	Attractive Charge	B:ARG309:NH1	Positive	A:C201:OP1	Negative
A:A169:N6 - B:ILE248:O	2.72504	Hydrogen Bond	Conventional Hydrogen Bond	A:A169:N6	H-Donor	B:ILE248:O	H-Acceptor
A:A169:N6 - B:GLY286:O	2.73454	Hydrogen Bond	Conventional Hydrogen Bond	A:A169:N6	H-Donor	B:GLY286:O	H-Acceptor
A:G186:O2' - B:ASP254:OD2	3.19749	Hydrogen Bond	Conventional Hydrogen Bond	A:G186:O2'	H-Donor	B:ASP254:OD2	H-Acceptor
B:GLN253:N - A:G186:OP1	2.6796	Hydrogen Bond	Conventional Hydrogen Bond	B:GLN253:N	H-Donor	A:G186:OP1	H-Acceptor
B:ASN272:ND2 - A:U166:O4	2.48945	Hydrogen Bond	Conventional Hydrogen Bond	B:ASN272:ND2	H-Donor	A:U166:O4	H-Acceptor
B:THR291:OG1 - A:U166:O4	2.84086	Hydrogen Bond	Conventional Hydrogen Bond	B:THR291:OG1	H-Donor	A:U166:O4	H-Acceptor
A:A169:C2 - B:GLN316:OE1	3.57253	Hydrogen Bond	Carbon Hydrogen Bond	A:A169:C2	H-Donor	B:GLN316:OE1	H-Acceptor
A:G186:C4' - B:ASP254:OD2	2.64253	Hydrogen Bond	Carbon Hydrogen Bond	A:G186:C4'	H-Donor	B:ASP254:OD2	H-Acceptor
B:GLY243:CA - A:G163:O6	3.16462	Hydrogen Bond	Carbon Hydrogen Bond	B:GLY243:CA	H-Donor	A:G163:O6	H-Acceptor
B:THR271:CG2 - A:G163	3.49288	Hydrophobic	Pi-Sigma	B:THR271:CG2	C-H	A:G163	Pi-Orbitals
B:THR293:CG2 - A:U162	3.24568	Hydrophobic	Pi-Sigma	B:THR293:CG2	C-H	A:U162	Pi-Orbitals
A:C167:O2 - B:PHE289	2.74556	Other	Pi-Lone Pair	A:C167:O2	Lone Pair	B:PHE289	Pi-Orbitals
A:A169:N3 - B:TYR249	2.77692	Other	Pi-Lone Pair	A:A169:N3	Lone Pair	B:TYR249	Pi-Orbitals
A:U168 - B:PHE247	5.07112	Hydrophobic	Pi-Pi T-shaped	A:U168	Pi-Orbitals	B:PHE247	Pi-Orbitals
A:A165 - B:CYS245	5.39333	Hydrophobic	Pi-Alkyl	A:A165	Pi-Orbitals	B:CYS245	Alkyl
A:U168 - B:ILE276	4.93166	Hydrophobic	Pi-Alkyl	A:U168	Pi-Orbitals	B:ILE276	Alkyl

Supplementary table 5 contact distance model 3

name	distance	category	type	from	om chemist	to	to chemistry
B:LYS274:NZ - A:A157:OP1	2.93787	drogen Bond;Electrost:	Salt Bridge	B:LYS274:NZ	H-Donor	A:A157:OP1	H-Acceptor
B:LYS283:NZ - A:A79:OP2	3.06376	drogen Bond;Electrost:	Salt Bridge	B:LYS283:NZ	H-Donor	A:A79:OP2	H-Acceptor
B:LYS274:NZ - A:G158:OP2	3.25167	Electrostatic	Attractive Charge	B:LYS274:NZ	Positive	A:G158:OP2	Negative
B:ARG309:NH2 - A:U70:OP2	5.04923	Electrostatic	Attractive Charge	B:ARG309:NH2	Positive	A:U70:OP2	Negative
A:C74:N4 - B:ILE314:O	2.62382	Hydrogen Bond	Conventional Hydrogen Bond	A:C74:N4	H-Donor	B:ILE314:O	H-Acceptor
A:C74:N4 - B:LEU315:O	3.37248	Hydrogen Bond	Conventional Hydrogen Bond	A:C74:N4	H-Donor	B:LEU315:O	H-Acceptor
A:C80:N4 - B:ASP254:O	2.77293	Hydrogen Bond	Conventional Hydrogen Bond	A:C80:N4	H-Donor	B:ASP254:O	H-Acceptor
A:A82:N6 - B:ASP256:OD1	2.9685	Hydrogen Bond	Conventional Hydrogen Bond	A:A82:N6	H-Donor	B:ASP256:OD1	H-Acceptor
A:G84:N2 - B:VAL270:O	2.96322	Hydrogen Bond	Conventional Hydrogen Bond	A:G84:N2	H-Donor	B:VAL270:O	H-Acceptor
B:TYR249:OH - A:G76:OP2	3.20333	Hydrogen Bond	Conventional Hydrogen Bond	B:TYR249:OH	H-Donor	A:G76:OP2	H-Acceptor
B:GLN253:N - A:A78:N7	3.22758	Hydrogen Bond	Conventional Hydrogen Bond	B:GLN253:N	H-Donor	A:A78:N7	H-Acceptor
B:GLN253:NE2 - A:A77:N7	3.30678	Hydrogen Bond	Conventional Hydrogen Bond	B:GLN253:NE2	H-Donor	A:A77:N7	H-Acceptor
B:ASN306:N - A:U72:OP2	2.75998	Hydrogen Bond	Conventional Hydrogen Bond	B:ASN306:N	H-Donor	A:U72:OP2	H-Acceptor
B:VAL317:N - A:G73:OP2	3.07088	Hydrogen Bond	Conventional Hydrogen Bond	B:VAL317:N	H-Donor	A:G73:OP2	H-Acceptor
A:U72:C6 - B:ASN306:O	3.50852	Hydrogen Bond	Carbon Hydrogen Bond	A:U72:C6	H-Donor	B:ASN306:O	H-Acceptor
A:G73:C8 - B:ASN306:OD1	3.49349	Hydrogen Bond	Carbon Hydrogen Bond	A:G73:C8	H-Donor	B:ASN306:OD1	H-Acceptor
A:C74:C3' - B:GLN316:OE1	2.77163	Hydrogen Bond	Carbon Hydrogen Bond	A:C74:C3'	H-Donor	B:GLN316:OE1	H-Acceptor
A:A75:C3' - B:TYR249:OH	3.39983	Hydrogen Bond	Carbon Hydrogen Bond	A:A75:C3'	H-Donor	B:TYR249:OH	H-Acceptor
A:A78:C2' - B:ASP254:OD2	3.53634	Hydrogen Bond	Carbon Hydrogen Bond	A:A78:C2'	H-Donor	B:ASP254:OD2	H-Acceptor
A:A75:OP2 - B:TYR249	2.68564	Electrostatic	Pi-Anion	A:A75:OP2	Negative	B:TYR249	Pi-Orbitals
B:ASP254:OD2 - A:A78	4.53292	Electrostatic	Pi-Anion	B:ASP254:OD2	Negative	A:A78	Pi-Orbitals
A:G84:N1 - B:TRP261	2.31483	Hydrogen Bond	Pi-Donor Hydrogen Bond	A:G84:N1	H-Donor	B:TRP261	Pi-Orbitals
A:G84:O6 - B:TRP261	2.77231	Other	Pi-Lone Pair	A:G84:O6	Lone Pair	B:TRP261	Pi-Orbitals
A:G84:O6 - B:TRP261	2.83218	Other	Pi-Lone Pair	A:G84:O6	Lone Pair	B:TRP261	Pi-Orbitals
A:G84 - B:TRP261	3.60738	Hydrophobic	Pi-Pi T-shaped	A:G84	Pi-Orbitals	B:TRP261	Pi-Orbitals
A:G76 - B:LYS313	5.39091	Hydrophobic	Pi-Alkyl	A:G76	Pi-Orbitals	B:LYS313	Alkyl
A:G76 - B:LYS313	4.87306	Hydrophobic	Pi-Alkyl	A:G76	Pi-Orbitals	B:LYS313	Alkyl
A:G196 - B:ILE314	4.40266	Hydrophobic	Pi-Alkyl	A:G196	Pi-Orbitals	B:ILE314	Alkyl
A:G196 - B:ILE314	4.27827	Hydrophobic	Pi-Alkyl	A:G196	Pi-Orbitals	B:ILE314	Alkyl
A:C197 - B:ILE314	5.16168	Hydrophobic	Pi-Alkyl	A:C197	Pi-Orbitals	B:ILE314	Alkyl
