# Supplementary material for: Exosomal circSPIRE1 mediates glycosylation of E-cadherin to suppress metastasis of renal cell carcinoma
Source: Oncogene. 2023 Apr 12;42(22):1802–20. doi: 10.1038/s41388-023-02678-7 (PMC10238271; doi:10.1038/s41388-023-02678-7)
Supplement: Supplementary file 8 — supplementary table 6 [file 41388_2023_2678_MOESM8_ESM.pdf]

Table S6. NCG result of PDX LM p-CTRL cells versus PDX LM p-circSPIRE1 cells

mRNA	RNA_type	Gene	Gene_type	OE1	OE2	OE3	NC1	NC2	NC3	log2(Fold_change)	p-value	Website
NM_001195812.1	mRNA	PLD5	protein_coding	3.743753823	3.270730274	4.054835025	6.94E-17	6.94E-17	6.94E-17	-4.935799663	0.001221876	http://www.ncbi.nlm.nih.gov/nuccore/NM_001195812.1
NM_001195811.1	mRNA	PLD5	protein_coding	3.743753823	3.270730274	4.054835025	6.94E-17	6.94E-17	6.94E-17	-4.935799454	0.00122205	http://www.ncbi.nlm.nih.gov/nuccore/NM_001195811.1
NM_001085375.1	mRNA	C1orf226	protein_coding	4.723325102	4.299473959	1.027494921	6.94E-17	6.94E-17	6.94E-17	-4.802284525	0.002660076	http://www.ncbi.nlm.nih.gov/nuccore/NM_001085375.1
NM_001278723.1	mRNA	IL20RA	protein_coding	0	3.24620749	6.062586495	6.94E-17	6.94E-17	6.94E-17	-4.668222508	0.007099621	http://www.ncbi.nlm.nih.gov/nuccore/NM_001278723.1
NM_001278724.1	mRNA	IL20RA	protein_coding	0	3.24620749	6.062586495	6.94E-17	6.94E-17	6.94E-17	-4.668217109	0.007097164	http://www.ncbi.nlm.nih.gov/nuccore/NM_001278724.1
NM_014432.3	mRNA	IL20RA	protein_coding	0	3.24620749	6.062586495	6.94E-17	6.94E-17	6.94E-17	-4.668210778	0.007094286	http://www.ncbi.nlm.nih.gov/nuccore/NM_014432.3
NM_004497.2	mRNA	FOXA3	protein_coding	1.835356222	2.201912925	5.05651917	6.94E-17	6.94E-17	6.94E-17	-4.657953254	0.004958065	http://www.ncbi.nlm.nih.gov/nuccore/NM_004497.2
NM_003658.4	mRNA	BARX2	protein_coding	2.798578164	3.246146293	3.043125353	6.94E-17	6.94E-17	6.94E-17	-4.657471817	0.004609956	http://www.ncbi.nlm.nih.gov/nuccore/NM_003658.4
NM_005538.3	mRNA	INHBC	protein_coding	4.735599753	2.201859461	2.035312278	6.94E-17	6.94E-17	6.94E-17	-4.653837369	0.004872271	http://www.ncbi.nlm.nih.gov/nuccore/NM_005538.3
NM_178013.3	mRNA	PRIMA1	protein_coding	5.707215902	0.057620021	3.043106487	6.94E-17	6.94E-17	6.94E-17	-4.64168555	0.006806841	http://www.ncbi.nlm.nih.gov/nuccore/NM_178013.3
NM_001278722.1	mRNA	IL20RA	protein_coding	0	3.233225529	5.053791789	6.94E-17	6.94E-17	6.94E-17	-4.503292066	0.011319699	http://www.ncbi.nlm.nih.gov/nuccore/NM_001278722.1
NM_001288587.2	mRNA	FAM83A	protein_coding	0.887034681	1.13934413	6.059634009	6.94E-17	6.94E-17	6.94E-17	-4.49659004	0.012486999	http://www.ncbi.nlm.nih.gov/nuccore/NM_001288587.2
NM_018931.2	mRNA	PCDHB11	protein_coding	1.844473599	2.190582472	4.047575171	6.94E-17	6.94E-17	6.94E-17	-4.494930092	0.009234585	http://www.ncbi.nlm.nih.gov/nuccore/NM_018931.2
NM_002521.2	mRNA	NPPB	protein_coding	2.809075058	3.233168848	2.03335	6.94E-17	6.94E-17	6.94E-17	-4.494592515	0.009038353	http://www.ncbi.nlm.nih.gov/nuccore/NM_002521.2
NM_001160213.1	mRNA	PMFBP1	protein_coding	0.89472573	3.219681246	3.038507945	6.94E-17	6.94E-17	6.94E-17	-4.314068957	0.018077453	http://www.ncbi.nlm.nih.gov/nuccore/NM_001160213.1
NM_201589.3	mRNA	MAFA	protein_coding	1.853963789	2.178811603	3.038502592	6.94E-17	6.94E-17	6.94E-17	-4.311237482	0.017657439	http://www.ncbi.nlm.nih.gov/nuccore/NM_201589.3
NM_001031680.2	mRNA	RUNX3	protein_coding	1.853973141	1.129737418	4.044944931	6.94E-17	6.94E-17	6.94E-17	-4.310045545	0.018429772	http://www.ncbi.nlm.nih.gov/nuccore/NM_001031680.2
NM_005293.2	mRNA	GPR20	protein_coding	3.789738356	3.219629335	0.008028351	6.94E-17	6.94E-17	6.94E-17	-4.3086897	0.019892832	http://www.ncbi.nlm.nih.gov/nuccore/NM_005293.2
NM_001024209.3	mRNA	SPRR2E	protein_coding	7.472427805	4.459444833	12.14074494	1.039723058	0.001338274	0	-4.168310417	5.04E-06	http://www.ncbi.nlm.nih.gov/nuccore/NM_001024209.3
NM_001317944.1	mRNA	BSPRY	protein_coding	0.902714432	4.240540348	1.020921433	6.94E-17	6.94E-17	6.94E-17	-4.107305343	0.036989949	http://www.ncbi.nlm.nih.gov/nuccore/NM_001317944.1
NM_003593.2	mRNA	FOXN1	protein_coding	0.902714432	4.240540348	1.020921433	6.94E-17	6.94E-17	6.94E-17	-4.107302105	0.036986786	http://www.ncbi.nlm.nih.gov/nuccore/NM_003593.2
NM_001110219.2	mRNA	GJB6	protein_coding	1.863882136	4.240522951	0.006899718	6.94E-17	6.94E-17	6.94E-17	-4.106071052	0.038884827	http://www.ncbi.nlm.nih.gov/nuccore/NM_001110219.2
NM_001110220.2	mRNA	GJB6	protein_coding	1.863882136	4.240522951	0.006899718	6.94E-17	6.94E-17	6.94E-17	-4.106067437	0.038878875	http://www.ncbi.nlm.nih.gov/nuccore/NM_001110220.2
NM_001136127.2	mRNA	DNM3	protein_coding	0.902721193	3.205511683	2.029162796	6.94E-17	6.94E-17	6.94E-17	-4.103911392	0.035077494	http://www.ncbi.nlm.nih.gov/nuccore/NM_001136127.2
NM_015569.4	mRNA	DNM3	protein_coding	0.902721193	3.205511683	2.029162796	6.94E-17	6.94E-17	6.94E-17	-4.103909181	0.035074785	http://www.ncbi.nlm.nih.gov/nuccore/NM_015569.4
NM_030820.3	mRNA	COL21A1	protein_coding	0.902727951	2.166532134	3.036028815	6.94E-17	6.94E-17	6.94E-17	-4.10252958	0.034985577	http://www.ncbi.nlm.nih.gov/nuccore/NM_030820.3
NM_001318752.1	mRNA	COL21A1	protein_coding	0.902727951	2.166532134	3.036028815	6.94E-17	6.94E-17	6.94E-17	-4.102528718	0.034983866	http://www.ncbi.nlm.nih.gov/nuccore/NM_001318752.1
NM_001318751.1	mRNA	COL21A1	protein_coding	0.902727951	2.166532134	3.036028815	6.94E-17	6.94E-17	6.94E-17	-4.102528246	0.03498293	http://www.ncbi.nlm.nih.gov/nuccore/NM_001318751.1
NM_194448.2	mRNA	CLEC4A	protein_coding	0.902727951	2.166532134	3.036028815	6.94E-17	6.94E-17	6.94E-17	-4.102527948	0.03498234	http://www.ncbi.nlm.nih.gov/nuccore/NM_194448.2
NM_002458.2	mRNA	MUC5B	protein_coding	0.902727951	2.166532134	3.036028815	6.94E-17	6.94E-17	6.94E-17	-4.102527534	0.034981517	http://www.ncbi.nlm.nih.gov/nuccore/NM_002458.2
NM_024306.4	mRNA	FA2H	protein_coding	1.863890576	3.205496055	1.020918074	6.94E-17	6.94E-17	6.94E-17	-4.102174815	0.035023025	http://www.ncbi.nlm.nih.gov/nuccore/NM_024306.4
NM_005992.1	mRNA	TBX1	protein_coding	2.83156473	3.205480432	0.006897557	6.94E-17	6.94E-17	6.94E-17	-4.100529237	0.037176459	http://www.ncbi.nlm.nih.gov/nuccore/NM_005992.1
NM_004350.2	mRNA	RUNX3	protein_coding	1.863907452	1.119794267	3.036023977	6.94E-17	6.94E-17	6.94E-17	-4.099552318	0.034971387	http://www.ncbi.nlm.nih.gov/nuccore/NM_004350.2
NM_001320672.1	mRNA	RUNX3	protein_coding	1.863907452	1.119794267	3.036023977	6.94E-17	6.94E-17	6.94E-17	-4.099552178	0.034972054	http://www.ncbi.nlm.nih.gov/nuccore/NM_001320672.1
NM_178342.2	mRNA	C3orf35	protein_coding	3.802634206	2.166491536	0.006895398	6.94E-17	6.94E-17	6.94E-17	-4.09532607	0.03844515	http://www.ncbi.nlm.nih.gov/nuccore/NM_178342.2
NM_001142966.1	mRNA	GREB1L	protein_coding	3.713262241	5.392687848	5.068826955	0.004261051	0.001337827	0.92767323	-3.411483971	0.001290931	http://www.ncbi.nlm.nih.gov/nuccore/NM_001142966.1
NM_001017363.1	mRNA	ARID3C	protein_coding	2.751181124	5.392720073	6.07577852	1.039723058	0.001338274	0	-3.410858672	0.001353829	http://www.ncbi.nlm.nih.gov/nuccore/NM_001017363.1
NM_003294.3	mRNA	TPSAB1	protein_coding	2.760117999	4.338446226	6.073299214	0.004261051	0.001337827	0.92767323	-3.307953136	0.002432645	http://www.ncbi.nlm.nih.gov/nuccore/NM_003294.3
NM_005185.3	mRNA	CALML3	protein_coding	1.809726502	4.325887824	6.070756366	1.039723058	0.001338274	0	-3.195067669	0.004611418	http://www.ncbi.nlm.nih.gov/nuccore/NM_005185.3
NM_004750.4	mRNA	CRLF1	protein_coding	3.733309538	2.233775472	6.070736923	0.00426215	1.012321538	0	-3.191524661	0.00453314	http://www.ncbi.nlm.nih.gov/nuccore/NM_004750.4
NM_178348.2	mRNA	LCE1A	protein_coding	4.700170072	3.282441011	4.057087798	1.039723058	0.001338274	0	-3.191318667	0.004239406	http://www.ncbi.nlm.nih.gov/nuccore/NM_178348.2
NM_002699.3	mRNA	POU3F1	protein_coding	0.865170805	6.388392343	4.054858787	0.00426215	1.012321538	0	-3.079511746	0.009234994	http://www.ncbi.nlm.nih.gov/nuccore/NM_002699.3
NM_152666.2	mRNA	PLD5	protein_coding	3.743753823	3.270730274	4.054835025	1.039723058	0.001338274	0	-3.0704466	0.007622071	http://www.ncbi.nlm.nih.gov/nuccore/NM_152666.2
NM_001320272.1	mRNA	PLD5	protein_coding	3.743753823	3.270730274	4.054835025	1.039723058	0.001338274	0	-3.070446143	0.007622628	http://www.ncbi.nlm.nih.gov/nuccore/NM_001320272.1
NM_000518.4	mRNA	HBB	protein_coding	6.053714948	24.04565194	63.67307597	0.045935175	10.10644465	0.8025434	-3.05468183	0.000880738	http://www.ncbi.nlm.nih.gov/nuccore/NM_000518.4
NM_018690.3	mRNA	APOBR	protein_coding	7.625036322	2.223365159	1.029014063	1.039723058	0.001338274	0	-3.053781914	0.012193842	http://www.ncbi.nlm.nih.gov/nuccore/NM_018690.3
NM_015464.2	mRNA	SOSTDC1	protein_coding	2.788469477	4.299522258	3.045307162	0.00426215	1.012321538	0	-2.939633304	0.013772661	http://www.ncbi.nlm.nih.gov/nuccore/NM_015464.2
NM_001276331.1	mRNA	LCE1C	protein_coding	1.826557117	2.212865093	6.06539727	0.00426215	1.012321538	0	-2.939215254	0.015263013	http://www.ncbi.nlm.nih.gov/nuccore/NM_001276331.1
NM_024875.4	mRNA	SYNPO2L	protein_coding	9.488555723	4.385462424	3.058862382	0.008502595	0.002669375	1.868557097	-2.89282743	0.001415321	http://www.ncbi.nlm.nih.gov/nuccore/NM_024875.4
NM_001114133.2	mRNA	SYNPO2L	protein_coding	9.488555723	4.385462424	3.058862382	0.008502595	0.002669375	1.868557097	-2.892827384	0.001415341	http://www.ncbi.nlm.nih.gov/nuccore/NM_001114133.2
NM_001321839.1	mRNA	CA8	protein_coding	3.694273402	7.498549737	5.073277532	0.008504778	1.015157451	0.910876161	-2.813720439	0.001843034	http://www.ncbi.nlm.nih.gov/nuccore/NM_001321839.1
NM_024575.4	mRNA	TNFAIP8L2	protein_coding	1.835345168	3.246166686	4.050093054	0.00426215	1.012321538	0	-2.79358394	0.024766547	http://www.ncbi.nlm.nih.gov/nuccore/NM_024575.4
NM_003248.5	mRNA	THBS4	protein_coding	4.735584336	3.246105524	1.025922021	1.039723058	0.001338274	0	-2.788787298	0.026035722	http://www.ncbi.nlm.nih.gov/nuccore/NM_003248.5
NM_001306214.1	mRNA	THBS4	protein_coding	4.735584336	3.246105524	1.025922021	1.039723058	0.001338274	0	-2.788784937	0.026038992	http://www.ncbi.nlm.nih.gov/nuccore/NM_001306214.1
NM_001319977.1	mRNA	CASC1	protein_coding	3.765844996	0.057640138	5.056503964	0.004261051	0.001337827	0.92767323	-2.788392661	0.029757376	http://www.ncbi.nlm.nih.gov/nuccore/NM_001319977.1
NM_001204101.2	mRNA	CASC1	protein_coding	3.765844996	0.057640138	5.056503964	0.004261051	0.001337827	0.92767323	-2.788390435	0.029764065	http://www.ncbi.nlm.nih.gov/nuccore/NM_001204101.2
NM_032330.1	mRNA	CAPNS2	protein_coding	1.844442924	5.306585856	1.024317706	0.00426215	1.012321538	0	-2.634426673	0.047463311	http://www.ncbi.nlm.nih.gov/nuccore/NM_032330.1
NM_178827.4	mRNA	IQUB	protein_coding	1.844442924	5.306585856	1.024317706	0.00426215	1.012321538	0	-2.634424447	0.047458531	http://www.ncbi.nlm.nih.gov/nuccore/NM_178827.4
NM_014395.2	mRNA	DAPP1	protein_coding	1.844453151	4.271300215	2.033355074	0.004261051	0.001337827	0.92767323	-2.633912227	0.044373891	http://www.ncbi.nlm.nih.gov/nuccore/NM_014395.2
NM_001306151.1	mRNA	DAPP1	protein_coding	1.844453151	4.271300215	2.033355074	0.004261051	0.001337827	0.92767323	-2.633910009	0.04437085	http://www.ncbi.nlm.nih.gov/nuccore/NM_001306151.1
NM_001317943.1	mRNA	BSPRY	protein_coding	1.844442924	5.306585856	1.024317706	1.039723058	0.001338274	0	-2.633466598	0.047432613	http://www.ncbi.nlm.nih.gov/nuccore/NM_001317943.1
NM_017688.2	mRNA	BSPRY	protein_coding	1.844442924	5.306585856	1.024317706	1.039723058	0.001338274	0	-2.633464438	0.047426576	http://www.ncbi.nlm.nih.gov/nuccore/NM_017688.2
NM_194450.2	mRNA	CLEC4A	protein_coding	0.887026343	2.19059894	5.053784716	1.039723058	0.001338274	0	-2.630766898	0.046846804	http://www.ncbi.nlm.nih.gov/nuccore/NM_194450.2
NM_004626.2	mRNA	WNT11	protein_coding	1.844483819	1.139330663	5.053777646	1.039723058	0.001338274	0	-2.627509631	0.046767159	http://www.ncbi.nlm.nih.gov/nuccore/NM_004626.2

NM_020980.4	mRNA	AQP9	protein_coding	3.777524617	1.13930374	3.040854131	1.039723058	0.001338274	0	-2.62536151	0.044612705	http://www.ncbi.nlm.nih.gov/nuccore/NM_020980.4
NM_000517.4	mRNA	HBA2	protein_coding	1.71611102	5.520858676	18.18112251	0.016936353	2.028546503	1.831392452	-2.583496968	0.004872077	http://www.ncbi.nlm.nih.gov/nuccore/NM_000517.4
NM_033378.1	mRNA	CGB2	protein_coding	0.858250454	5.365881683	6.070766095	1.048840068	1.015159234	0	-2.410546826	0.017702901	http://www.ncbi.nlm.nih.gov/nuccore/NM_033378.1
NM_001243126.1	mRNA	KLK7	protein_coding	6.580041247	6.472451672	4.067489385	3.103558749	0.003998667	0	-2.387144017	0.003635535	http://www.ncbi.nlm.nih.gov/nuccore/NM_001243126.1
NM_005046.3	mRNA	KLK7	protein_coding	9.427766962	6.534077516	6.093509901	4.135541953	0.005320845	0	-2.375452436	0.000934853	http://www.ncbi.nlm.nih.gov/nuccore/NM_005046.3
NM_001204744.1	mRNA	CDH16	protein_coding	17.45729693	40.82451248	37.48547365	8.38001838	6.101528338	4.454116939	-2.321508138	9.05E-09	http://www.ncbi.nlm.nih.gov/nuccore/NM_001204744.1
NM_001204745.1	mRNA	CDH16	protein_coding	17.45729693	40.82451248	37.48547365	8.38001838	6.101528338	4.454116939	-2.321508135	9.04E-09	http://www.ncbi.nlm.nih.gov/nuccore/NM_001204745.1
NM_000395.2	mRNA	CSF2RB	protein_coding	6.591395736	2.272654318	7.087378996	1.057017353	1.017698196	0.895945772	-2.300530439	0.006255084	http://www.ncbi.nlm.nih.gov/nuccore/NM_000395.2
NM_001278423.1	mRNA	PKD1L2	protein_coding	0.865181343	5.351824937	5.061701709	2.071685456	0.002671153	0	-2.288362351	0.029524935	http://www.ncbi.nlm.nih.gov/nuccore/NM_001278423.1
NM_001307985.1	mRNA	SLC22A11	protein_coding	2.778751636	2.223467066	6.068112919	1.048840068	1.015159234	0	-2.282923435	0.027328942	http://www.ncbi.nlm.nih.gov/nuccore/NM_001307985.1
NM_018484.3	mRNA	SLC22A11	protein_coding	2.778751636	2.223467066	6.068112919	1.048840068	1.015159234	0	-2.282923218	0.027329592	http://www.ncbi.nlm.nih.gov/nuccore/NM_018484.3
NM_198207.2	mRNA	CERS1	protein_coding	4.711554419	3.270707078	3.047401838	1.048840068	1.015159234	0	-2.282283827	0.02602023	http://www.ncbi.nlm.nih.gov/nuccore/NM_198207.2
NM_198501.2	mRNA	SMTNL2	protein_coding	12.15662096	11.94498781	14.18816419	2.124913684	4.05540083	1.771348614	-2.22215551	2.12E-05	http://www.ncbi.nlm.nih.gov/nuccore/NM_198501.2
NM_001302454.1	mRNA	CCDC151	protein_coding	2.742464913	6.44597814	6.078191204	0.012734538	3.032194864	0	-2.215369151	0.010376155	http://www.ncbi.nlm.nih.gov/nuccore/NM_001302454.1
NM_001306213.1	mRNA	THBS4	protein_coding	5.634531073	7.484198075	2.045987652	2.08223591	1.017700628	0	-2.211992455	0.010653852	http://www.ncbi.nlm.nih.gov/nuccore/NM_001306213.1
NM_139277.2	mRNA	KLK7	protein_coding	6.558319743	6.497900325	6.087227099	4.135541953	0.005320845	0	-2.169970663	0.003768059	http://www.ncbi.nlm.nih.gov/nuccore/NM_139277.2
NM_000442.4	mRNA	PECAM1	protein_coding	3.754574446	3.258632895	3.045300431	0.008502595	0.002669375	1.868557097	-2.153580221	0.04386905	http://www.ncbi.nlm.nih.gov/nuccore/NM_000442.4
NM_001243007.1	mRNA	PROX2	protein_coding	1.82653341	4.299546418	4.0525142	0.008506963	2.022272853	0	-2.15350506	0.04479339	http://www.ncbi.nlm.nih.gov/nuccore/NM_001243007.1
NM_001290265.1	mRNA	CERS1	protein_coding	4.723357966	2.212807701	3.045293703	1.048840068	1.015159234	0	-2.148193357	0.044089111	http://www.ncbi.nlm.nih.gov/nuccore/NM_001290265.1
NM_001195430.1	mRNA	DCLK1	protein_coding	5.694035226	1.157611948	3.045286978	0.008504778	1.015157451	0.910876161	-2.146973474	0.046896452	http://www.ncbi.nlm.nih.gov/nuccore/NM_001195430.1
NM_001178134.1	mRNA	CXCL12	protein_coding	4.678444222	3.304970774	6.075757224	1.057017353	1.017698196	0.895945772	-2.115213398	0.015432452	http://www.ncbi.nlm.nih.gov/nuccore/NM_001178134.1
NM_001216.2	mRNA	CA9	protein_coding	5.602762746	5.442494523	7.092202004	2.091864763	2.028554161	0	-2.094645425	0.00569736	http://www.ncbi.nlm.nih.gov/nuccore/NM_001216.2
NM_001282424.2	mRNA	A2ML1	protein_coding	5.563428416	9.6595293	7.101226041	4.14716039	1.02224429	0	-2.083380245	0.002448269	http://www.ncbi.nlm.nih.gov/nuccore/NM_001282424.2
NM_001114974.1	mRNA	SMTNL2	protein_coding	16.85101792	12.08593609	22.27598211	2.15307652	8.096763676	1.720668509	-2.075921778	8.43E-06	http://www.ncbi.nlm.nih.gov/nuccore/NM_001114974.1
NM_153370.2	mRNA	PI16	protein_coding	14.27927632	4.438968215	3.067341926	0.021127667	3.038921199	1.815060514	-2.065959325	0.008092748	http://www.ncbi.nlm.nih.gov/nuccore/NM_153370.2
NM_001286447.1	mRNA	FAM65B	protein_coding	11.23194369	8.761820075	14.17898389	1.091104511	4.055392971	2.718959887	-2.063353524	0.000149537	http://www.ncbi.nlm.nih.gov/nuccore/NM_001286447.1
NM_001286446.2	mRNA	FAM65B	protein_coding	11.23194369	8.761820075	14.17898389	1.091104511	4.055392971	2.718959887	-2.063353331	0.000149592	http://www.ncbi.nlm.nih.gov/nuccore/NM_001286446.2
NM_001289129.1	mRNA	TUBB4A	protein_coding	20.68048446	15.26242205	18.25270473	4.221964073	6.085335788	2.647401651	-2.047427711	1.91E-06	http://www.ncbi.nlm.nih.gov/nuccore/NM_001289129.1
NM_001289127.1	mRNA	TUBB4A	protein_coding	20.68048446	15.26242205	18.25270473	4.221964073	6.085335788	2.647401651	-2.047427181	1.91E-06	http://www.ncbi.nlm.nih.gov/nuccore/NM_001289127.1
NM_015864.3	mRNA	FAM65B	protein_coding	9.352161105	7.659790533	12.15311108	1.084865168	3.044823316	2.735002413	-2.020466196	0.000551385	http://www.ncbi.nlm.nih.gov/nuccore/NM_015864.3
NM_001144915.1	mRNA	FGFR2	protein_coding	5.613110443	5.430449494	6.082802793	0.016927737	0.005313827	3.750577866	-2.019108493	0.009589919	http://www.ncbi.nlm.nih.gov/nuccore/NM_001144915.1
NM_153000.4	mRNA	APCDD1	protein_coding	4.647936888	6.472528901	6.082814707	1.064571214	2.028550331	0.882241933	-2.016270331	0.009042196	http://www.ncbi.nlm.nih.gov/nuccore/NM_153000.4
NM_001244847.1	mRNA	KRTDAP	protein_coding	1.801631411	6.418011359	5.066531866	1.057023398	2.025559632	0	-2.014757259	0.026850123	http://www.ncbi.nlm.nih.gov/nuccore/NM_001244847.1
NM_002652.2	mRNA	PIP	protein_coding	4.689126076	4.338389342	4.059281875	0.012728028	1.017695766	1.849053446	-2.013179474	0.024927531	http://www.ncbi.nlm.nih.gov/nuccore/NM_002652.2
NM_001207053.1	mRNA	KLK7	protein_coding	6.580041247	6.472451672	4.067489385	4.135541953	0.005320845	0	-2.011913292	0.010107369	http://www.ncbi.nlm.nih.gov/nuccore/NM_001207053.1
NM_001145018.1	mRNA	CCDC153	protein_coding	8.471123682	1.246384648	11.12638239	2.100818871	3.038931583	0	-1.997172333	0.009520078	http://www.ncbi.nlm.nih.gov/nuccore/NM_001145018.1
NM_001199159.1	mRNA	PI16	protein_coding	14.29310336	4.428621576	2.055312872	0.021127667	3.038921199	1.815060514	-1.996225097	0.016219873	http://www.ncbi.nlm.nih.gov/nuccore/NM_001199159.1
NM_001244995.1	mRNA	TMPRSS13	protein_coding	7.396488226	8.739977342	16.18787191	1.091054635	0.010532859	6.549767893	-1.990220489	0.000453983	http://www.ncbi.nlm.nih.gov/nuccore/NM_001244995.1
NM_006087.3	mRNA	TUBB4A	protein_coding	20.67010622	15.27320345	19.26251851	4.229926334	6.088199922	3.578255927	-1.970311108	2.82E-06	http://www.ncbi.nlm.nih.gov/nuccore/NM_006087.3
NM_004482.4	mRNA	GALNT3	protein_coding	20.67010622	15.27320345	19.26251851	4.229926334	6.088199922	3.578255927	-1.970310391	2.82E-06	https://www.ncbi.nlm.nih.gov/nuccore/NM_004482.4
NM_005291.2	mRNA	GPR17	protein_coding	5.544928945	9.686430443	9.120294512	1.078387294	1.024319116	3.709036455	-1.965892484	0.002267463	http://www.ncbi.nlm.nih.gov/nuccore/NM_005291.2
NM_001161417.1	mRNA	GPR17	protein_coding	5.544928945	9.686430443	9.120294512	1.078387294	1.024319116	3.709036455	-1.965888706	0.002266599	http://www.ncbi.nlm.nih.gov/nuccore/NM_001161417.1
NM_178554.4	mRNA	KY	protein_coding	5.623591768	10.60727467	0.018010911	2.091845753	0.005317334	1.831382139	-1.946328127	0.046105705	http://www.ncbi.nlm.nih.gov/nuccore/NM_178554.4
NM_178351.3	mRNA	LCE1C	protein_coding	1.778578584	2.272785069	12.1184004	3.114787036	1.020044013	0	-1.918312283	0.0464564	http://www.ncbi.nlm.nih.gov/nuccore/NM_178351.3
NM_000506.4	mRNA	F2	protein_coding	1.809753247	2.233818707	8.083070292	1.057017353	1.017698196	0.895945772	-1.901278485	0.048136433	http://www.ncbi.nlm.nih.gov/nuccore/NM_000506.4
NM_000397.3	mRNA	CYBB	protein_coding	2.769298862	3.282490064	6.070746642	2.082228163	0.003997341	0.895942512	-1.900037523	0.04162115	http://www.ncbi.nlm.nih.gov/nuccore/NM_000397.3
NM_001306212.1	mRNA	THBS4	protein_coding	4.700133425	5.365764274	2.040861713	2.08223591	1.017700628	0	-1.898063886	0.04158805	http://www.ncbi.nlm.nih.gov/nuccore/NM_001306212.1
NM_080474.2	mRNA	SERPINB12	protein_coding	6.639557373	4.325752505	1.03051988	0.012731282	2.025556048	0.895949032	-1.896689079	0.045627499	http://www.ncbi.nlm.nih.gov/nuccore/NM_080474.2
NM_012168.5	mRNA	FBXO2	protein_coding	5.669060913	5.365734942	1.030525449	1.057023398	2.025559632	0	-1.896631372	0.044288003	http://www.ncbi.nlm.nih.gov/nuccore/NM_012168.5
NM_020805.2	mRNA	KLHL14	protein_coding	51.35496516	66.88616329	58.81458013	13.75392228	19.28800102	14.457716	-1.8881822	8.53E-15	http://www.ncbi.nlm.nih.gov/nuccore/NM_020805.2
NM_023029.2	mRNA	FGFR2	protein_coding	7.525546811	5.4542144	6.087214416	0.02111162	0.006626824	4.691322771	-1.879448141	0.009591565	http://www.ncbi.nlm.nih.gov/nuccore/NM_023029.2
NM_001346031.1	mRNA	FAM65B	protein_coding	12.1666849	10.8882139	14.18590771	1.103019902	5.068858277	3.638660184	-1.876046667	0.000205226	http://www.ncbi.nlm.nih.gov/nuccore/NM_001346031.1
NM_001346032.1	mRNA	FAM65B	protein_coding	12.1666849	10.8882139	14.18590771	1.103019902	5.068858277	3.638660184	-1.876046624	0.000205208	http://www.ncbi.nlm.nih.gov/nuccore/NM_001346032.1
NM_001286445.2	mRNA	FAM65B	protein_coding	12.1666849	10.8882139	14.18590771	1.103019902	5.068858277	3.638660184	-1.876046621	0.000205207	http://www.ncbi.nlm.nih.gov/nuccore/NM_001286445.2
NM_014722.4	mRNA	FAM65B	protein_coding	12.1666849	10.8882139	14.18590771	1.103019902	5.068858277	3.638660184	-1.8760466	0.000205199	http://www.ncbi.nlm.nih.gov/nuccore/NM_014722.4
NM_001289130.1	mRNA	TUBB4A	protein_coding	15.92599849	13.0922885	18.2376858	4.221964073	6.085335788	2.647401651	-1.849493232	3.49E-05	http://www.ncbi.nlm.nih.gov/nuccore/NM_001289130.1
NM_001289131.1	mRNA	TUBB4A	protein_coding	15.92599849	13.0922885	18.2376858	4.221964073	6.085335788	2.647401651	-1.84949286	3.49E-05	http://www.ncbi.nlm.nih.gov/nuccore/NM_001289131.1
NM_001144916.1	mRNA	FGFR2	protein_coding	6.569081556	5.442457144	6.085033055	0.02111162	0.006626824	4.691322771	-1.803706021	0.014621281	http://www.ncbi.nlm.nih.gov/nuccore/NM_001144916.1
NM_001145411.1	mRNA	CCDC110	protein_coding	4.584225653	6.557439982	13.14729442	2.117228186	2.036422666	2.734993711	-1.754279993	0.006152669	http://www.ncbi.nlm.nih.gov/nuccore/NM_001145411.1
NM_006632.3	mRNA	SLC17A3	protein_coding	19.85794501	14.06857553	7.136713956	6.264901835	5.074685519	0.792385429	-1.754257406	0.000668464	http://www.ncbi.nlm.nih.gov/nuccore/NM_006632.3
NM_001169123.1	mRNA	AFF2	protein_coding	4.678343894	8.505574132	1.033446996	2.091845753	0.005317334	1.831382139	-1.748345334	0.046437751	http://www.ncbi.nlm.nih.gov/nuccore/NM_001169123.1
NM_002025.3	mRNA	AFF2	protein_coding	4.678343894	8.505574132	1.033446996	2.091845753	0.005317334	1.831382139	-1.748343192	0.046430882	http://www.ncbi.nlm.nih.gov/nuccore/NM_002025.3
NM_006488.2	mRNA	KHK	protein_coding	4.678444222	3.304970774	6.075757224	1.064563717	1.020037986	1.831387295	-1.742015606	0.036162987	http://www.ncbi.nlm.nih.gov/nuccore/NM_006488.2
NM_001161415.1	mRNA	GPR17	protein_coding	7.442644167	10.76656286	9.126998327	2.124867835	1.02824795	4.629074376	-1.738866467	0.002914018	http://www.ncbi.nlm.nih.gov/nuccore/NM_001161415.1
NM_001130046.1	mRNA	CCL20	protein_coding	10.27678477	12.92693948	10.14734174	2.139341747	1.031928327	6.507714702	-1.716851671	0.001157254	http://www.ncbi.nlm.nih.gov/nuccore/NM_001130046.1
NM_001077263.2	mRNA	TMPRSS13	protein_coding	12.14678574	10.91074703	16.20492608	1.114253269	0.015654741	10.30924978	-1.715604687	0.001242151	http://www.ncbi.nlm.nih.gov/nuccore/NM_001077263.2

NM_001204746.1	mRNA	CDH16	protein_coding	17.46431469	39.76716631	37.48333733	8.462825374	16.2096951	4.329165932	-1.70157526	3.55E-05	http://www.ncbi.nlm.nih.gov/nuccore/NM_001204746.1
NM_001267037.1	mRNA	SLC7A8	protein_coding	9.451292665	5.465698276	5.081518004	1.078407507	3.041956347	1.799734541	-1.700108775	0.01259754	http://www.ncbi.nlm.nih.gov/nuccore/NM_001267037.1
NM_001320804.1	mRNA	VWA2	protein_coding	7.48254845	7.590021411	8.110780937	3.144166241	1.026326048	2.734985008	-1.690979149	0.006984672	http://www.ncbi.nlm.nih.gov/nuccore/NM_001320804.1
NM_001272046.1	mRNA	VWA2	protein_coding	7.48254845	7.590021411	8.110780937	3.144166241	1.026326048	2.734985008	-1.690979084	0.006984145	http://www.ncbi.nlm.nih.gov/nuccore/NM_001272046.1
NM_001320135.1	mRNA	NTRK3	protein_coding	15.74466817	24.82378351	27.34958719	3.246378737	13.16193871	4.427416567	-1.687274737	2.64E-05	http://www.ncbi.nlm.nih.gov/nuccore/NM_001320135.1
NM_001161416.1	mRNA	GPR17	protein_coding	6.488838797	10.75341502	9.124807273	2.124867835	1.02824795	4.629074376	-1.68600394	0.00450365	http://www.ncbi.nlm.nih.gov/nuccore/NM_001161416.1
NM_001206789.1	mRNA	TMPRSS13	protein_coding	10.28697282	8.739795921	13.16722407	1.102946549	0.013105548	8.429757342	-1.677999893	0.001503083	http://www.ncbi.nlm.nih.gov/nuccore/NM_001206789.1
NM_001098486.1	mRNA	SLC17A3	protein_coding	20.81442349	14.08047124	7.138345658	6.274411989	5.077438362	1.708854952	-1.677735011	0.00085379	http://www.ncbi.nlm.nih.gov/nuccore/NM_001098486.1
NM_001206790.1	mRNA	TMPRSS13	protein_coding	10.25726188	10.86525773	14.18135506	2.146254199	2.045408041	6.487933774	-1.666923679	0.001038043	http://www.ncbi.nlm.nih.gov/nuccore/NM_001206790.1
NM_004591.2	mRNA	CCL20	protein_coding	11.19216859	14.03249781	13.1807713	3.185068608	2.047488939	6.468835299	-1.662746461	0.000598186	http://www.ncbi.nlm.nih.gov/nuccore/NM_004591.2
NM_000552.4	mRNA	VWF	protein_coding	2.709432143	11.69044	5.079589361	2.109202433	0.007938918	3.70902762	-1.648910173	0.028933209	http://www.ncbi.nlm.nih.gov/nuccore/NM_000552.4
NM_004062.3	mRNA	CDH16	protein_coding	18.3861514	41.91889999	39.50972065	10.54429426	17.22660563	4.295276635	-1.634407623	6.46E-05	http://www.ncbi.nlm.nih.gov/nuccore/NM_004062.3
NM_001144918.1	mRNA	FGFR2	protein_coding	6.558319743	6.497900325	6.087227099	0.025282936	1.024315062	4.669372727	-1.633781918	0.019138966	http://www.ncbi.nlm.nih.gov/nuccore/NM_001144918.1
NM_001080421.2	mRNA	UNC13A	protein_coding	8.459800726	7.577639352	6.093523659	1.084842545	1.026316966	4.648693837	-1.629815313	0.011133443	http://www.ncbi.nlm.nih.gov/nuccore/NM_001080421.2
NM_020877.3	mRNA	DNAH2	protein_coding	6.558346861	5.454252965	7.094514177	3.134930936	1.024327232	1.79972099	-1.629046686	0.018111148	http://www.ncbi.nlm.nih.gov/nuccore/NM_020877.3
NM_001142773.1	mRNA	PCDH15	protein_coding	10.43376978	4.407515039	4.071289311	0.02529569	3.041950491	2.751877885	-1.626267126	0.02223902	http://www.ncbi.nlm.nih.gov/nuccore/NM_001142773.1
NM_001142768.1	mRNA	PCDH15	protein_coding	10.43376978	4.407515039	4.071289311	0.02529569	3.041950491	2.751877885	-1.626265929	0.022242583	http://www.ncbi.nlm.nih.gov/nuccore/NM_001142768.1
NM_025184.3	mRNA	EFHC2	protein_coding	9.427733136	7.57759209	5.085429289	3.144198445	3.044836277	0.845565547	-1.624599311	0.011146723	http://www.ncbi.nlm.nih.gov/nuccore/NM_025184.3
NM_001309242.1	mRNA	MYO15B	protein_coding	8.368040433	11.85968039	11.15058384	2.139430499	6.076168038	1.745191531	-1.623850032	0.002444704	http://www.ncbi.nlm.nih.gov/nuccore/NM_001309242.1
NM_001297563.1	mRNA	TCEANC	protein_coding	6.498303636	7.614111728	11.1365949	6.223365692	1.028267991	0.834307354	-1.623046524	0.00770884	http://www.ncbi.nlm.nih.gov/nuccore/NM_001297563.1
NM_207392.2	mRNA	KRTDAP	protein_coding	7.245051785	27.73948874	17.23912408	5.282383609	10.12736263	1.664357988	-1.603021831	0.00361915	http://www.ncbi.nlm.nih.gov/nuccore/NM_207392.2
NM_021800.2	mRNA	DNAJC12	protein_coding	14.14271225	4.543204009	14.17653411	3.177464757	3.055147496	4.575148129	-1.574192525	0.003491073	http://www.ncbi.nlm.nih.gov/nuccore/NM_021800.2
NM_001007156.2	mRNA	NTRK3	protein_coding	24.0590083	44.12997756	41.54625211	8.520339244	20.26495243	7.991117153	-1.569794996	7.60E-06	http://www.ncbi.nlm.nih.gov/nuccore/NM_001007156.2
NM_019111.4	mRNA	HLA-DRA	protein_coding	3.626058241	8.645238526	12.14082053	3.153027169	4.055408693	0.834327402	-1.56941888	0.012492756	http://www.ncbi.nlm.nih.gov/nuccore/NM_019111.4
NM_000141.4	mRNA	FGFR2	protein_coding	10.37314487	6.55715852	7.105410231	2.124867835	1.02824795	4.629074376	-1.567807397	0.009970516	http://www.ncbi.nlm.nih.gov/nuccore/NM_000141.4
NM_001320658.1	mRNA	FGFR2	protein_coding	10.37314487	6.55715852	7.105410231	2.124867835	1.02824795	4.629074376	-1.56780731	0.009968897	http://www.ncbi.nlm.nih.gov/nuccore/NM_001320658.1
NM_199168.3	mRNA	CXCL12	protein_coding	6.537562855	5.477165248	9.113173294	2.117214224	1.026321506	3.690139049	-1.562743712	0.016468579	http://www.ncbi.nlm.nih.gov/nuccore/NM_199168.3
NM_004056.5	mRNA	CA8	protein_coding	5.572994311	7.565217992	8.106247249	3.14418234	2.03642828	1.785202588	-1.562063412	0.015913215	http://www.ncbi.nlm.nih.gov/nuccore/NM_004056.5
NM_001982.3	mRNA	ERBB3	protein_coding	7.396189164	17.0722088	8.129526413	2.146254199	2.045408041	6.487933774	-1.549960275	0.003897318	http://www.ncbi.nlm.nih.gov/nuccore/NM_001982.3
NM_001206850.1	mRNA	NLGN4Y	protein_coding	4.667974153	6.445906329	4.063495659	1.071660243	2.031326567	1.815054518	-1.543677735	0.047772871	http://www.ncbi.nlm.nih.gov/nuccore/NM_001206850.1
NM_001080423.3	mRNA	GRIP2	protein_coding	4.667995039	5.405570754	5.071069752	0.021127667	3.038921199	1.815060514	-1.543524513	0.048191819	http://www.ncbi.nlm.nih.gov/nuccore/NM_001080423.3
NM_001013626.2	mRNA	PPP1R42	protein_coding	4.668015921	4.362572674	6.078169028	0.021127667	3.038921199	1.815060514	-1.542995673	0.048502962	http://www.ncbi.nlm.nih.gov/nuccore/NM_001013626.2
NM_002530.3	mRNA	NTRK3	protein_coding	23.24659932	31.35235146	35.45878376	5.380490032	20.24576659	5.243209567	-1.536868091	8.60E-05	http://www.ncbi.nlm.nih.gov/nuccore/NM_002530.3
NM_012114.2	mRNA	CASP14	protein_coding	7.304864297	19.30623936	17.2215453	7.317930371	5.082727284	2.621633493	-1.527777906	0.001099885	http://www.ncbi.nlm.nih.gov/nuccore/NM_012114.2
NM_152775.3	mRNA	CCDC110	protein_coding	4.567435478	7.625942539	14.15908237	2.132279576	4.05831246	2.70360041	-1.522223509	0.01133967	http://www.ncbi.nlm.nih.gov/nuccore/NM_152775.3
NM_201262.1	mRNA	DNAJC12	protein_coding	12.28707931	8.669600899	5.092817926	2.132279576	4.05831246	2.70360041	-1.516319542	0.009393597	http://www.ncbi.nlm.nih.gov/nuccore/NM_201262.1
NM_003717.3	mRNA	NPFF	protein_coding	5.554264924	3.395280628	14.1508446	2.124867835	1.02824795	4.629074376	-1.512565494	0.0272604	http://www.ncbi.nlm.nih.gov/nuccore/NM_003717.3
NM_001291815.1	mRNA	HMCN2	protein_coding	3.658869354	7.552504739	9.110758568	3.14418234	2.03642828	1.785202588	-1.49441246	0.025140375	http://www.ncbi.nlm.nih.gov/nuccore/NM_001291815.1
NM_001012338.2	mRNA	NTRK3	protein_coding	23.24659932	31.35235146	35.45878376	5.386702757	20.2490611	6.17071894	-1.493024837	8.01E-05	http://www.ncbi.nlm.nih.gov/nuccore/NM_001012338.2
NM_152634.3	mRNA	TCEANC	protein_coding	5.582720372	6.51027865	8.1038979	5.190724785	1.026335139	0.845553425	-1.490547776	0.025163949	http://www.ncbi.nlm.nih.gov/nuccore/NM_152634.3
NM_001080446.2	mRNA	C11orf94	protein_coding	9.451292665	5.465698276	5.081518004	1.084876488	4.052350755	1.785217601	-1.489563422	0.025145038	http://www.ncbi.nlm.nih.gov/nuccore/NM_001080446.2
NM_012244.3	mRNA	SLC7A8	protein_coding	9.451292665	5.465698276	5.081518004	1.084876488	4.052350755	1.785217601	-1.489525162	0.025237782	http://www.ncbi.nlm.nih.gov/nuccore/NM_012244.3
NM_182728.2	mRNA	SLC7A8	protein_coding	9.451292665	5.465698276	5.081518004	1.084876488	4.052350755	1.785217601	-1.489524895	0.025238431	http://www.ncbi.nlm.nih.gov/nuccore/NM_182728.2
NM_001320134.1	mRNA	NTRK3	protein_coding	20.38297753	38.64685859	29.40704217	5.386606989	18.23409209	8.056762954	-1.469471198	0.000108828	http://www.ncbi.nlm.nih.gov/nuccore/NM_001320134.1
NM_001144914.1	mRNA	FGFR2	protein_coding	9.4885852	3.336676519	4.067458494	2.109202433	0.007938918	3.70902762	-1.469397817	0.048329494	http://www.ncbi.nlm.nih.gov/nuccore/NM_001144914.1
NM_001243101.1	mRNA	NTRK3	protein_coding	20.44740737	31.23617614	28.3828723	5.367722005	18.22452106	5.267347251	-1.46346456	0.000108493	http://www.ncbi.nlm.nih.gov/nuccore/NM_001243101.1
NM_004479.3	mRNA	FUT7	protein_coding	23.49411792	10.08168195	28.34510159	5.326957489	7.12019748	10.09390212	-1.439396305	0.000450682	http://www.ncbi.nlm.nih.gov/nuccore/NM_004479.3
NM_000070.2	mRNA	CAPN3	protein_coding	6.507866398	6.557346113	11.13411944	1.097138224	4.058303998	3.655093486	-1.406733906	0.018679568	http://www.ncbi.nlm.nih.gov/nuccore/NM_000070.2
NM_001012980.2	mRNA	SATL1	protein_coding	11.34189301	7.601944255	5.089178174	4.187495167	1.030124914	3.65505938	-1.402885212	0.019450416	http://www.ncbi.nlm.nih.gov/nuccore/NM_001012980.2
NM_030812.2	mRNA	ACTL8	protein_coding	3.650383624	10.68405244	7.099065167	3.153027169	4.055408693	0.834327402	-1.380996233	0.033915286	http://www.ncbi.nlm.nih.gov/nuccore/NM_030812.2
NM_001042416.2	mRNA	ZNF596	protein_coding	10.24748157	15.04875509	11.16132088	0.058015277	6.088148353	7.38862589	-1.373631676	0.003149307	http://www.ncbi.nlm.nih.gov/nuccore/NM_001042416.2
NM_019101.2	mRNA	APOM	protein_coding	16.10354801	8.728172902	6.110537533	2.152996304	4.066422769	5.512522585	-1.365976232	0.011119107	http://www.ncbi.nlm.nih.gov/nuccore/NM_019101.2
NM_001710.5	mRNA	CFB	protein_coding	366.069877	424.0275475	394.4546817	204.7994637	123.1121078	132.0365934	-1.364741922	1.16E-16	http://www.ncbi.nlm.nih.gov/nuccore/NM_001710.5
NM_001287399.1	mRNA	ZNF596	protein_coding	8.397096316	10.77950175	9.129153803	0.045855902	3.055121549	7.44718949	-1.352790357	0.008637795	http://www.ncbi.nlm.nih.gov/nuccore/NM_001287399.1
NM_001105556.2	mRNA	THEMIS2	protein_coding	8.349250662	16.04798253	9.139547863	4.222017	8.100095166	0.782498101	-1.346383574	0.007790214	http://www.ncbi.nlm.nih.gov/nuccore/NM_001105556.2
NM_144670.5	mRNA	A2ML1	protein_coding	5.55406628	10.71250229	7.103375254	4.187579329	5.065841001	0	-1.343695811	0.028616485	http://www.ncbi.nlm.nih.gov/nuccore/NM_144670.5
NM_001025231.1	mRNA	KPRP	protein_coding	7.482580091	6.545832219	9.117936699	4.187579329	5.065841001	0	-1.340511007	0.02684053	http://www.ncbi.nlm.nih.gov/nuccore/NM_001025231.1
NM_001942.3	mRNA	DSG1	protein_coding	31.44650407	65.53243681	52.71353092	23.1040764	29.40272158	6.811825637	-1.334233188	0.000759256	http://www.ncbi.nlm.nih.gov/nuccore/NM_001942.3
NM_002411.3	mRNA	SCGB2A2	protein_coding	11.05984986	17.34477446	25.30238905	0.089638145	9.134430785	12.02279765	-1.31116469	0.009381783	http://www.ncbi.nlm.nih.gov/nuccore/NM_002411.3
NM_173087.1	mRNA	CAPN3	protein_coding	4.61957382	5.465896914	10.11735491	1.091104511	4.055392971	2.718959887	-1.310804633	0.045971882	http://www.ncbi.nlm.nih.gov/nuccore/NM_173087.1
NM_181871.3	mRNA	PKLR	protein_coding	4.619524593	7.552459856	8.103912468	3.153009595	3.047569734	1.771340413	-1.309880679	0.043153571	http://www.ncbi.nlm.nih.gov/nuccore/NM_181871.3
NM_000298.5	mRNA	PKLR	protein_coding	4.619524593	7.552459856	8.103912468	3.153009595	3.047569734	1.771340413	-1.3098804	0.043149888	http://www.ncbi.nlm.nih.gov/nuccore/NM_000298.5
NM_001144917.1	mRNA	FGFR2	protein_coding	9.451260489	6.51010918	4.073165332	2.124867835	1.02824795	4.629074376	-1.309580122	0.046133857	http://www.ncbi.nlm.nih.gov/nuccore/NM_001144917.1
NM_001004019.1	mRNA	FBLN2	protein_coding	5.51841456	7.637435871	14.16170757	3.177443214	2.045415628	5.530428151	-1.298917376	0.022908183	http://www.ncbi.nlm.nih.gov/nuccore/NM_001004019.1
NM_001998.2	mRNA	FBLN2	protein_coding	5.51841456	7.637435871	14.16170757	3.177443214	2.045415628	5.530428151	-1.29891512	0.022903085	http://www.ncbi.nlm.nih.gov/nuccore/NM_001998.2
NM_001280547.1	mRNA	PAX5	protein_coding	6.402279989	20.24584479	10.15357075	2.172143311	4.073819892	8.326970985	-1.295950254	0.009973491	http://www.ncbi.nlm.nih.gov/nuccore/NM_001280547.1

NM_001280555.1	mRNA	PAX5	protein_coding	6.402279989	20.24584479	10.15357075	2.172143311	4.073819892	8.326970985	-1.295944581	0.009969666	http://www.ncbi.nlm.nih.gov/nuccore/NM_001280555.1
NM_024344.1	mRNA	CAPN3	protein_coding	6.527485004	5.488309546	10.12240664	1.097138224	4.058303998	3.655093486	-1.282558163	0.037751113	http://www.ncbi.nlm.nih.gov/nuccore/NM_024344.1
NM_001003795.2	mRNA	GTF2IRD2B	protein_coding	21.45210854	22.81044579	30.38761242	12.59113544	11.17530016	7.125634226	-1.270751932	0.000112614	http://www.ncbi.nlm.nih.gov/nuccore/NM_001003795.2
NM_001127699.1	mRNA	SPINK5	protein_coding	6.348445298	16.18097673	21.24979783	6.318497019	10.13054478	1.653751756	-1.26649652	0.013695498	http://www.ncbi.nlm.nih.gov/nuccore/NM_001127699.1
NM_001042415.2	mRNA	ZNF596	protein_coding	9.331989598	11.85961315	10.14312519	0.053978257	5.077369233	7.407525321	-1.262963625	0.009175748	http://www.ncbi.nlm.nih.gov/nuccore/NM_001042415.2
NM_148964.2	mRNA	CTSE	protein_coding	13.19724949	11.85934432	6.110587043	3.192599289	7.092854543	2.647414483	-1.254185735	0.015823173	http://www.ncbi.nlm.nih.gov/nuccore/NM_148964.2
NM_001317331.1	mRNA	CTSE	protein_coding	13.19724949	11.85934432	6.110587043	3.192599289	7.092854543	2.647414483	-1.254184534	0.015826731	http://www.ncbi.nlm.nih.gov/nuccore/NM_001317331.1
NM_001165035.1	mRNA	FBLN2	protein_coding	4.567435478	7.625942539	14.15908237	3.177443214	2.045415628	5.530428151	-1.247030484	0.033905815	http://www.ncbi.nlm.nih.gov/nuccore/NM_001165035.1
NM_025268.3	mRNA	TMEM121	protein_coding	20.86386154	7.753355752	9.14898698	5.274216013	5.085238503	5.44607154	-1.244472407	0.011568575	http://www.ncbi.nlm.nih.gov/nuccore/NM_025268.3
NM_173653.3	mRNA	SLC9A9	protein_coding	8.417232902	10.75329649	7.109555617	2.146254199	2.045408041	6.487933774	-1.244360632	0.027196813	http://www.ncbi.nlm.nih.gov/nuccore/NM_173653.3
NM_014470.3	mRNA	RND1	protein_coding	5.526883954	15.94392997	5.092917106	6.255062297	4.063848987	0.802492764	-1.243358797	0.040294991	http://www.ncbi.nlm.nih.gov/nuccore/NM_014470.3
NM_001166035.1	mRNA	SBSN	protein_coding	11.10998511	18.31632539	18.23783867	9.414732413	9.128981606	1.633051861	-1.242068422	0.003025986	http://www.ncbi.nlm.nih.gov/nuccore/NM_001166035.1
NM_000954.5	mRNA	PTGDS	protein_coding	23.55675344	18.42345997	14.22432344	9.45021215	6.113477459	8.172962566	-1.234503253	0.001188022	http://www.ncbi.nlm.nih.gov/nuccore/NM_000954.5
NM_001287255.1	mRNA	ZNF596	protein_coding	8.397096316	10.77950175	9.129153803	0.049925163	4.066402566	7.427025121	-1.230680042	0.014952276	http://www.ncbi.nlm.nih.gov/nuccore/NM_001287255.1
NM_001287256.1	mRNA	ZNF596	protein_coding	8.397096316	10.77950175	9.129153803	0.049925163	4.066402566	7.427025121	-1.230678135	0.014950361	http://www.ncbi.nlm.nih.gov/nuccore/NM_001287256.1
NM_173539.3	mRNA	ZNF596	protein_coding	8.397096316	10.77950175	9.129153803	0.049925163	4.066402566	7.427025121	-1.230675938	0.014948154	http://www.ncbi.nlm.nih.gov/nuccore/NM_173539.3
NM_016644.2	mRNA	PRR16	protein_coding	5.509649763	13.89934625	9.129208441	4.213777405	6.082385716	1.720647067	-1.226790198	0.019429079	http://www.ncbi.nlm.nih.gov/nuccore/NM_016644.2
NM_001280549.1	mRNA	PAX5	protein_coding	6.418577618	20.21532547	8.133386395	2.172143311	4.073819892	8.326970985	-1.217865378	0.020038524	http://www.ncbi.nlm.nih.gov/nuccore/NM_001280549.1
NM_001280551.1	mRNA	PAX5	protein_coding	6.418651038	18.13992907	10.14950011	2.172143311	4.073819892	8.326970985	-1.20750591	0.012287747	http://www.ncbi.nlm.nih.gov/nuccore/NM_001280551.1
NM_001280552.1	mRNA	PAX5	protein_coding	6.418651038	18.13992907	10.14950011	2.172143311	4.073819892	8.326970985	-1.2075036	0.012286017	http://www.ncbi.nlm.nih.gov/nuccore/NM_001280552.1
NM_139211.4	mRNA	HOPX	protein_coding	15.83098529	16.3427384	25.31183578	8.431490042	14.18218697	2.507713461	-1.196674303	0.003597111	http://www.ncbi.nlm.nih.gov/nuccore/NM_139211.4
NM_001145459.1	mRNA	HOPX	protein_coding	15.83098529	16.3427384	25.31183578	8.431490042	14.18218697	2.507713461	-1.196672411	0.003598646	http://www.ncbi.nlm.nih.gov/nuccore/NM_001145459.1
NM_001206480.2	mRNA	ELMO1	protein_coding	7.462330581	6.568578249	11.13657628	3.177400155	0.014392257	7.447138714	-1.19005328	0.043843256	http://www.ncbi.nlm.nih.gov/nuccore/NM_001206480.2
NM_001206482.1	mRNA	ELMO1	protein_coding	7.462330581	6.568578249	11.13657628	3.177400155	0.014392257	7.447138714	-1.190051067	0.043836481	http://www.ncbi.nlm.nih.gov/nuccore/NM_001206482.1
NM_014800.10	mRNA	ELMO1	protein_coding	7.462330581	6.568578249	11.13657628	3.177400155	0.014392257	7.447138714	-1.190047697	0.043826171	http://www.ncbi.nlm.nih.gov/nuccore/NM_014800.10
NM_001184780.1	mRNA	NOX5	protein_coding	194.6152158	203.699238	244.2908178	67.85592404	149.1211128	64.55630581	-1.188590953	4.01E-06	http://www.ncbi.nlm.nih.gov/nuccore/NM_001184780.1
NM_006775.3	mRNA	QKI	protein_coding	194.6152158	203.699238	244.2908178	67.85592404	149.1211128	64.55630581	-1.188590839	4.02E-06	https://www.ncbi.nlm.nih.gov/nuccore/NM_006775.3
NM_001267053.3	mRNA	RIBC1	protein_coding	10.3616557	8.657394079	6.099480728	2.146235303	1.033697629	7.447155641	-1.188398917	0.041153822	http://www.ncbi.nlm.nih.gov/nuccore/NM_001267053.3
NM_000423.2	mRNA	KRT2	protein_coding	77.37384498	148.0791328	70.07988593	51.45500804	67.93022261	9.950897202	-1.187528585	0.01340705	http://www.ncbi.nlm.nih.gov/nuccore/NM_000423.2
NM_001910.3	mRNA	CTSE	protein_coding	14.13105903	12.93946388	7.124718467	4.237713539	7.098866545	3.564236337	-1.185634223	0.014995218	http://www.ncbi.nlm.nih.gov/nuccore/NM_001910.3
NM_024505.3	mRNA	NOX5	protein_coding	194.6070464	203.7098955	246.314976	67.86418076	149.1256555	66.40359245	-1.183740558	3.47E-06	http://www.ncbi.nlm.nih.gov/nuccore/NM_024505.3
NM_001331238.1	mRNA	TMEM121	protein_coding	17.03615504	7.713044591	9.141360847	4.237655846	5.08268963	5.461995223	-1.182817913	0.016302144	http://www.ncbi.nlm.nih.gov/nuccore/NM_001331238.1
NM_004820.4	mRNA	CYP7B1	protein_coding	20.5171693	15.4332408	35.41761649	12.59908732	2.081952181	16.61620644	-1.178179552	0.012210232	http://www.ncbi.nlm.nih.gov/nuccore/NM_004820.4
NM_021012.4	mRNA	KCNJ12	protein_coding	12.27533892	6.590359702	8.119353466	5.240092484	4.066453114	2.660776719	-1.167742207	0.031660315	http://www.ncbi.nlm.nih.gov/nuccore/NM_021012.4
NM_001280548.1	mRNA	PAX5	protein_coding	6.410413148	19.19289252	10.15154869	2.178252093	4.076161025	9.265466312	-1.164781705	0.018997674	http://www.ncbi.nlm.nih.gov/nuccore/NM_001280548.1
NM_001280553.1	mRNA	PAX5	protein_coding	6.410413148	19.19289252	10.15154869	2.178252093	4.076161025	9.265466312	-1.16477467	0.018990324	http://www.ncbi.nlm.nih.gov/nuccore/NM_001280553.1
NM_001280554.1	mRNA	PAX5	protein_coding	6.394247836	21.29880157	10.15556721	2.184268101	5.087674099	9.246374395	-1.164006218	0.022540784	http://www.ncbi.nlm.nih.gov/nuccore/NM_001280554.1
NM_001280556.1	mRNA	PAX5	protein_coding	6.394247836	21.29880157	10.15556721	2.184268101	5.087674099	9.246374395	-1.164001701	0.022534874	http://www.ncbi.nlm.nih.gov/nuccore/NM_001280556.1
NM_016734.2	mRNA	PAX5	protein_coding	6.394247836	21.29880157	10.15556721	2.184268101	5.087674099	9.246374395	-1.163998667	0.022530906	http://www.ncbi.nlm.nih.gov/nuccore/NM_016734.2
NM_139212.3	mRNA	HOPX	protein_coding	16.76638278	16.3747016	27.3333855	8.447455989	16.20339247	2.487062409	-1.15652213	0.007293652	http://www.ncbi.nlm.nih.gov/nuccore/NM_139212.3
NM_032495.5	mRNA	HOPX	protein_coding	16.76638278	16.3747016	27.3333855	8.447455989	16.20339247	2.487062409	-1.156520241	0.007296355	http://www.ncbi.nlm.nih.gov/nuccore/NM_032495.5
NM_001145460.1	mRNA	HOPX	protein_coding	16.76638278	16.3747016	27.3333855	8.447455989	16.20339247	2.487062409	-1.156517449	0.007300352	http://www.ncbi.nlm.nih.gov/nuccore/NM_001145460.1
NM_001324112.1	mRNA	CYP7B1	protein_coding	8.349528305	8.751034412	16.19040315	4.237569391	2.053416963	8.326928572	-1.150789016	0.024259101	http://www.ncbi.nlm.nih.gov/nuccore/NM_001324112.1
NM_020311.2	mRNA	ACKR3	protein_coding	16.83113707	19.43332455	17.24297963	15.59904057	6.113582582	2.518128262	-1.149388812	0.005495065	http://www.ncbi.nlm.nih.gov/nuccore/NM_020311.2
NM_174945.2	mRNA	ZNF575	protein_coding	13.06245345	12.01263358	19.23806824	9.414594286	6.104023867	4.440561527	-1.147437059	0.007016727	http://www.ncbi.nlm.nih.gov/nuccore/NM_174945.2
NM_001287254.1	mRNA	ZNF596	protein_coding	12.18698345	13.99422691	9.143426529	2.178300524	6.093672335	7.352377448	-1.141244421	0.016657971	http://www.ncbi.nlm.nih.gov/nuccore/NM_001287254.1
NM_024421.2	mRNA	DSC1	protein_coding	7.370172022	12.95262255	15.18849608	4.245346562	9.116894006	2.609272714	-1.139152155	0.019972878	http://www.ncbi.nlm.nih.gov/nuccore/NM_024421.2
NM_006846.3	mRNA	SPINK5	protein_coding	9.181870804	16.2415255	23.27606648	7.379116363	13.16526515	1.613056317	-1.133435747	0.019724168	http://www.ncbi.nlm.nih.gov/nuccore/NM_006846.3
NM_001127698.1	mRNA	SPINK5	protein_coding	9.181870804	16.2415255	23.27606648	7.379116363	13.16526515	1.613056317	-1.133433723	0.019733939	http://www.ncbi.nlm.nih.gov/nuccore/NM_001127698.1
NM_001003940.1	mRNA	BMF	protein_coding	23.46257362	26.88627984	15.24903426	10.5279869	12.18104494	7.138998469	-1.131703291	0.001166144	http://www.ncbi.nlm.nih.gov/nuccore/NM_001003940.1
NM_001288741.1	mRNA	C10orf128	protein_coding	15.74446588	27.95983963	24.32668737	10.53616611	14.20010686	6.183053279	-1.131653988	0.001318441	http://www.ncbi.nlm.nih.gov/nuccore/NM_001288741.1
NM_001280550.1	mRNA	PAX5	protein_coding	6.435385977	18.11006591	8.129545042	2.172143311	4.073819892	8.326970985	-1.123740065	0.024221232	http://www.ncbi.nlm.nih.gov/nuccore/NM_001280550.1
NM_001007189.1	mRNA	IGIP	protein_coding	25.11015147	33.52425287	38.49718971	15.79012914	14.23645718	14.50066356	-1.116281484	7.40E-05	http://www.ncbi.nlm.nih.gov/nuccore/NM_001007189.1
NM_001286113.1	mRNA	THEMIS2	protein_coding	8.417268213	9.712247917	8.117311264	3.185205315	8.096776881	0.792412426	-1.115829583	0.045937146	http://www.ncbi.nlm.nih.gov/nuccore/NM_001286113.1
NM_001080527.1	mRNA	MYO7B	protein_coding	9.142738005	23.60507933	21.27359431	7.125130415	11.5114714	6.262000821	-1.106055793	0.007119541	http://www.ncbi.nlm.nih.gov/nuccore/NM_001080527.1
NM_001190348.1	mRNA	DMKN	protein_coding	24.26697375	28.13338798	30.4046997	17.78619934	19.26042829	1.468749022	-1.105260634	0.011677783	http://www.ncbi.nlm.nih.gov/nuccore/NM_001190348.1
NM_001740.4	mRNA	CALB2	protein_coding	11.98614116	33.02506882	13.21794898	15.62996698	6.120258236	5.291965124	-1.102019524	0.023584229	http://www.ncbi.nlm.nih.gov/nuccore/NM_001740.4
NM_001288740.1	mRNA	C10orf128	protein_coding	17.57567854	30.17962626	31.40425233	15.72393782	15.22501859	6.110516755	-1.094601886	0.00152177	http://www.ncbi.nlm.nih.gov/nuccore/NM_001288740.1
NM_001010863.2	mRNA	C10orf128	protein_coding	17.57567854	30.17962626	31.40425233	15.72393782	15.22501859	6.110516755	-1.094601358	0.001522548	http://www.ncbi.nlm.nih.gov/nuccore/NM_001010863.2
NM_001014440.3	mRNA	ODF3B	protein_coding	21.59410705	18.45775468	19.27080791	10.51132964	9.150303663	8.112975972	-1.090772915	0.002261156	http://www.ncbi.nlm.nih.gov/nuccore/NM_001014440.3
NM_207336.2	mRNA	ZNF467	protein_coding	18.70121365	20.54925718	20.27872824	9.483769891	13.1835312	5.27958389	-1.090533351	0.002612643	http://www.ncbi.nlm.nih.gov/nuccore/NM_207336.2
NM_001288742.1	mRNA	C10orf128	protein_coding	17.64896716	27.98081014	24.33080368	10.55206751	16.22178646	6.158272979	-1.08336901	0.001652522	http://www.ncbi.nlm.nih.gov/nuccore/NM_001288742.1
NM_007088.3	mRNA	CALB2	protein_coding	11.1360431	24.51832701	9.160103917	12.49741619	5.097205617	3.486428225	-1.08227234	0.030243186	http://www.ncbi.nlm.nih.gov/nuccore/NM_007088.3
NM_006095.2	mRNA	ATP8A1	protein_coding	24.34839513	33.24774548	16.2715148	14.67941542	11.18533836	8.96109416	-1.080031064	0.001715639	http://www.ncbi.nlm.nih.gov/nuccore/NM_006095.2
NM_173481.3	mRNA	MISP	protein_coding	31.5859951	43.29088078	53.68240689	27.23206189	18.31090483	15.22843706	-1.079886388	0.000111055	http://www.ncbi.nlm.nih.gov/nuccore/NM_173481.3
NM_003641.3	mRNA	IFITM1	protein_coding	650.3814471	610.634248	598.3012166	430.7755793	213.6698295	235.1072805	-1.078483317	5.03E-08	http://www.ncbi.nlm.nih.gov/nuccore/NM_003641.3

NM_001285485.1	mRNA	NEURL3	protein_coding	74.10192503	73.51721144	61.89340552	42.98809361	27.48217332	28.88183214	-1.077676322	3.17E-07	http://www.ncbi.nlm.nih.gov/nuccore/NM_001285485.1
NM_001285486.1	mRNA	NEURL3	protein_coding	77.84178773	74.68003384	68.97700397	44.06618003	32.54474312	28.80886708	-1.07416279	8.03E-08	http://www.ncbi.nlm.nih.gov/nuccore/NM_001285486.1
NM_001024940.2	mRNA	TRIM17	protein_coding	3.559002057	16.04837652	14.17679343	4.245286546	7.101747033	4.496614287	-1.067142201	0.039818954	http://www.ncbi.nlm.nih.gov/nuccore/NM_001024940.2
NM_001003943.2	mRNA	BMF	protein_coding	21.58358088	24.73684947	14.23162216	10.51974896	11.17003549	7.152506837	-1.065409614	0.003467415	http://www.ncbi.nlm.nih.gov/nuccore/NM_001003943.2
NM_001321187.1	mRNA	FAM156B	protein_coding	12.20800792	13.96792799	7.122937929	5.274118789	2.0553148	8.308154871	-1.058225072	0.033234241	http://www.ncbi.nlm.nih.gov/nuccore/NM_001321187.1
NM_001288743.1	mRNA	C10orf128	protein_coding	20.42283288	31.27207749	31.41273158	15.74970687	17.24981349	7.013702565	-1.054816495	0.001091859	http://www.ncbi.nlm.nih.gov/nuccore/NM_001288743.1
NM_001012302.2	mRNA	ANO9	protein_coding	12.2298813	8.72841066	10.1430654	5.266024701	5.082702175	4.511427168	-1.054256327	0.033030483	http://www.ncbi.nlm.nih.gov/nuccore/NM_001012302.2
NM_001347882.1	mRNA	ANO9	protein_coding	12.20823805	8.75078888	12.16229701	5.274248446	6.093714141	4.496596875	-1.052460519	0.028013177	http://www.ncbi.nlm.nih.gov/nuccore/NM_001347882.1
NM_006610.3	mRNA	MASP2	protein_coding	12.03671618	17.32131745	22.27615467	5.341050553	13.17456659	6.262152128	-1.046796198	0.007939739	http://www.ncbi.nlm.nih.gov/nuccore/NM_006610.3
NM_001964.2	mRNA	EGR1	protein_coding	38.01813263	55.23585093	74.93691622	25.34288453	26.43595656	30.0545346	-1.033712766	9.89E-05	http://www.ncbi.nlm.nih.gov/nuccore/NM_001964.2
NM_139055.3	mRNA	ADAMTS15	protein_coding	26.05347253	40.87205428	32.45276264	12.72968475	16.2640494	19.18602628	-1.028788901	0.00026753	http://www.ncbi.nlm.nih.gov/nuccore/NM_139055.3
NM_001128636.2	mRNA	ELFN1	protein_coding	36.31051627	55.9719571	45.63283479	20.08186435	25.39165593	21.74908171	-1.028543138	2.89E-05	http://www.ncbi.nlm.nih.gov/nuccore/NM_001128636.2
NM_016102.3	mRNA	TRIM17	protein_coding	3.538336826	17.12995969	16.19799255	5.290141712	8.115325936	4.467996991	-1.02601812	0.04888768	http://www.ncbi.nlm.nih.gov/nuccore/NM_016102.3
NM_003725.3	mRNA	HSD17B6	protein_coding	21.8208304	6.709973856	10.15913174	9.405239843	6.101543732	3.511299707	-1.024262176	0.041899495	http://www.ncbi.nlm.nih.gov/nuccore/NM_003725.3
NM_001126057.2	mRNA	DMKN	protein_coding	21.44308164	25.96182463	28.37444005	16.75087824	19.25742807	1.477125709	-1.016701166	0.023171126	http://www.ncbi.nlm.nih.gov/nuccore/NM_001126057.2
NM_002120.3	mRNA	HLA-DOB	protein_coding	5.476609937	12.91430235	14.17433154	8.351857403	2.055343971	5.446015537	-1.01552391	0.038734464	http://www.ncbi.nlm.nih.gov/nuccore/NM_002120.3
NM_004948.3	mRNA	DSC1	protein_coding	7.36168242	12.96510844	16.19788848	4.260027397	10.13050831	3.524166822	-1.013877658	0.035528821	http://www.ncbi.nlm.nih.gov/nuccore/NM_004948.3
NM_002339.2	mRNA	LSP1	protein_coding	13.06256081	9.915449414	21.25208368	9.432839229	8.126357854	4.414280371	-1.009792762	0.019450116	http://www.ncbi.nlm.nih.gov/nuccore/NM_002339.2
NM_001242932.1	mRNA	LSP1	protein_coding	14.02668475	8.864347431	21.25205178	9.432839229	8.126357854	4.414280371	-1.008036292	0.020849581	http://www.ncbi.nlm.nih.gov/nuccore/NM_001242932.1
NM_002608.3	mRNA	PDGFB	protein_coding	44.3765213	87.33755278	93.20085484	28.63623848	52.7507363	30.60980477	-1.005150992	0.001013102	http://www.ncbi.nlm.nih.gov/nuccore/NM_002608.3
NM_001190349.1	mRNA	DMKN	protein_coding	21.52525035	22.7210035	22.30874763	14.66225906	17.22975114	1.511337642	-1.001778783	0.014741524	http://www.ncbi.nlm.nih.gov/nuccore/NM_001190349.1
NM_001314051.1	mRNA	ANGPT1	protein_coding	4.689087651	6.417912423	2.042612534	9.47540288	8.138939833	9.076787802	1.005850473	0.047873073	http://www.ncbi.nlm.nih.gov/nuccore/NM_001314051.1
NM_001077196.1	mRNA	PDE11A	protein_coding	10.35040642	9.712134224	6.101410366	24.08847555	14.25703837	15.32033837	1.020176577	0.005500491	http://www.ncbi.nlm.nih.gov/nuccore/NM_001077196.1
NM_152349.2	mRNA	KRT222	protein_coding	6.579964685	9.587488431	1.037628127	15.71464622	8.159388706	11.78838953	1.03847264	0.025867492	http://www.ncbi.nlm.nih.gov/nuccore/NM_152349.2
NM_006725.4	mRNA	CD6	protein_coding	4.667995039	5.405570754	5.071069752	8.484589417	8.150518128	14.70742418	1.043238333	0.035378071	http://www.ncbi.nlm.nih.gov/nuccore/NM_006725.4
NM_001185100.1	mRNA	CD22	protein_coding	4.689126076	4.338389342	4.059281875	8.454674579	6.122280625	12.86922037	1.055185229	0.049626211	http://www.ncbi.nlm.nih.gov/nuccore/NM_001185100.1
NM_199355.3	mRNA	ADAMTS18	protein_coding	4.689106865	5.37939814	3.051432455	14.61531989	7.132130002	6.221498087	1.063011729	0.040670286	http://www.ncbi.nlm.nih.gov/nuccore/NM_199355.3
NM_001326358.1	mRNA	ADAMTS18	protein_coding	4.689106865	5.37939814	3.051432455	14.61531989	7.132130002	6.221498087	1.063013729	0.040677452	http://www.ncbi.nlm.nih.gov/nuccore/NM_001326358.1
NM_001276460.1	mRNA	FAM26F	protein_coding	12.31144889	6.557064796	5.08916464	11.71756747	17.28025036	21.98988517	1.074581468	0.006605922	http://www.ncbi.nlm.nih.gov/nuccore/NM_001276460.1
NM_139072.3	mRNA	DNER	protein_coding	19.72524787	17.34388445	16.23501023	43.10240314	30.5420007	40.0088374	1.085320181	2.90E-05	http://www.ncbi.nlm.nih.gov/nuccore/NM_139072.3
NM_001271889.1	mRNA	CIB2	protein_coding	4.678343894	8.505574132	1.033446996	5.380066657	11.17512442	13.77122095	1.088098366	0.042634592	http://www.ncbi.nlm.nih.gov/nuccore/NM_001271889.1
NM_170706.3	mRNA	NMNAT2	protein_coding	3.713280725	4.350664188	6.075767869	8.477463548	11.17519967	10.91528408	1.098052006	0.021319227	http://www.ncbi.nlm.nih.gov/nuccore/NM_170706.3
NM_001035.2	mRNA	RYR2	protein_coding	8.438178093	7.602092913	8.112984877	9.65585901	21.31808818	21.02344362	1.099258847	0.005058199	http://www.ncbi.nlm.nih.gov/nuccore/NM_001035.2
NM_001771.3	mRNA	CD22	protein_coding	4.689126076	4.338389342	4.059281875	8.462290573	6.124401325	13.80591101	1.104271708	0.0377601	http://www.ncbi.nlm.nih.gov/nuccore/NM_001771.3
NM_001083.3	mRNA	PDE5A	protein_coding	28.96934553	41.86716535	24.37746034	89.17110077	91.34273561	24.23012059	1.10618848	0.0074477	http://www.ncbi.nlm.nih.gov/nuccore/NM_001083.3
NM_001164556.1	mRNA	DISC1	protein_coding	7.548533627	2.281875251	7.089799263	8.526358994	8.163407227	20.32637152	1.107657282	0.027346186	http://www.ncbi.nlm.nih.gov/nuccore/NM_001164556.1
NM_001318534.1	mRNA	ZNF311	protein_coding	5.634531073	7.484198075	2.045987652	9.529410713	5.123603555	18.4900702	1.116556378	0.038977014	http://www.ncbi.nlm.nih.gov/nuccore/NM_001318534.1
NM_033437.3	mRNA	PDE5A	protein_coding	27.0812064	39.72798844	24.37058451	87.06106396	88.29831812	23.34338424	1.125313625	0.006596038	http://www.ncbi.nlm.nih.gov/nuccore/NM_033437.3
NM_033430.2	mRNA	PDE5A	protein_coding	27.0812064	39.72798844	24.37058451	87.06106396	88.29831812	23.34338424	1.12531378	0.006597767	http://www.ncbi.nlm.nih.gov/nuccore/NM_033430.2
NM_020404.2	mRNA	CD248	protein_coding	4.628715164	10.65431058	4.071355341	13.71687552	19.27455781	9.79979221	1.136001732	0.007730603	http://www.ncbi.nlm.nih.gov/nuccore/NM_020404.2
NM_030979.2	mRNA	PABPC3	protein_coding	11.33005108	7.613858381	6.099465984	12.78277744	16.28244313	26.66365737	1.143710957	0.003187693	http://www.ncbi.nlm.nih.gov/nuccore/NM_030979.2
NM_080829.3	mRNA	FAM65C	protein_coding	8.505905062	3.346887924	6.085008463	14.73003036	18.26080334	7.941308736	1.153550895	0.008728884	http://www.ncbi.nlm.nih.gov/nuccore/NM_080829.3
NM_001347826.1	mRNA	OGDHL	protein_coding	7.548452942	5.430377212	4.067479083	18.82069691	10.18548	9.851939896	1.159108376	0.00909751	http://www.ncbi.nlm.nih.gov/nuccore/NM_001347826.1
NM_006438.4	mRNA	COLEC10	protein_coding	10.38481037	5.499090025	7.103300637	16.88109634	17.28280994	18.17551558	1.165321479	0.002073113	http://www.ncbi.nlm.nih.gov/nuccore/NM_006438.4
NM_148959.3	mRNA	HUS1B	protein_coding	7.525661098	1.231323015	10.11468717	12.69405814	14.23397823	16.41395157	1.169708464	0.006749054	http://www.ncbi.nlm.nih.gov/nuccore/NM_148959.3
NM_153366.3	mRNA	SVEP1	protein_coding	16.7935259	18.43565539	22.28972708	52.51176689	46.73934521	32.27502756	1.185018487	7.27E-06	http://www.ncbi.nlm.nih.gov/nuccore/NM_153366.3
NM_002279.4	mRNA	KRT33B	protein_coding	3.713299204	3.304997816	7.082361004	6.427389775	13.19731553	12.78551138	1.185420708	0.017943929	http://www.ncbi.nlm.nih.gov/nuccore/NM_002279.4
NM_001292024.1	mRNA	LOC100129940	protein_coding	9.416337619	7.589924538	6.095539498	19.97628405	13.24707183	20.05771046	1.190735369	0.001715425	http://www.ncbi.nlm.nih.gov/nuccore/NM_001292024.1
NM_001145938.1	mRNA	MMP1	protein_coding	139.6392734	141.7125593	128.8100987	367.3379136	247.1734364	324.1938406	1.193760973	1.62E-14	http://www.ncbi.nlm.nih.gov/nuccore/NM_001145938.1
NM_007332.2	mRNA	TRPA1	protein_coding	17.81521598	19.40866341	14.21504929	43.13352733	45.70030722	29.60021843	1.193939843	1.05E-05	http://www.ncbi.nlm.nih.gov/nuccore/NM_007332.2
NM_001190730.1	mRNA	RALGPS1	protein_coding	5.64571652	6.432061421	2.044315098	12.60843487	16.2218472	4.284241959	1.200112021	0.021788799	http://www.ncbi.nlm.nih.gov/nuccore/NM_001190730.1
NM_015039.3	mRNA	NMNAT2	protein_coding	2.760117999	4.338446226	6.073299214	8.477463548	11.17519967	10.91528408	1.201607189	0.013298481	http://www.ncbi.nlm.nih.gov/nuccore/NM_015039.3
NM_014568.2	mRNA	GALNT5	protein_coding	4.689106865	5.37939814	3.051432455	16.69406627	7.138800641	7.125515983	1.209175246	0.018328745	http://www.ncbi.nlm.nih.gov/nuccore/NM_014568.2
NM_001329868.1	mRNA	GALNT5	protein_coding	4.689106865	5.37939814	3.051432455	16.69406627	7.138800641	7.125515983	1.209176942	0.018332492	http://www.ncbi.nlm.nih.gov/nuccore/NM_001329868.1
NM_002421.3	mRNA	MMP1	protein_coding	165.9179451	160.2148153	153.1402952	436.8491938	283.7935017	388.7211674	1.210084369	1.15E-14	http://www.ncbi.nlm.nih.gov/nuccore/NM_002421.3
NM_144990.3	mRNA	SLFNL1	protein_coding	5.634553464	6.445870437	3.055247588	9.544437362	13.20524767	12.73807237	1.214236982	0.007089824	http://www.ncbi.nlm.nih.gov/nuccore/NM_144990.3
NM_000960.3	mRNA	PTGIR	protein_coding	5.69398224	4.299449819	0.0113928	12.52724288	7.122757951	4.388884024	1.216774265	0.042367507	http://www.ncbi.nlm.nih.gov/nuccore/NM_000960.3
NM_001010917.2	mRNA	GOLGA7B	protein_coding	3.733275848	4.325833673	4.057096147	9.491461983	8.143685482	10.94683993	1.219184032	0.015227987	http://www.ncbi.nlm.nih.gov/nuccore/NM_001010917.2
NM_021044.3	mRNA	DHH	protein_coding	0.87954144	4.285716094	4.050100046	4.287631783	10.1424877	7.253784952	1.22476251	0.039338802	http://www.ncbi.nlm.nih.gov/nuccore/NM_021044.3
NM_002422.4	mRNA	MMP3	protein_coding	47.89158352	41.20848704	40.57642819	103.2695578	92.54639531	110.0431906	1.234637497	2.50E-12	http://www.ncbi.nlm.nih.gov/nuccore/NM_002422.4
NM_001185056.1	mRNA	CLDN11	protein_coding	4.711519618	5.351713636	1.029029737	15.62996698	6.120258236	5.291965124	1.251074026	0.031811445	http://www.ncbi.nlm.nih.gov/nuccore/NM_001185056.1
NM_005103.4	mRNA	FEZ1	protein_coding	5.694035226	1.157611948	3.045286978	6.37253804	9.142856276	9.108156735	1.264960637	0.022462269	http://www.ncbi.nlm.nih.gov/nuccore/NM_005103.4
NM_001347825.1	mRNA	OGDHL	protein_coding	7.548452942	5.430377212	4.067479083	20.90041027	10.1920824	10.75644977	1.266209124	0.004260549	http://www.ncbi.nlm.nih.gov/nuccore/NM_001347825.1
NM_001347824.1	mRNA	OGDHL	protein_coding	7.548452942	5.430377212	4.067479083	20.90041027	10.1920824	10.75644977	1.266210218	0.004261259	http://www.ncbi.nlm.nih.gov/nuccore/NM_001347824.1
NM_001347823.1	mRNA	OGDHL	protein_coding	7.548452942	5.430377212	4.067479083	20.90041027	10.1920824	10.75644977	1.266211109	0.004261837	http://www.ncbi.nlm.nih.gov/nuccore/NM_001347823.1
NM_001010877.3	mRNA	ZNF311	protein_coding	3.723081207	7.454589628	2.042619549	9.529410713	5.123603555	18.4900702	1.31368039	0.018118865	http://www.ncbi.nlm.nih.gov/nuccore/NM_001010877.3

NM_001318535.1	mRNA	ZNF311	protein_coding	3.723081207	7.454589628	2.042619549	9.529410713	5.123603555	18.4900702	1.313684721	0.018113245	http://www.ncbi.nlm.nih.gov/nuccore/NM_001318535.1
NM_000640.2	mRNA	IL13RA2	protein_coding	17.84598248	18.32819005	12.19286653	56.55549808	29.54494682	35.20673513	1.318922392	1.33E-05	http://www.ncbi.nlm.nih.gov/nuccore/NM_000640.2
NM_020820.3	mRNA	PREX1	protein_coding	4.711589206	1.166450065	5.061667299	7.432093245	6.124382014	14.7607778	1.337239722	0.017363091	http://www.ncbi.nlm.nih.gov/nuccore/NM_020820.3
NM_001290268.1	mRNA	FAM65C	protein_coding	8.518060751	3.336707019	5.07536802	14.75397797	21.29379042	7.905638511	1.339265736	0.003279391	http://www.ncbi.nlm.nih.gov/nuccore/NM_001290268.1
NM_018245.2	mRNA	OGDHL	protein_coding	8.505846839	5.442382416	4.069409566	22.9991365	13.23201145	10.69136188	1.349169573	0.001700722	http://www.ncbi.nlm.nih.gov/nuccore/NM_018245.2
NM_001347821.1	mRNA	OGDHL	protein_coding	8.505846839	5.442382416	4.069409566	22.9991365	13.23201145	10.69136188	1.349170977	0.001701081	http://www.ncbi.nlm.nih.gov/nuccore/NM_001347821.1
NM_178826.3	mRNA	ANO4	protein_coding	4.761745326	1.129700806	1.022637241	6.326604239	8.118169827	4.454155341	1.371885783	0.036756047	http://www.ncbi.nlm.nih.gov/nuccore/NM_178826.3
NM_001286615.1	mRNA	ANO4	protein_coding	4.761745326	1.129700806	1.022637241	6.326604239	8.118169827	4.454155341	1.371887081	0.036758409	http://www.ncbi.nlm.nih.gov/nuccore/NM_001286615.1
NM_001347819.1	mRNA	OGDHL	protein_coding	8.505846839	5.442382416	4.069409566	23.00823443	13.2342469	11.62164069	1.377838168	0.001123531	http://www.ncbi.nlm.nih.gov/nuccore/NM_001347819.1
NM_153347.2	mRNA	TMEM86A	protein_coding	10.39670159	4.439123964	7.10115321	14.86796329	17.29868546	26.61341255	1.401831403	0.000276489	http://www.ncbi.nlm.nih.gov/nuccore/NM_153347.2
NM_014370.3	mRNA	SRPK3	protein_coding	3.733275848	4.325833673	4.057096147	10.55181903	12.18897083	9.935997516	1.404798262	0.00365836	http://www.ncbi.nlm.nih.gov/nuccore/NM_014370.3
NM_001347830.1	mRNA	PDGFRA	protein_coding	17.80513837	20.46427366	14.21697661	48.44258763	68.97091089	22.84245114	1.414245215	0.000134959	http://www.ncbi.nlm.nih.gov/nuccore/NM_001347830.1
NM_001347829.1	mRNA	PDGFRA	protein_coding	17.80513837	20.46427366	14.21697661	48.44258763	68.97091089	22.84245114	1.414245711	0.000135036	http://www.ncbi.nlm.nih.gov/nuccore/NM_001347829.1
NM_006206.5	mRNA	PDGFRA	protein_coding	17.80513837	20.46427366	14.21697661	49.48205739	68.97355457	22.83377944	1.424873877	0.00012113	http://www.ncbi.nlm.nih.gov/nuccore/NM_006206.5
NM_001286616.1	mRNA	ANO4	protein_coding	3.802645173	1.119772469	1.020911363	5.282316319	8.11243422	3.537202383	1.42497297	0.043235042	http://www.ncbi.nlm.nih.gov/nuccore/NM_001286616.1
NM_001168247.2	mRNA	SLFNL1	protein_coding	7.536810658	7.52594229	3.060651094	14.79855986	15.25730883	19.17022458	1.426242704	0.000467209	http://www.ncbi.nlm.nih.gov/nuccore/NM_001168247.2
NM_001347828.1	mRNA	PDGFRA	protein_coding	17.80513837	20.46427366	14.21697661	49.48870649	68.97609035	23.75755367	1.434051233	7.78E-05	http://www.ncbi.nlm.nih.gov/nuccore/NM_001347828.1
NM_032291.3	mRNA	SGIP1	protein_coding	3.789750447	2.178781536	1.022640995	7.362421685	8.120953998	4.440601054	1.443190318	0.024539443	http://www.ncbi.nlm.nih.gov/nuccore/NM_032291.3
NM_001347822.1	mRNA	OGDHL	protein_coding	6.591321776	5.418102705	4.065505771	21.95439042	12.21800887	10.71695421	1.450282838	0.000935263	http://www.ncbi.nlm.nih.gov/nuccore/NM_001347822.1
NM_001143997.1	mRNA	OGDHL	protein_coding	6.591321776	5.418102705	4.065505771	21.95439042	12.21800887	10.71695421	1.450285247	0.00093571	http://www.ncbi.nlm.nih.gov/nuccore/NM_001143997.1
NM_001347820.1	mRNA	OGDHL	protein_coding	6.591321776	5.418102705	4.065505771	22.98983754	12.22025246	10.70406511	1.482949987	0.000784758	http://www.ncbi.nlm.nih.gov/nuccore/NM_001347820.1
NM_001143996.1	mRNA	OGDHL	protein_coding	6.591321776	5.418102705	4.065505771	22.98983754	12.22025246	10.70406511	1.482952161	0.000785101	http://www.ncbi.nlm.nih.gov/nuccore/NM_001143996.1
NM_001300859.1	mRNA	SLFNL1	protein_coding	3.733258998	5.365793614	3.049453318	10.56709862	11.18523196	12.75358366	1.486965662	0.001761152	http://www.ncbi.nlm.nih.gov/nuccore/NM_001300859.1
NM_001142800.1	mRNA	EYS	protein_coding	1.874370665	3.190549918	0.00576146	4.237713539	7.098866545	3.564236337	1.490815369	0.049883798	http://www.ncbi.nlm.nih.gov/nuccore/NM_001142800.1
NM_001292009.1	mRNA	EYS	protein_coding	1.874370665	3.190549918	0.00576146	4.237713539	7.098866545	3.564236337	1.490815566	0.049887087	http://www.ncbi.nlm.nih.gov/nuccore/NM_001292009.1
NM_001017961.4	mRNA	FAM78B	protein_coding	3.789762536	1.129713006	2.031294027	3.246088644	4.087149399	12.99951806	1.495630831	0.039128935	http://www.ncbi.nlm.nih.gov/nuccore/NM_001017961.4
NM_000739.2	mRNA	CHRM2	protein_coding	0.90270767	5.273115517	0.006901879	6.318422696	8.115342602	3.524132402	1.500609563	0.032319699	http://www.ncbi.nlm.nih.gov/nuccore/NM_000739.2
NM_001006626.1	mRNA	CHRM2	protein_coding	0.90270767	5.273115517	0.006901879	6.318422696	8.115342602	3.524132402	1.500610053	0.032313367	http://www.ncbi.nlm.nih.gov/nuccore/NM_001006626.1
NM_001006627.1	mRNA	CHRM2	protein_coding	0.90270767	5.273115517	0.006901879	6.318422696	8.115342602	3.524132402	1.500610379	0.032309156	http://www.ncbi.nlm.nih.gov/nuccore/NM_001006627.1
NM_001006628.1	mRNA	CHRM2	protein_coding	0.90270767	5.273115517	0.006901879	6.318422696	8.115342602	3.524132402	1.500610721	0.032304724	http://www.ncbi.nlm.nih.gov/nuccore/NM_001006628.1
NM_001006629.1	mRNA	CHRM2	protein_coding	0.90270767	5.273115517	0.006901879	6.318422696	8.115342602	3.524132402	1.500611035	0.032300658	http://www.ncbi.nlm.nih.gov/nuccore/NM_001006629.1
NM_001006630.1	mRNA	CHRM2	protein_coding	0.90270767	5.273115517	0.006901879	6.318422696	8.115342602	3.524132402	1.500611067	0.032300245	http://www.ncbi.nlm.nih.gov/nuccore/NM_001006630.1
NM_001006631.1	mRNA	CHRM2	protein_coding	0.90270767	5.273115517	0.006901879	6.318422696	8.115342602	3.524132402	1.500611536	0.032294175	http://www.ncbi.nlm.nih.gov/nuccore/NM_001006631.1
NM_001006632.1	mRNA	CHRM2	protein_coding	0.90270767	5.273115517	0.006901879	6.318422696	8.115342602	3.524132402	1.500611724	0.032291737	http://www.ncbi.nlm.nih.gov/nuccore/NM_001006632.1
NM_001178063.2	mRNA	C2	protein_coding	2.831584335	1.119783366	2.029154438	4.259930836	7.107300541	6.36561984	1.501104238	0.027599397	http://www.ncbi.nlm.nih.gov/nuccore/NM_001178063.2
NM_052840.4	mRNA	CELF6	protein_coding	2.831584335	1.119783366	2.029154438	9.395638668	5.090190701	3.524080757	1.50673721	0.029733078	http://www.ncbi.nlm.nih.gov/nuccore/NM_052840.4
NM_006475.2	mRNA	POSTN	protein_coding	2.820051867	2.178796567	2.031298663	5.312746881	10.13960539	5.371973282	1.510351825	0.01687242	http://www.ncbi.nlm.nih.gov/nuccore/NM_006475.2
NM_001330517.1	mRNA	POSTN	protein_coding	2.820051867	2.178796567	2.031298663	5.312746881	10.13960539	5.371973282	1.510351894	0.016872641	http://www.ncbi.nlm.nih.gov/nuccore/NM_001330517.1
NM_001286665.1	mRNA	POSTN	protein_coding	2.820051867	2.178796567	2.031298663	5.312746881	10.13960539	5.371973282	1.510352675	0.016875169	http://www.ncbi.nlm.nih.gov/nuccore/NM_001286665.1
NM_001135935.1	mRNA	POSTN	protein_coding	2.820051867	2.178796567	2.031298663	5.312746881	10.13960539	5.371973282	1.510353013	0.016876265	http://www.ncbi.nlm.nih.gov/nuccore/NM_001135935.1
NM_173644.2	mRNA	C20orf197	protein_coding	3.777498335	3.233149965	1.024309418	7.394767701	8.131485237	8.173017064	1.514754943	0.009364052	http://www.ncbi.nlm.nih.gov/nuccore/NM_173644.2
NM_152701.4	mRNA	ABCA13	protein_coding	1.85394508	4.25628074	1.02264851	3.252396357	9.134489072	9.157361171	1.568665496	0.012780287	http://www.ncbi.nlm.nih.gov/nuccore/NM_152701.4
NM_001105521.3	mRNA	JAKMIP3	protein_coding	3.754544279	5.337223835	1.027499799	14.64353706	8.148399698	8.070412447	1.572425455	0.002886178	http://www.ncbi.nlm.nih.gov/nuccore/NM_001105521.3
NM_001143818.1	mRNA	SERPINB2	protein_coding	1.85394508	4.25628074	1.02264851	13.53104177	1.051319278	7.253561269	1.574217456	0.036128931	http://www.ncbi.nlm.nih.gov/nuccore/NM_001143818.1
NM_002575.2	mRNA	SERPINB2	protein_coding	1.85394508	4.25628074	1.02264851	13.53104177	1.051319278	7.253561269	1.574218212	0.036115765	http://www.ncbi.nlm.nih.gov/nuccore/NM_002575.2
NM_005602.5	mRNA	CLDN11	protein_coding	4.711519618	5.351713636	1.029029737	17.74808994	8.155137114	8.02973838	1.575634822	0.002704038	http://www.ncbi.nlm.nih.gov/nuccore/NM_005602.5
NM_001286666.1	mRNA	POSTN	protein_coding	2.831584335	1.119783366	2.029154438	5.297802108	8.118152659	5.400631539	1.578939145	0.018377375	http://www.ncbi.nlm.nih.gov/nuccore/NM_001286666.1
NM_001286667.1	mRNA	POSTN	protein_coding	2.831584335	1.119783366	2.029154438	5.297802108	8.118152659	5.400631539	1.578940196	0.018380185	http://www.ncbi.nlm.nih.gov/nuccore/NM_001286667.1
NM_181486.2	mRNA	TBX5	protein_coding	0.894733294	2.178826643	4.044950892	4.287776691	14.1724422	3.474515323	1.579611103	0.023908233	http://www.ncbi.nlm.nih.gov/nuccore/NM_181486.2
NM_080717.2	mRNA	TBX5	protein_coding	0.894733294	2.178826643	4.044950892	4.287776691	14.1724422	3.474515323	1.57961201	0.02391627	http://www.ncbi.nlm.nih.gov/nuccore/NM_080717.2
NM_000192.3	mRNA	TBX5	protein_coding	0.894733294	2.178826643	4.044950892	4.287776691	14.1724422	3.474515323	1.579613028	0.023925287	http://www.ncbi.nlm.nih.gov/nuccore/NM_000192.3
NM_002113.2	mRNA	CFHR1	protein_coding	3.765816735	2.201877277	3.043119061	8.462450816	9.15272218	10.94687205	1.620542903	0.002487294	http://www.ncbi.nlm.nih.gov/nuccore/NM_002113.2
NM_001302815.1	mRNA	C20orf197	protein_coding	1.863890576	3.205496055	1.020918074	6.334499208	5.094838567	8.23764066	1.647677407	0.012159365	http://www.ncbi.nlm.nih.gov/nuccore/NM_001302815.1
NM_152353.2	mRNA	CLDND2	protein_coding	1.863899015	2.166518597	2.029158616	8.3889495	6.104008029	5.386090396	1.651285748	0.011968835	http://www.ncbi.nlm.nih.gov/nuccore/NM_152353.2
NM_001135934.1	mRNA	POSTN	protein_coding	2.831584335	1.119783366	2.029154438	5.305345509	9.128907542	5.386153776	1.65188459	0.01239123	http://www.ncbi.nlm.nih.gov/nuccore/NM_001135934.1
NM_001135936.1	mRNA	POSTN	protein_coding	2.831584335	1.119783366	2.029154438	5.305345509	9.128907542	5.386153776	1.651885426	0.012392754	http://www.ncbi.nlm.nih.gov/nuccore/NM_001135936.1
NM_001170760.1	mRNA	SRPK3	protein_coding	3.754589525	2.212826826	4.052499274	10.55181903	12.18897083	9.935997516	1.660040278	0.000997489	http://www.ncbi.nlm.nih.gov/nuccore/NM_001170760.1
NM_152479.5	mRNA	TTC9B	protein_coding	1.874370665	3.190549918	0.00576146	7.336251673	4.078510791	5.430526572	1.66651253	0.021437359	http://www.ncbi.nlm.nih.gov/nuccore/NM_152479.5
NM_004418.3	mRNA	DUSP2	protein_coding	4.748363137	2.190533098	1.024305277	6.386903288	11.1645612	9.076878291	1.683001425	0.003076406	http://www.ncbi.nlm.nih.gov/nuccore/NM_004418.3
NM_001135195.1	mRNA	SLC39A5	protein_coding	0	1.098539577	3.030619889	2.16592839	5.080064195	6.432505209	1.684964965	0.038684007	http://www.ncbi.nlm.nih.gov/nuccore/NM_001135195.1
NM_173596.2	mRNA	SLC39A5	protein_coding	0	1.098539577	3.030619889	2.16592839	5.080064195	6.432505209	1.684967775	0.038679304	http://www.ncbi.nlm.nih.gov/nuccore/NM_173596.2
NM_001288811.1	mRNA	ARL17A	protein_coding	2.856832771	1.098515344	0.004617101	5.257624971	5.080100319	3.578241085	1.693285763	0.036547839	http://www.ncbi.nlm.nih.gov/nuccore/NM_001288811.1
NM_001302813.1	mRNA	C20orf197	protein_coding	3.789750447	2.178781536	1.022640995	7.394767701	8.131485237	8.173017064	1.698801297	0.004642357	http://www.ncbi.nlm.nih.gov/nuccore/NM_001302813.1
NM_001160100.1	mRNA	CLDN10	protein_coding	1.863890576	3.205496055	1.020918074	4.280779084	6.106373177	10.12903074	1.713531642	0.008744308	http://www.ncbi.nlm.nih.gov/nuccore/NM_001160100.1
NM_006984.4	mRNA	CLDN10	protein_coding	1.863890576	3.205496055	1.020918074	4.280779084	6.106373177	10.12903074	1.713533534	0.008743092	http://www.ncbi.nlm.nih.gov/nuccore/NM_006984.4
NM_006182.2	mRNA	DDR2	protein_coding	10.4599016	4.385428989	2.049192459	14.84896198	29.39656593	12.45188876	1.714616624	0.00033605	http://www.ncbi.nlm.nih.gov/nuccore/NM_006182.2

NM_001308203.1	mRNA	SGIP1	protein_coding	2.831574534	2.166505065	1.020914718	9.423852548	8.123700369	3.486484716	1.72472504	0.008817695	http://www.ncbi.nlm.nih.gov/nuccore/NM_001308203.1
NM_001014796.1	mRNA	DDR2	protein_coding	10.4599016	4.385428989	2.049192459	14.85581165	30.40767752	12.43983417	1.740884637	0.000344534	http://www.ncbi.nlm.nih.gov/nuccore/NM_001014796.1
NM_001282773.1	mRNA	RGS7	protein_coding	1.87437813	2.153592464	1.019112785	8.370770228	3.07052198	6.36553517	1.747411673	0.014275341	http://www.ncbi.nlm.nih.gov/nuccore/NM_001282773.1
NM_005814.2	mRNA	GPA33	protein_coding	0.919872005	2.139886464	1.017215633	4.237655846	5.08268963	5.461995223	1.785868919	0.022837187	http://www.ncbi.nlm.nih.gov/nuccore/NM_005814.2
NM_001204963.1	mRNA	PBX1	protein_coding	0	2.139896681	2.024507633	5.266024701	5.082702175	4.511427168	1.786355487	0.023004445	http://www.ncbi.nlm.nih.gov/nuccore/NM_001204963.1
NM_001145903.2	mRNA	C2	protein_coding	1.874385594	1.109425529	2.026900837	4.267026391	7.109983428	7.301393459	1.822590558	0.008652505	http://www.ncbi.nlm.nih.gov/nuccore/NM_001145903.2
NM_000063.5	mRNA	C2	protein_coding	1.874385594	1.109425529	2.026900837	4.267026391	7.109983428	7.301393459	1.822590565	0.008652488	http://www.ncbi.nlm.nih.gov/nuccore/NM_000063.5
NM_001282458.1	mRNA	C2	protein_coding	1.874385594	1.109425529	2.026900837	4.267026391	7.109983428	7.301393459	1.822590938	0.008651629	http://www.ncbi.nlm.nih.gov/nuccore/NM_001282458.1
NM_182848.3	mRNA	CLDN10	protein_coding	1.863890576	3.205496055	1.020918074	4.294081433	6.111093281	12.00400798	1.839280693	0.006489625	http://www.ncbi.nlm.nih.gov/nuccore/NM_182848.3
NM_018417.5	mRNA	ADCY10	protein_coding	0.929260583	2.12514441	0.00347209	5.240037871	2.047505023	4.558450504	1.84766134	0.041584	http://www.ncbi.nlm.nih.gov/nuccore/NM_018417.5
NM_001297772.1	mRNA	ADCY10	protein_coding	0.929260583	2.12514441	0.00347209	5.240037871	2.047505023	4.558450504	1.847662589	0.04157756	http://www.ncbi.nlm.nih.gov/nuccore/NM_001297772.1
NM_001288813.1	mRNA	ARL17A	protein_coding	2.870992259	0.019455788	0.003469888	5.240065172	3.057526767	3.607475433	1.853007142	0.041790991	http://www.ncbi.nlm.nih.gov/nuccore/NM_001288813.1
NM_001323086.1	mRNA	JAKMIP3	protein_coding	3.789738356	3.219629335	0.008028351	11.5294311	7.129793702	8.127552593	1.867654516	0.001438522	http://www.ncbi.nlm.nih.gov/nuccore/NM_001323086.1
NM_152426.3	mRNA	APOBEC3D	protein_coding	0.91987703	1.098531497	2.024504469	2.178300524	6.093672335	7.352377448	1.873799995	0.015861595	http://www.ncbi.nlm.nih.gov/nuccore/NM_152426.3
NM_001136475.2	mRNA	VASH2	protein_coding	0.919872005	2.139886464	1.017215633	5.274248446	6.093714141	4.496596875	1.878268071	0.014427478	http://www.ncbi.nlm.nih.gov/nuccore/NM_001136475.2
NM_001170761.1	mRNA	SRPK3	protein_coding	1.844473599	2.190582472	4.047575171	9.507272103	12.18370011	9.017270112	1.879284908	0.000513569	http://www.ncbi.nlm.nih.gov/nuccore/NM_001170761.1
NM_005091.2	mRNA	PGLYRP1	protein_coding	1.885555052	0.025892007	2.024501306	5.274313351	8.109472647	2.609258125	1.883929053	0.016136897	http://www.ncbi.nlm.nih.gov/nuccore/NM_005091.2
NM_001282775.1	mRNA	RGS7	protein_coding	1.863890576	3.205496055	1.020918074	11.5020381	3.082067549	9.124079165	1.906909685	0.003104298	http://www.ncbi.nlm.nih.gov/nuccore/NM_001282775.1
NM_001282778.1	mRNA	RGS7	protein_coding	1.863890576	3.205496055	1.020918074	11.5020381	3.082067549	9.124079165	1.906910205	0.003103539	http://www.ncbi.nlm.nih.gov/nuccore/NM_001282778.1
NM_002924.5	mRNA	RGS7	protein_coding	1.863890576	3.205496055	1.020918074	11.5020381	3.082067549	9.124079165	1.90691038	0.003103284	http://www.ncbi.nlm.nih.gov/nuccore/NM_002924.5
NM_001323087.1	mRNA	JAKMIP3	protein_coding	3.789738356	3.219629335	0.008028351	11.53825118	8.141373426	8.112946535	1.919499304	0.000904051	http://www.ncbi.nlm.nih.gov/nuccore/NM_001323087.1
NM_001323090.1	mRNA	JAKMIP3	protein_coding	3.789738356	3.219629335	0.008028351	11.53825118	8.141373426	8.112946535	1.91949992	0.000904208	http://www.ncbi.nlm.nih.gov/nuccore/NM_001323090.1
NM_001323088.1	mRNA	JAKMIP3	protein_coding	3.789738356	3.219629335	0.008028351	11.53825118	8.141373426	8.112946535	1.919500287	0.000904301	http://www.ncbi.nlm.nih.gov/nuccore/NM_001323088.1
NM_006258.3	mRNA	PRKG1	protein_coding	0	3.157495919	0.003473192	2.159570917	6.085311085	4.542358902	1.955882571	0.027568538	http://www.ncbi.nlm.nih.gov/nuccore/NM_006258.3
NM_153619.1	mRNA	SEMA6D	protein_coding	1.897614494	0.019459277	1.015189663	5.249026233	6.085348149	1.708866257	1.967739246	0.026802442	http://www.ncbi.nlm.nih.gov/nuccore/NM_153619.1
NM_153618.1	mRNA	SEMA6D	protein_coding	1.897614494	0.019459277	1.015189663	5.249026233	6.085348149	1.708866257	1.967743504	0.026806533	http://www.ncbi.nlm.nih.gov/nuccore/NM_153618.1
NM_153617.1	mRNA	SEMA6D	protein_coding	1.897614494	0.019459277	1.015189663	5.249026233	6.085348149	1.708866257	1.967743821	0.026806837	http://www.ncbi.nlm.nih.gov/nuccore/NM_153617.1
NM_153616.1	mRNA	SEMA6D	protein_coding	1.897614494	0.019459277	1.015189663	5.249026233	6.085348149	1.708866257	1.967747192	0.026810076	http://www.ncbi.nlm.nih.gov/nuccore/NM_153616.1
NM_020858.1	mRNA	SEMA6D	protein_coding	1.897614494	0.019459277	1.015189663	5.249026233	6.085348149	1.708866257	1.967749888	0.026812666	http://www.ncbi.nlm.nih.gov/nuccore/NM_020858.1
NM_001198999.1	mRNA	SEMA6D	protein_coding	1.897614494	0.019459277	1.015189663	5.249026233	6.085348149	1.708866257	1.967753357	0.026816001	http://www.ncbi.nlm.nih.gov/nuccore/NM_001198999.1
NM_001323089.1	mRNA	JAKMIP3	protein_coding	2.83156473	3.205480432	0.006897557	10.48555233	8.134058195	6.262026046	1.968974048	0.001453467	http://www.ncbi.nlm.nih.gov/nuccore/NM_001323089.1
NM_000623.3	mRNA	BDKRB2	protein_coding	0	3.190577578	2.026908221	9.423994471	11.1473278	0.708532271	1.980722881	0.015238916	http://www.ncbi.nlm.nih.gov/nuccore/NM_000623.3
NM_001172684.1	mRNA	CELF6	protein_coding	0.91987703	1.098531497	2.024504469	9.395638668	5.090190701	3.524080757	2.04878928	0.006397442	http://www.ncbi.nlm.nih.gov/nuccore/NM_001172684.1
NM_032546.3	mRNA	TRIM54	protein_coding	0	1.074382421	1.012997377	2.139394976	4.06111098	3.638648063	2.100310185	0.043836715	http://www.ncbi.nlm.nih.gov/nuccore/NM_032546.3
NM_187841.2	mRNA	TRIM54	protein_coding	0	1.074382421	1.012997377	2.139394976	4.06111098	3.638648063	2.100310799	0.043835041	http://www.ncbi.nlm.nih.gov/nuccore/NM_187841.2
NM_001291447.1	mRNA	SALL2	protein_coding	0.939485257	0.01300215	1.012995901	3.169618203	4.061120019	2.688832706	2.103927477	0.043535955	http://www.ncbi.nlm.nih.gov/nuccore/NM_001291447.1
NM_145654.3	mRNA	RDM1	protein_coding	0.939482265	1.074377658	0.002319462	3.169658765	6.076178597	0.812849959	2.108590465	0.048373455	http://www.ncbi.nlm.nih.gov/nuccore/NM_145654.3
NM_000599.3	mRNA	IGFBP5	protein_coding	84.24218841	108.787053	71.05382476	449.5043146	563.6970356	137.0843094	2.122945624	7.41E-09	http://www.ncbi.nlm.nih.gov/nuccore/NM_000599.3
NM_001010932.2	mRNA	HGF	protein_coding	4.70015175	4.32580661	3.049445762	33.34550425	17.29040088	5.911031592	2.197307132	0.000185722	http://www.ncbi.nlm.nih.gov/nuccore/NM_001010932.2
NM_000601.5	mRNA	HGF	protein_coding	4.70015175	4.32580661	3.049445762	33.35556434	18.30201543	5.901553885	2.222605016	0.00014551	http://www.ncbi.nlm.nih.gov/nuccore/NM_000601.5
NM_001166395.1	mRNA	CHST4	protein_coding	0.939485257	0.01300215	1.012995901	4.205222685	1.033710254	5.530413103	2.23268074	0.02833246	http://www.ncbi.nlm.nih.gov/nuccore/NM_001166395.1
NM_016124.4	mRNA	RHD	protein_coding	1.910916478	0.012999808	0.002318723	4.205270357	3.055156154	3.622790834	2.237123821	0.02631138	http://www.ncbi.nlm.nih.gov/nuccore/NM_016124.4
NM_001282867.1	mRNA	RHD	protein_coding	1.910916478	0.012999808	0.002318723	4.205270357	3.055156154	3.622790834	2.237124446	0.026312628	http://www.ncbi.nlm.nih.gov/nuccore/NM_001282867.1
NM_001098512.2	mRNA	PRKG1	protein_coding	0	3.157495919	0.003473192	5.282249082	6.096363055	5.430565196	2.33632733	0.003535874	http://www.ncbi.nlm.nih.gov/nuccore/NM_001098512.2
NM_001145291.1	mRNA	PDE6B	protein_coding	0.939485257	0.01300215	1.012995901	2.152996304	4.066422769	5.512522585	2.354355739	0.015931391	http://www.ncbi.nlm.nih.gov/nuccore/NM_001145291.1
NM_001320901.1	mRNA	FHIT	protein_coding	0.939485257	0.01300215	1.012995901	1.114353769	6.082350379	4.558509431	2.354933559	0.017267178	http://www.ncbi.nlm.nih.gov/nuccore/NM_001320901.1
NM_001172685.1	mRNA	CELF6	protein_coding	0	1.074382421	1.012997377	4.213752221	5.074663528	2.660788932	2.35744081	0.015505264	http://www.ncbi.nlm.nih.gov/nuccore/NM_001172685.1
NM_153840.3	mRNA	ADGRF1	protein_coding	0.939482265	1.074377658	0.002319462	4.221990531	7.092867668	1.708877561	2.473529581	0.010227148	http://www.ncbi.nlm.nih.gov/nuccore/NM_153840.3
NM_005547.2	mRNA	IVL	protein_coding	1.863907452	1.119794267	3.036023977	31.07702134	4.115707437	1.485247043	2.552450234	0.0090393	http://www.ncbi.nlm.nih.gov/nuccore/NM_005547.2
NM_002256.3	mRNA	KISS1	protein_coding	0.939485257	0.01300215	1.012995901	3.199747326	4.071431404	6.43248681	2.570513269	0.005452228	http://www.ncbi.nlm.nih.gov/nuccore/NM_002256.3
NM_001322799.1	mRNA	KCNS1	protein_coding	0	0.006515795	1.010542854	1.091092034	3.047555606	3.672218611	2.580371062	0.044693954	http://www.ncbi.nlm.nih.gov/nuccore/NM_001322799.1
NM_002251.4	mRNA	KCNS1	protein_coding	0	0.006515795	1.010542854	1.091092034	3.047555606	3.672218611	2.580372771	0.044690622	http://www.ncbi.nlm.nih.gov/nuccore/NM_002251.4
NM_001282464.1	mRNA	C14orf132	protein_coding	0	1.060405691	0.00116248	3.153027169	4.055408693	0.834327402	2.586543831	0.04521773	http://www.ncbi.nlm.nih.gov/nuccore/NM_001282464.1
NM_021637.2	mRNA	TMEM35A	protein_coding	0.950966215	0.006514617	0.001162108	3.153027169	4.055408693	0.834327402	2.589512316	0.045253332	http://www.ncbi.nlm.nih.gov/nuccore/NM_021637.2
NM_019060.2	mRNA	CRCT1	protein_coding	0	1.074382421	1.012997377	3.213621344	2.055295379	10.22387583	2.739978193	0.00355639	http://www.ncbi.nlm.nih.gov/nuccore/NM_019060.2
NM_001134663.1	mRNA	SAMD13	protein_coding	0.950966215	0.006514617	0.001162108	0.037696632	4.058295541	4.610388305	2.740252105	0.029287588	http://www.ncbi.nlm.nih.gov/nuccore/NM_001134663.1
NM_001134664.1	mRNA	SAMD13	protein_coding	0.950966215	0.006514617	0.001162108	0.037696632	4.058295541	4.610388305	2.74025566	0.029281183	http://www.ncbi.nlm.nih.gov/nuccore/NM_001134664.1
NM_001034836.1	mRNA	RDM1	protein_coding	0	1.060405691	0.00116248	2.132312687	6.072898371	0.82344694	2.750381624	0.029131539	http://www.ncbi.nlm.nih.gov/nuccore/NM_001034836.1
NM_032883.2	mRNA	TOX2	protein_coding	0	0.006515795	1.010542854	3.161497257	5.065831776	0.823439658	2.751419997	0.02653939	http://www.ncbi.nlm.nih.gov/nuccore/NM_032883.2
NM_005182.2	mRNA	CA7	protein_coding	0	0.006515795	1.010542854	3.161497257	5.065831776	0.823439658	2.751423626	0.026543679	http://www.ncbi.nlm.nih.gov/nuccore/NM_005182.2
NM_001098797.1	mRNA	TOX2	protein_coding	0	0.006515795	1.010542854	3.161497257	5.065831776	0.823439658	2.751425805	0.026546253	http://www.ncbi.nlm.nih.gov/nuccore/NM_001098797.1
NM_001098798.1	mRNA	TOX2	protein_coding	0	0.006515795	1.010542854	3.161497257	5.065831776	0.823439658	2.751427258	0.02654797	http://www.ncbi.nlm.nih.gov/nuccore/NM_001098798.1
NM_001098796.1	mRNA	TOX2	protein_coding	0	0.006515795	1.010542854	3.161497257	5.065831776	0.823439658	2.751428784	0.026549775	http://www.ncbi.nlm.nih.gov/nuccore/NM_001098796.1
NM_001014435.1	mRNA	CA7	protein_coding	0	0.006515795	1.010542854	3.161497257	5.065831776	0.823439658	2.751430742	0.02655209	http://www.ncbi.nlm.nih.gov/nuccore/NM_001014435.1
NM_144690.2	mRNA	ZNF582	protein_coding	0	0.006515795	1.010542854	5.211641009	3.050204615	0.823425089	2.752997596	0.026707993	http://www.ncbi.nlm.nih.gov/nuccore/NM_144690.2
NM_001320371.1	mRNA	ZNF582	protein_coding	0	0.006515795	1.010542854	5.211641009	3.050204615	0.823425089	2.75300077	0.026710957	http://www.ncbi.nlm.nih.gov/nuccore/NM_001320371.1

NM_024780.4	mRNA	TMC5	protein_coding	0	0.006515795	1.010542854	6.234341441	2.041100233	0.823417804	2.756970689	0.029704966	http://www.ncbi.nlm.nih.gov/nuccore/NM_024780.4
NM_001308161.1	mRNA	TMC5	protein_coding	0	0.006515795	1.010542854	6.234341441	2.041100233	0.823417804	2.756974818	0.029709121	http://www.ncbi.nlm.nih.gov/nuccore/NM_001308161.1
NM_001261841.1	mRNA	TMC5	protein_coding	0	0.006515795	1.010542854	6.234341441	2.041100233	0.823417804	2.75698124	0.029715586	http://www.ncbi.nlm.nih.gov/nuccore/NM_001261841.1
NM_001105249.1	mRNA	TMC5	protein_coding	0	0.006515795	1.010542854	6.234341441	2.041100233	0.823417804	2.756987921	0.029722314	http://www.ncbi.nlm.nih.gov/nuccore/NM_001105249.1
NM_001105248.1	mRNA	TMC5	protein_coding	0	0.006515795	1.010542854	6.234341441	2.041100233	0.823417804	2.756990119	0.029724528	http://www.ncbi.nlm.nih.gov/nuccore/NM_001105248.1
NM_000283.3	mRNA	PDE6B	protein_coding	0.939485257	0.01300215	1.012995901	4.245226577	5.085225477	6.39817875	2.759648222	0.001767946	http://www.ncbi.nlm.nih.gov/nuccore/NM_000283.3
NM_001145292.1	mRNA	PDE6B	protein_coding	0.939485257	0.01300215	1.012995901	4.245226577	5.085225477	6.39817875	2.759648714	0.001767633	http://www.ncbi.nlm.nih.gov/nuccore/NM_001145292.1
NM_001451.2	mRNA	FOXF1	protein_coding	2.831594135	0.038671066	3.036019141	15.78293453	23.30988568	5.100233882	2.801372422	1.69E-05	http://www.ncbi.nlm.nih.gov/nuccore/NM_001451.2
NM_173565.3	mRNA	RSPH10B	protein_coding	0	0.006515795	1.010542854	0.041822158	7.083104356	2.688865396	2.889936115	0.024317744	http://www.ncbi.nlm.nih.gov/nuccore/NM_173565.3
NM_152770.2	mRNA	C4orf22	protein_coding	0	1.060405691	0.00116248	3.169597919	3.052711122	3.63863594	2.890968172	0.013698093	http://www.ncbi.nlm.nih.gov/nuccore/NM_152770.2
NM_001206997.1	mRNA	C4orf22	protein_coding	0	1.060405691	0.00116248	3.177486309	4.0638202	3.622803678	3.024095869	0.007642162	http://www.ncbi.nlm.nih.gov/nuccore/NM_001206997.1
NM_001024455.3	mRNA	RGAG4	protein_coding	0	1.060405691	0.00116248	4.205270357	3.055156154	3.622790834	3.024479163	0.007655931	http://www.ncbi.nlm.nih.gov/nuccore/NM_001024455.3
NM_001271733.1	mRNA	MST1L	protein_coding	0	0.006515795	1.010542854	3.18513692	5.074652541	3.607502504	3.14673428	0.004288728	http://www.ncbi.nlm.nih.gov/nuccore/NM_001271733.1
NM_005416.2	mRNA	SPRR3	protein_coding	0	1.060405691	0.00116248	0.053912361	0.016915277	12.21147183	3.169301958	0.030031268	http://www.ncbi.nlm.nih.gov/nuccore/NM_005416.2
NM_002965.3	mRNA	S100A9	protein_coding	0	0.006515795	1.010542854	4.273745398	0.025646951	14.93865472	3.809664317	0.00400615	http://www.ncbi.nlm.nih.gov/nuccore/NM_002965.3
NM_001136533.1	mRNA	DCAF8L2	protein_coding	6.94E-17	6.94E-17	6.94E-17	2.109202433	0.007938918	3.70902762	4.067123528	0.038520298	http://www.ncbi.nlm.nih.gov/nuccore/NM_001136533.1
NM_016523.2	mRNA	KLRF1	protein_coding	6.94E-17	6.94E-17	6.94E-17	0.025302071	4.049147152	1.799741315	4.072831971	0.039343504	http://www.ncbi.nlm.nih.gov/nuccore/NM_016523.2
NM_032054.1	mRNA	PCDHGA5	protein_coding	6.94E-17	6.94E-17	6.94E-17	2.10922758	2.033942835	1.799727766	4.075081284	0.034432982	http://www.ncbi.nlm.nih.gov/nuccore/NM_032054.1
NM_016943.2	mRNA	TAS2R3	protein_coding	6.94E-17	6.94E-17	6.94E-17	2.10922758	2.033942835	1.799727766	4.075081374	0.034433687	http://www.ncbi.nlm.nih.gov/nuccore/NM_016943.2
NM_133371.4	mRNA	MYOZ3	protein_coding	6.94E-17	6.94E-17	6.94E-17	3.134945484	2.033947895	0.857224154	4.078385851	0.035130475	http://www.ncbi.nlm.nih.gov/nuccore/NM_133371.4
NM_001122853.2	mRNA	MYOZ3	protein_coding	6.94E-17	6.94E-17	6.94E-17	3.134945484	2.033947895	0.857224154	4.078387061	0.03513188	http://www.ncbi.nlm.nih.gov/nuccore/NM_001122853.2
NM_170776.4	mRNA	ADGRG3	protein_coding	6.94E-17	6.94E-17	6.94E-17	4.158019511	0.007944112	1.799714214	4.078419846	0.039316081	http://www.ncbi.nlm.nih.gov/nuccore/NM_170776.4
NM_001308360.1	mRNA	ADGRG3	protein_coding	6.94E-17	6.94E-17	6.94E-17	4.158019511	0.007944112	1.799714214	4.078422451	0.039322925	http://www.ncbi.nlm.nih.gov/nuccore/NM_001308360.1
NM_198174.2	mRNA	GRHL3	protein_coding	6.94E-17	6.94E-17	6.94E-17	0.025308456	5.055832516	0.857240397	4.079295005	0.047982069	http://www.ncbi.nlm.nih.gov/nuccore/NM_198174.2
NM_001271829.1	mRNA	C9orf92	protein_coding	6.94E-17	6.94E-17	6.94E-17	1.078427737	5.05583965	0	4.087858482	0.04524133	http://www.ncbi.nlm.nih.gov/nuccore/NM_001271829.1
NM_001137560.1	mRNA	TMEM151B	protein_coding	6.94E-17	6.94E-17	6.94E-17	1.084865168	3.044823316	2.735002413	4.282970829	0.018048827	http://www.ncbi.nlm.nih.gov/nuccore/NM_001137560.1
NM_001304819.1	mRNA	TSACC	protein_coding	6.94E-17	6.94E-17	6.94E-17	2.117228186	2.036422666	2.734993711	4.283782202	0.017665725	http://www.ncbi.nlm.nih.gov/nuccore/NM_001304819.1
NM_001304820.1	mRNA	TSACC	protein_coding	6.94E-17	6.94E-17	6.94E-17	2.117228186	2.036422666	2.734993711	4.283782904	0.017664057	http://www.ncbi.nlm.nih.gov/nuccore/NM_001304820.1
NM_001304826.1	mRNA	TSACC	protein_coding	6.94E-17	6.94E-17	6.94E-17	2.117228186	2.036422666	2.734993711	4.283783236	0.01766327	http://www.ncbi.nlm.nih.gov/nuccore/NM_001304826.1
NM_001244000.1	mRNA	SPIB	protein_coding	6.94E-17	6.94E-17	6.94E-17	4.168302876	0.00924662	2.734976304	4.285089601	0.020297311	http://www.ncbi.nlm.nih.gov/nuccore/NM_001244000.1
NM_003121.4	mRNA	SPIB	protein_coding	6.94E-17	6.94E-17	6.94E-17	4.168302876	0.00924662	2.734976304	4.28508962	0.020297599	http://www.ncbi.nlm.nih.gov/nuccore/NM_003121.4
NM_001243999.1	mRNA	SPIB	protein_coding	6.94E-17	6.94E-17	6.94E-17	4.168302876	0.00924662	2.734976304	4.285089928	0.020302175	http://www.ncbi.nlm.nih.gov/nuccore/NM_001243999.1
NM_001077243.2	mRNA	GRIA4	protein_coding	6.94E-17	6.94E-17	6.94E-17	2.117270109	5.059334702	0	4.296505696	0.021923949	http://www.ncbi.nlm.nih.gov/nuccore/NM_001077243.2
NM_001319198.1	mRNA	S100A8	protein_coding	6.94E-17	6.94E-17	6.94E-17	7.244379771	1.028273008	0	4.509215215	0.024032566	http://www.ncbi.nlm.nih.gov/nuccore/NM_001319198.1
NM_032094.1	mRNA	PCDHGA12	protein_coding	6.94E-17	6.94E-17	6.94E-17	6.234292345	0.011841252	2.703559597	4.637299553	0.007688693	http://www.ncbi.nlm.nih.gov/nuccore/NM_032094.1
NM_001319201.1	mRNA	S100A8	protein_coding	6.94E-17	6.94E-17	6.94E-17	9.309536794	1.031969616	0	4.828417126	0.01391762	http://www.ncbi.nlm.nih.gov/nuccore/NM_001319201.1
NM_001282868.1	mRNA	RHD	protein_coding	6.94E-17	6.94E-17	6.94E-17	4.205270357	3.055156154	3.622790834	4.909864914	0.001226998	http://www.ncbi.nlm.nih.gov/nuccore/NM_001282868.1
NM_001127691.2	mRNA	RHD	protein_coding	6.94E-17	6.94E-17	6.94E-17	4.205270357	3.055156154	3.622790834	4.909864987	0.001227097	http://www.ncbi.nlm.nih.gov/nuccore/NM_001127691.2
NM_001282869.1	mRNA	RHD	protein_coding	6.94E-17	6.94E-17	6.94E-17	4.205270357	3.055156154	3.622790834	4.909865808	0.001228215	http://www.ncbi.nlm.nih.gov/nuccore/NM_001282869.1
NM_001282872.1	mRNA	RHD	protein_coding	6.94E-17	6.94E-17	6.94E-17	4.205270357	3.055156154	3.622790834	4.909866586	0.001229276	http://www.ncbi.nlm.nih.gov/nuccore/NM_001282872.1
NM_001282871.1	mRNA	RHD	protein_coding	6.94E-17	6.94E-17	6.94E-17	4.205270357	3.055156154	3.622790834	4.909866601	0.001229296	http://www.ncbi.nlm.nih.gov/nuccore/NM_001282871.1
NM_001282870.1	mRNA	RHD	protein_coding	6.94E-17	6.94E-17	6.94E-17	4.205270357	3.055156154	3.622790834	4.909866698	0.001229428	http://www.ncbi.nlm.nih.gov/nuccore/NM_001282870.1
